# Global environmental drivers shape Cenozoic neoselachian diversity and identify modern conservation priorities

**DOI:** 10.1038/s41598-025-25653-6

**Published:** 2025-11-20

**Authors:** Manuel Andreas Staggl, Eduardo Villalobos-Segura, Michael J. Benton, Jürgen Kriwet

**Affiliations:** 1 Department of Palaeontology, Faculty of Earth Sciences, Geography and Astronomy, Josef-Holaubek-Platz 2, Vienna, 1090 Austria; 2 Vienna Doctoral School of Ecology and Evolution (VDSEE), University of Vienna, Djerassiplatz 1, Vienna, 1030 Austria; 3 School of Earth Sciences, University of Bristol, Wills Memorial Building, Queen’s Road, Bristol, BS8 1RJ UK

**Keywords:** Neoselachii, Biodiversity, Climate change, Conservation palaeobiology, Biodiversity, Biodiversity, Climate-change ecology, Conservation biology, Evolutionary ecology, Palaeoecology, Ichthyology, Palaeontology

## Abstract

Sharks, rays and their immediate extinct ancestors (Neoselachii) are some of the ecologically most impactful marine vertebrates. Despite their evolutionary success, the deep-time patterns of neoselachian diversity and turnover as a response to environmental perturbations remain understudied. Past studies have focussed on taxonomic richness while neglecting changes in faunal composition and ecological strategies, limiting our understanding of macroevolutionary resilience and vulnerability of neoselachians. Consequently, the extent to which major Cenozoic environmental shifts affected neoselachian diversity and turnover remain elusive. Here, we present a sampling-standardised analysis of Cenozoic neoselachian genus diversity and faunal composition change, using high-resolution fossil occurrence data and ecological and environmental variables. We demonstrate a milder impact of the KPg mass extinction on neoselachians than previously suggested, followed by a radiation culminating in an Eocene diversity peak, which is systematically documented and described for the first time using sampling-standardised data. Additionally, we identify two distinct faunal shifts during the Miocene. Shallow, heterogeneous habitats emerged as the primary diversification driver, although responses varied across ecological and taxonomic groups. These findings shed light on the evolutionary and ecological dynamics that shaped modern neoselachian diversity and provide critical context for assessing their vulnerability to ongoing anthropogenic pressures and informing conservation strategies.

**Supplementary Information:**

The online version contains supplementary material available at 10.1038/s41598-025-25653-6.

## Introduction

Neoselachii (modern sharks and rays together with their extinct immediate relatives (e.g. ♱Synechodontiformes^1^) have an extensive fossil record, reaching back to the Permian (~ 290 Ma)^2,3^. Mesozoic neoselachian diversity was initially low but increased dramatically, especially in the Late Cretaceous^4–7^. Today more than 1200 extant species of sharks and rays are known^8^, representing one of the most ecologically important vertebrate groups in today’s oceans^9^, triggering negative cascading effects in their respective ecosystems when disappearing^10–13^.

The Cenozoic, spans 66 million years, encompassing a broad array of environmental conditions^14^ including periods of exceedingly high temperatures during the early Eocene Climatic Optimum (~ 51 Ma), a series of consecutive cooling events, e.g. Eocene-Oligocene Transition (~ 34 Ma), and extensive Pleistocene (2.6 Ma) ice ages. While the KPg boundary event (66 Ma) is commonly regarded as a major extinction event for certain marine vertebrate groups^15^, its impact for neoselachians remains controversial, and its subsequent evolutionary consequences remain relatively unexplored^5,7,16–19^. Moreover, the extent to which environmental changes induced shifts in neoselachian faunal composition or diversity is not yet fully understood. Previous studies have largely focussed on overall neoselachian diversity trends^5,7,16–19^, often overlooking faunal or ecological turnovers, possibly indicating more nuanced environmental responses. Consequently, the present study seeks to address the following research questions: How did neoselachian diversity and faunal composition change throughout the Cenozoic? What were the driving forces underlying the observed patterns? Can knowledge of neoselachian diversification drivers contribute to our understanding of modern-day threats to neoselachians?

In this study we present a comprehensive sampling-standardised assessment of neoselachian genus-level diversity and faunal composition changes across the Cenozoic. By integrating abiotic and biotic environmental variables, we aim to identify the specific driving forces shaping neoselachian diversification and faunal turnover.

Our results indicate a milder KPg impact on neoselachians than previously suggested, followed by an all-time peak in diversity during the Eocene and two major Miocene faunal shifts - the most pronounced since the Early Jurassic. The availability of shallow, heterogeneous habitat emerges as the primary driver of Cenozoic neoselachian diversity and turnover, alongside secondary smaller scale factors affecting specific ecological and taxonomic subgroups.

Our results provide a foundation for a more refined perspective on the long-term resilience and ecological dynamics of neoselachians, as well as a deeper understanding of their evolutionary history and future vulnerability. Considering that over one third of modern shark and ray species are threatened with extinction^9^, understanding long-term drivers of neoselachian diversity and faunal composition is crucial for informing future conservation policies^20–23^.

## Materials and methods

### Fossil occurrence data and diversification analyses

#### Fossil occurrence dataset

The Staggl et al.^6^ dataset was used as the basis for the present analysis and extended by incorporating additional literature entries. To minimise uncertainties from misidentification of fossil taxa at the species level, we used genera as the study unit^16,21,24–27^. All data entries were meticulously reviewed and when necessary updated in terms of correct taxonomic assignment and validity following the taxonomy of the bibliography database Shark References^28^ (accessed between June 2023 and September 2024) supplemented by additional literature where needed. Only entries with age information (preassigned stage or geological formation) were considered, using the geological timescale of the International Chronostratigraphic Chart v2023/09^29^. For each occurrence, a minimum (last appearance date (LAD)) and maximum age (first appearance date (FAD)) was assigned, both not necessarily represent the actual time of origination and extinction of the taxon, but rather the known occurrence range^30^. A mid-point value between the maximum and minimum age of each occurrence was defined and subsequently utilised to allocate the respective occurrence to the relevant time bin according to this mid-point value^31^. This approach produced a final dataset of 49,750 occurrences, including 503 genera (Tab. S1), across 15 orders, spanning from the Permian to the Holocene. A rarefaction analysis was performed in R 4.3.2^32^ using the package Vegan (v.2.6–4.6.)^33^ at a CI of 0.95 to determine the sampling coverage and overall quality of the final dataset for the Triassic, Jurassic + Cretaceous, and Cenozoic periods (Fig. S1).

### Diversity analyses

Diversity dynamics were analysed in R 4.3.2^32^ using the package divDyn (v0.8.2.)^34^ using the function “*divDyn*”. Several subsets of the overall dataset were analysed using higher taxonomic ranks (e.g. orders) or similar ecological characteristics (e.g. habitat and lifestyle). Selachimorpha, Batomorpha, Carcharhiniformes, Lamniformes, Orectolobiformes and Squaliformes were analysed individually. Batoid orders were not analysed individually, as they encompass notably fewer occurrences than most shark orders, which could have led to rather inconsistent results when implementing sampling standardisation. We also ran analyses of shark clades by major habitat divisions: Carcharhiniformes and Lamniformes as “coastal sharks”; Heterodontiformes, Orectolobiformes, and Squatiniformes as “benthic sharks”; and Echinorhiniformes, Hexanchiformes, Pristiophoriformes, and Squaliformes as “deep-sea sharks”. These categories were assigned on the basis of ecological preferences of the individual groups set out in Ebert et al.^35^. Data visualisation was achieved via the R package ggplot2 (v.3.5.0.)^36^. To account for stage duration, the dataset was binned and analysed in 88 time bins of 4 Ma each^31,37,38^ (Tab. S2). A widespread problem in deep time diversity analyses is the “pull of the recent”^27^, however Pimiento & Benton^27^ showed this bias is not significant for the neoselachian fossil record. Aiming to further minimise this problem, we included only extant genera with fossil representatives. To diminish any potential additional edge effects towards the present, the binning was extended from the most recent bin (bin number 88) two million years into the future. To minimise sampling biases, shareholder quorum subsampling (SQS)^37,39^ was applied. For the neoselachian, selachian, and batoidean analyses, a quorum of q = 0.63 at 1000 iterations was applied. Quorum levels for the other groups include q = 0.8 for Carcharhiniformes, q = 0.59 for Lamniformes, q = 0.55 for Orectolobiformes, q = 0.357 for Squaliformes, q = 0.64 for benthic sharks, q = 0.58 for coastal sharks, and q = 0.63 for deep-sea sharks at 1000 iterations providing the most continuous results. To maximize the robustness of the diversity analyses, three approaches were utilized: sampled-in-bin (SIB), range-through (RT) and boundary-crosser (BC)^40^. The results of the diversity analyses are listed in Table S3.

### Faunal composition change

A detrended correspondence analysis (DCA) was performed (R 4.3.2^32^, with the function “*decorana*” in the package vegan (v.2.6–4.6.)^33^ following the method of Correa-Metrio et al.^41^, to track faunal composition change in deep time.

The positions of faunas per time bin, DCA axes 1 (DCA1) and 2 (DCA2) were plotted together (Fig. S2) as the highest relative importance was found in those two axes (Eigenvalues: DCA1: 0.74680; DCA2: 0.49910) (Tab. S4). In conjunction with the results of the diversity analyses, the values of DCA1 were employed as an additional dependent variable for the ensuing analyses.

### Diversification driver selection

To assess possible drivers of neoselachian diversity, we compared a selection of abiotic and biotic variables against neoselachian diversity and faunal turnover. The selection of the respective datasets was primarily based on criteria including actuality, completeness of data during the relevant period, and short binning of the individual data points. Linear interpolation was applied because of non-alignment of data points with the binning intervals of the diversity data (Tab. S3).

Data on abiotic environmental factors were obtained from the following sources: sea level, Miller et al.^42^; sea surface temperature, Song et al.^43^; deep-sea temperature, Meckler et al.^44^; atmospheric CO_2_ concentration, Hönisch et al.^45^; flooded continental area, Marcilly et al.^46^; continental fragmentation, Zaffos et al.^47^.

The following parameters were chosen as biotic factors possibly influencing neoselachian diversity due to their good fossil record and known relevance in marine ecosystems: dinoflagellate diversity^48^, calcareous nannoplankton diversity^48^, planktic foraminifera diversity^49^, and bony fish diversity (paleobiodb.org; Diversity metrics retrieved from the PBDB on 19 August 2023, search string: Actinopterygii).

### Environmental impact on neoselachian diversity patterns

The impact of several environmental predictors was determined by implementing a stepwise approach, which included an initial exploratory stage, followed by a subsequent quantitative analysis of the associations identified during the initial stage. The analyses of these dependencies were conducted using exclusively the results of the diversity analyses for the Cenozoic (time bin 72–88 Ma). Spearman’s rank correlation analyses were initially performed in R 4.3.2^32^ using the “cor.test” function to assess for general correlations between diversity and extrinsic factors (Tab. S5). The results of this pairwise correlation test were utilised for exploratory purposes, rather than being employed to derive explicit conclusions. Following the methodology of Marx & Uhen^50^, the relative probability for multiple a priori regression models was determined using Akaike weights and stepwise multivariate regression analysis in R 4.3.2^32^ with the packages divDyn (v0.8.2.)^34^ and ape (v.5.7-1.7.)^51^. Stepwise multivariate linear regression evaluates various model combinations to determine the best-fitting model. This method involves a stepwise search in both directions, initially adding variables and penalising poorly fitting ones, and then subsequently removing variables and reintroducing them if necessary. This process is repeated until the model that best fits the data is found. In each iteration, a variable is either added or removed, depending on its contribution to model enhancement^52^. The sampling-standardised diversity of neoselachians and the individual subsets and faunal turnover represented by DCA axis 1 (DCA1) were set as dependent variables respectively (Tab. S3). To mitigate potential impacts of abiotic factors on biotic factors, that might affect the results, separate analyses were conducted for both factor categories. Nonetheless, the potential for mutual interference between the two categories was explored and discussed qualitatively.

## Results and discussion

### Diversification patterns

**Fig. 1:**
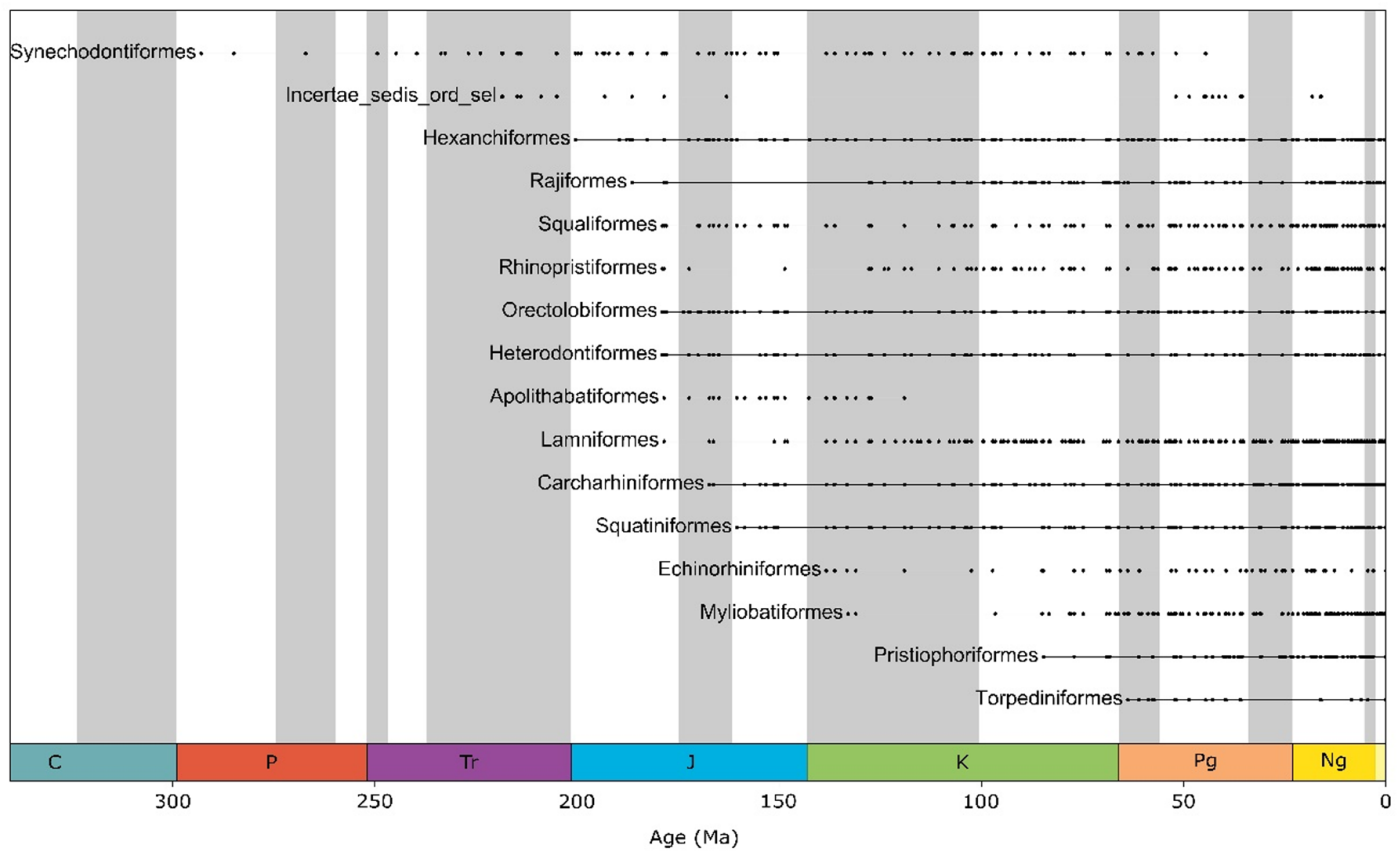
Chronostratigraphic range plot showing the emergence of neoselachian orders through time: Black dots represent one or more fossil occurrences at the respective time. The solid black bars correspond to the entire period between the first and last known occurrences of each taxon.

**Fig. 2:**
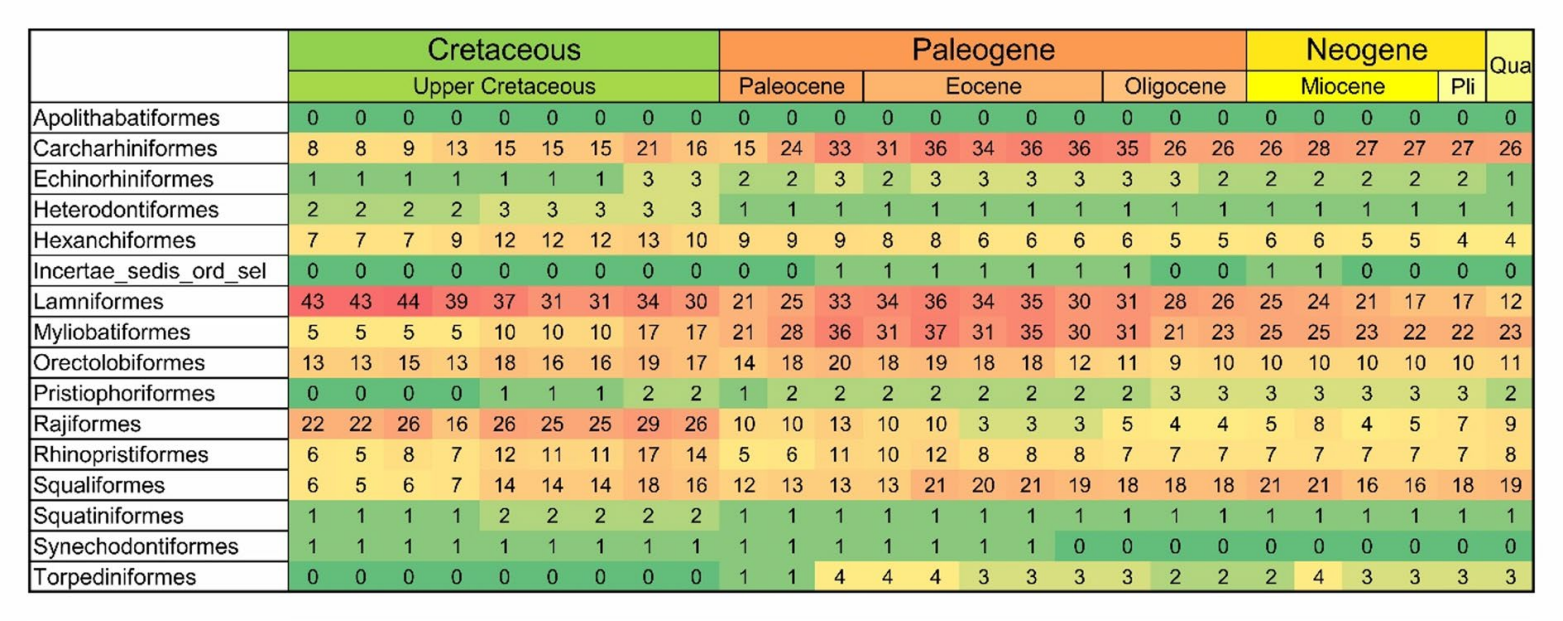
Heatmap of raw standing diversity of neoselachian genera per order in each time bin: Shading from green represents a lack of diversity, light yellow represents low diversity with an increase to red representing high diversity in the corresponding order in the respective bin (bins equal 4 Ma each).

The majority of extant neoselachian orders first appeared in the fossil record during the Jurassic (201 Ma) and persist to the present day, with the exception of Apolithabatiformes, known only until the Early Cretaceous^53^. In the Cretaceous (145 Ma), Echinorhiniformes^54^, Myliobatiformes^55^ and Pristiophoriformes^56^ made their appearances (Fig. 1). During the Cenozoic, many families particularly from the Carcharhiniformes and batoids (mainly Myliobatiformes) first appeared in the fossil record (Fig. S3). We found high standing genus diversity especially for Carcharhiniformes, Lamniformes, Myliobatiformes, Orectolobiformes and Squaliformes (Fig. 2).

**Fig. 3:**
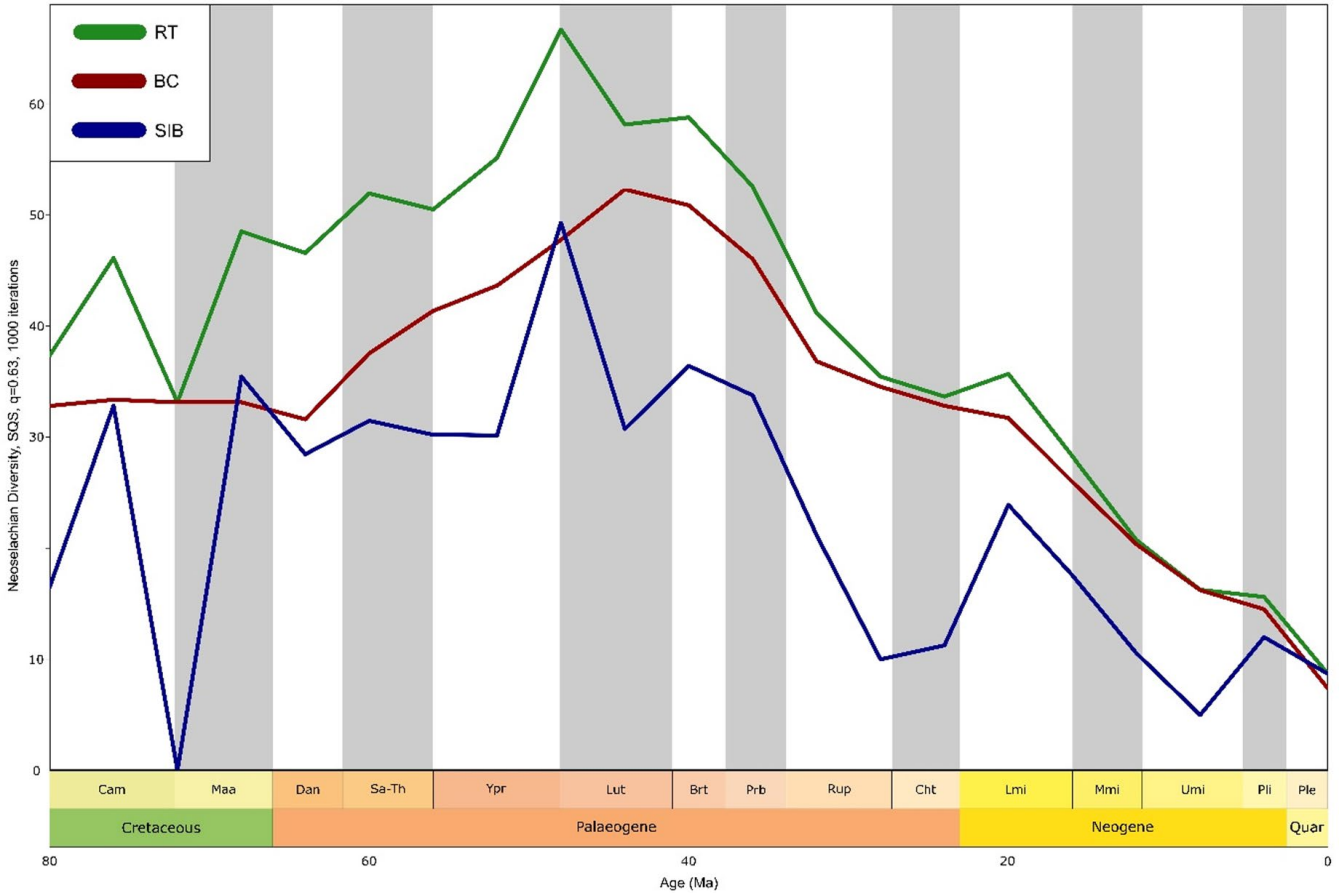
Diversity dynamics of neoselachian genera throughout the latest Mesozoic and the Cenozoic: Diversity is measured using sampling-standardised data (SQS, q = 0.63, 1000 iterations) by three complementary diversity approaches, sampled in bin (SIB, blue solid line), range through (RT, green solid line) and boundary crosser (BC, red solid line). Following a slight post-KPg decline, diversity remained relatively constant during the Palaeocene but started to increase during the Ypresian. After the peak at the Ypresian-Lutetian boundary, diversity declined until the present, interrupted only by periods of slight diversity increase in the late Eocene and early Miocene.

The results of the SQS analyses show variability in the quality of the resulting diversity curves (Fig. 3). Sampled-in-bin diversity (SIB) demonstrated a notably more volatile progression, as this approach is more sensitive to inconsistencies in the fossil record due to the inclusion of single-interval taxa^39,40^. The range-through (RT) and boundary-crosser (BC) curves produced more complete diversity curves, allowing for more insightful results when used as the dependent variables in correlations and regression models (described below).

**Fig. 4:**
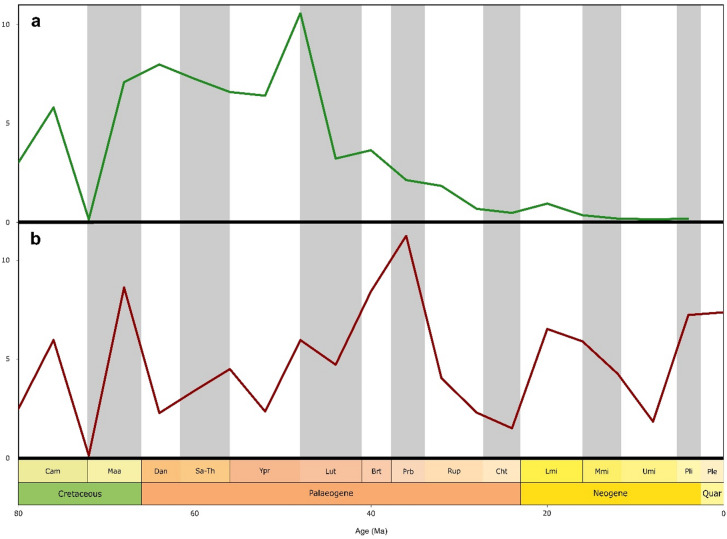
Latest Mesozoic and Cenozoic origination and extinction rates of neoselachian genera: Both diversity metrics are based on the subsampled data. **a**) Origination rate was high during the Palaeocene and early Eocene, peaked at the end of the Ypresian and subsequently dropped throughout the Lutetian. **b**) Extinction rate was relatively constant during the first part of the Cenozoic, but inclined notably throughout the late Eocene, dropped during the Oligocene and subsequently rose again in the early Miocene.

Our results for overall neoselachian diversity are similar to those reported by Friedmann & Sallan^25^ and Guinot & Condamine^19^, with only a slight decrease in diversity after the KPg boundary event (66 Ma). Our sampling-standardised results describe a pronounced neoselachian radiation with its origins in the mid-Danian (64 Ma) culminating in the mid-Palaeogene (48 Ma) (Fig. 3, Tab. S3). During the Ypresian (48 Ma) an increase of diversity can be observed peaking at the closure of this stage, and representing the all-time high in neoselachian diversity, which contrasts with earlier studies that established a peak in the Late Cretaceous^16–18^. Following this all-time peak, there is a pronounced decline in genus diversity that continues to the present day. Both trends (peak and decline) are also reflected in the origination and extinction rates (Fig. 4). The only notable phase of diversity increase after the Ypresian (56 Ma) peak, was during the early Miocene (23 Ma) (Fig. 3).

The sampling-standardised diversity curves for subsets of the data present similar patterns, especially for sharks (Fig. S4) and batoids (Fig. S5). However, certain curves differ in certain respects. Lamniformes (Fig. S6) and Carcharhiniformes (Fig. S7) exhibit comparable tendencies, both separately and combined (Fig. S8) aligning generally with the neoselachian SQS curve, which is to be expected as they represent most of the entries in the dataset. Lamniformes exhibit a prolonged phase of high standing diversity during most of the Eocene and a somewhat delayed onset of diversity decline after the Eocene peak, in contrast to Carcharhiniformes. This order exhibits a pronounced decline following the Ypresian–Lutetian boundary (48 Ma), which persists through the remainder of the Eocene. As shown by the gap in the SQS curve during the Oligocene, this order is underrepresented in the fossil record during this epoch.

Heterodontiformes, Orectolobiformes, and Squatiniformes (benthic sharks) show no immediate decline in diversity in the aftermath of the KPg boundary event but experience a notable drop starting at 60 Ma (Fig. S9). During the Eocene, diversity increases again and peaks at the end of the Ypresian, being followed by a steady decline until the middle Oligocene (27 Ma). Since then, diversity remain roughly constant until the present day. The same pattern is observed when looking at the carpet sharks (Orectolobiformes) separately (Fig. S10).

Deep-sea sharks diverge from general patterns (Fig. S11), showing stable diversity after a marked post-KPg decline and a radiation peak in the Bartonian (41 Ma), followed by a brief decline until the Priabonian (37 Ma) and subsequent stable phase until the present. Squaliformes, representing most deep-sea shark occurrences in our dataset, mirror this trend (Fig. S12). Comparably to Carcharhiniformes, they exhibit an Oligocene sampling gap and more dynamic Neogene (23 Ma) diversity.

Even though some variation between the various subsets exists, overall similar patterns are recovered, showing pronounced Eocene peaks followed by declines in diversity. Plots depicting the raw diversity for neoselachians, sharks and batoids, respectively, can be found in the supplementary data (Fig. S13).

### Faunal composition change

**Fig. 5:**
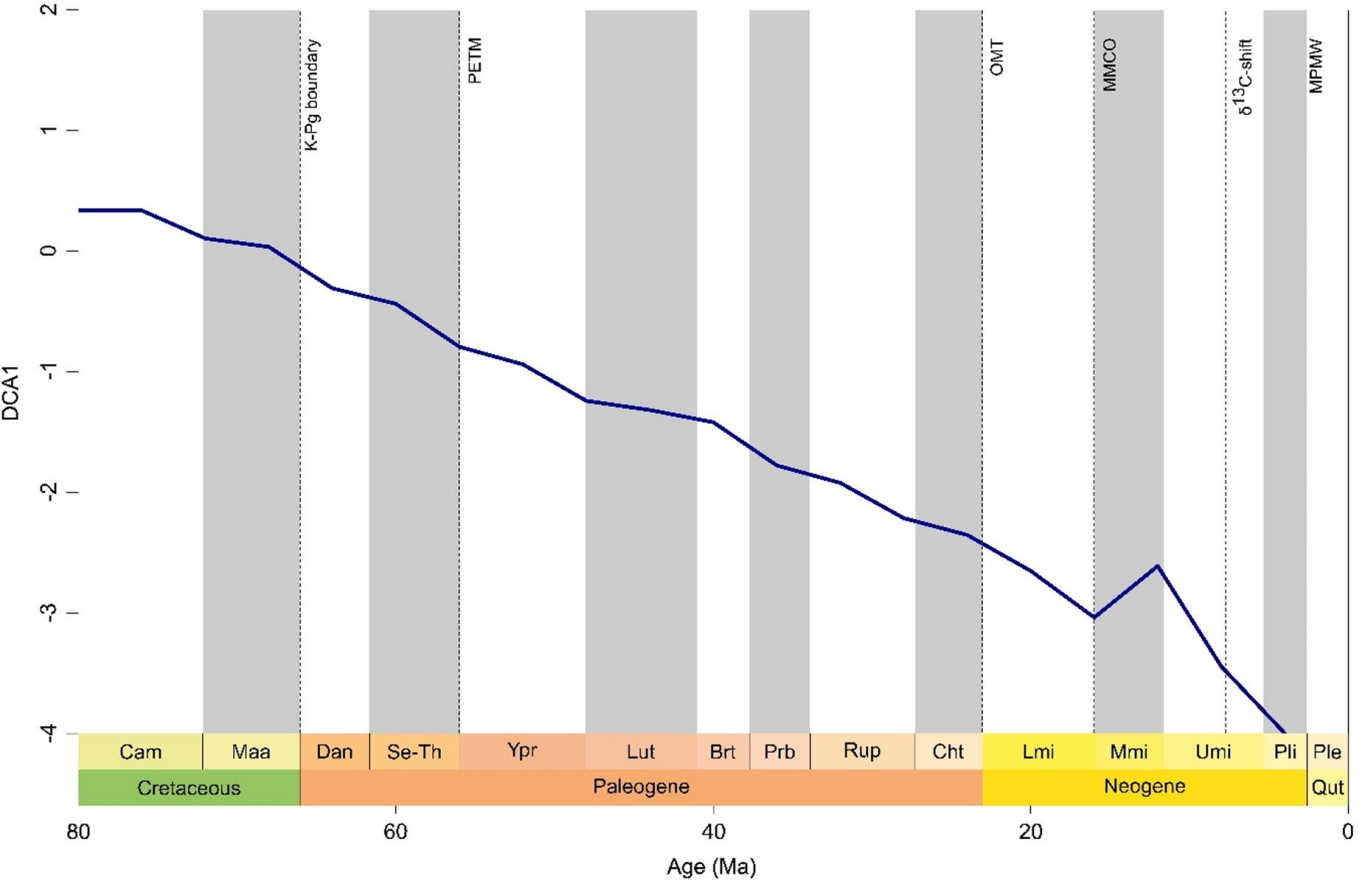
Neoselachian faunal composition change (DCA axis 1) throughout the latest Mesozoic and the Cenozoic: Blue solid line shows the rate of faunal change as a function of its ordination derived from DCA rescaled faunal composition. Y-axis units are standard deviation as a proxy for faunal turnover. Marked events in Earth’s history are indicated by dashed lines for reference. Abbreviations: MMCO, mid Miocene climate optimum; MPWP, mid Pliocene warm period; PETM, Palaeocene–Eocene thermal maximum; OMT, Oligocene-Miocene climatic transition; δ13C-shift, Carbon (δ^13^C) isotope shift.

Despite overall constant faunal turnover, the detrended correspondence analysis (DCA) (Figs. 5, S2 Tab. S4) reveal two major faunal shifts (alterations in genera per order) (Fig. S14) - one at the early to middle Miocene boundary (16 Ma) and the other at the middle to late Miocene boundary (12 Ma), making this the first major deviations since the Jurassic (see Staggl et al.)^6^. The faunal shift during early and middle Miocene is observed especially among deep-sea sharks (Fig. S15) and batoids (Fig. S16), and the middle to late Miocene boundary shift is particularly strong among coastal sharks (Fig. S17).

### Drivers of Cenozoic neoselachian diversity and faunal composition

We found several extrinsic factors with recurring correlations among the different subsamples and diversity approaches. Overall, the strongest correlations occurred with flooded continental area, CO_2_ concentration, continental fragmentation, and dinoflagellate diversity (Tab. S5). By applying stepwise multivariate regression analysis, we have been able not only to engage in a qualitative discussion of possible evolutionary drivers, but also to add a quantitative component to it.

### Abiotic drivers

**Fig. 6:**
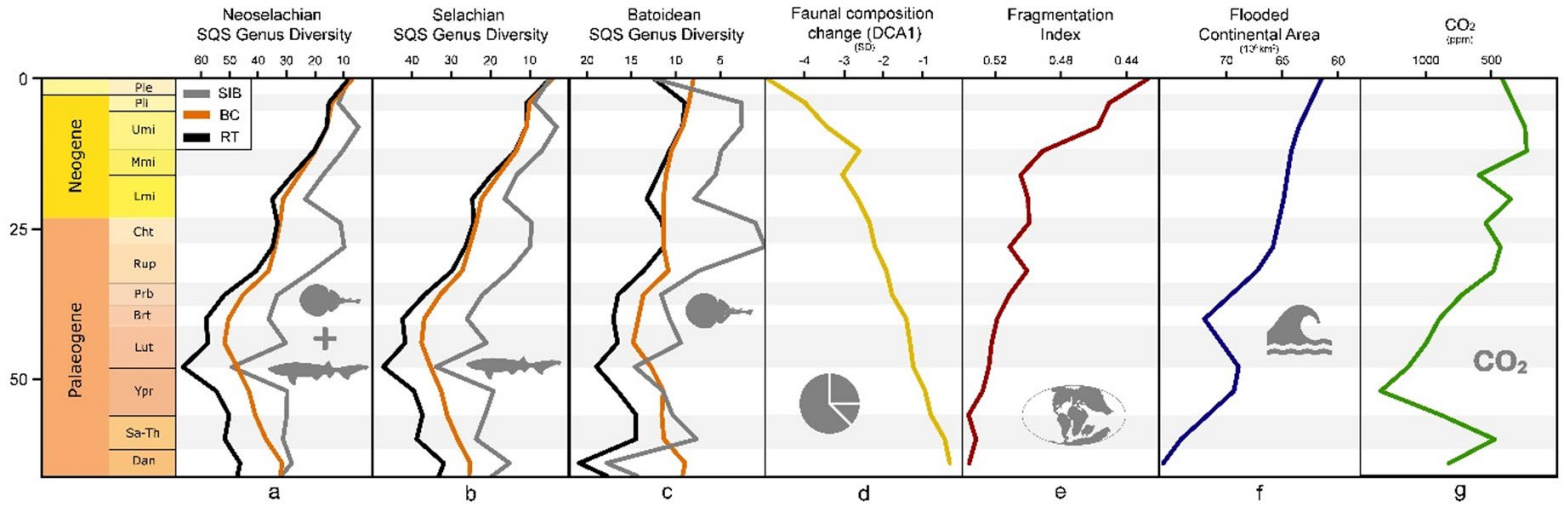
Comparison of neoselachian **a**), selachian **b**), batoidean **c**) genus diversity, and neoselachian faunal composition change **d**), with continental fragmentation **e**), flooded continental area **f**), and atmospheric CO_2_ concentration **g**) through time. Diversity in a), b), c) is shown as sampling standardises (SQS) sampled-in-bin (grey), boundary-crosser (orange), and range-through (black) diversity. Data of e), f), g) retrieved from published literature, respective reference mentioned in the material and method section. For full statistical results see Table S5.

Stepwise multivariate linear modelling identified the best fitting model of abiotic factors for each investigated (sub)group and diversity metric (Fig. 6, Tab. S6). For RT and BC diversity, continental fragmentation and atmospheric CO_2_ concentration explained most of the variance (75.3% and 84.7% respectively). SIB diversity is influenced by continental fragmentation, sea level and sea surface temperature (*p* > 0.05, not significant), explaining 71.8% of the variance. Sampled-in-bin models were significant but explained less variance than RT and BC models. We found the combination of continental fragmentation and the extent of flooded continental area as the most important abiotic drivers of neoselachian faunal composition change (Tab. S6). These two factors explain (together with sea level and sea surface temperature, both not significant (*p* > 0.05)) over 95% of the observed variance in neoselachian faunal composition change during the Cenozoic and thus highly influencing neoselachian diversity. This resembles the results found for the Mesozoic faunal turnover drivers described by Staggl et al.^6^.

The best model fits for most of the individually assessed subsamples of the full dataset appear in similar combinations as for overall neoselachian diversity, albeit with some variation in distinct groups. Deep-sea shark diversity benefits from higher continental fragmentation too, but additionally from lower deep-sea temperature, suggesting a preference for cooler conditions—consistent with previous findings^57–59^.

The most significant abiotic diversification driver for benthic sharks is the amount of flooded continental area (epicontinental sea area), further emphasising the preference of these taxa for shallow marine habitats.

Coastal sharks, including Carcharhiniformes and Lamniformes, exhibit a marked benefit from higher continental fragmentation, contrary to the findings of Condamine et al.^60^. This discrepancy may be attributed to differences in taxonomic resolution or the modelling framework applied in the respective analyses. When analysed separately, both groups are additionally influenced by atmospheric CO₂ concentration. The results for carcharhiniforms lack significance, which is probably explained by a gap in the SQS diversity data due to uneven sampling during the Oligocene (34 Ma).

During the Miocene (23 Ma), several distinct environmental changes occurred including climatic perturbations and tectonic processes resulting in several major changes in marine ecosystems. To some extent, these processes are interdependent and therefore difficult to consider separately^61^.

The most important factor for neoselachian diversification and faunal turnover is higher continental fragmentation which results in greater habitat heterogeneity, formation of dispersal barriers, and niche partitioning promoting allopatric speciation^38,47,62–64^, and eventually balancing competition and leading to rather gradual turnovers^65^. The global decline in shallow marine area throughout the Cenozoic^66^ parallels the overall decline in diversity observed here. It is widely accepted that a gradual increase in habitats due to the rise in flooded continental areas is accompanied by the emergence of new taxa^18,38^. Conversely, a decrease in habitat area leads to extinction in various scales^18,38^. While not statistically significant, both sea level and sea surface temperature influence the observed associations of faunal turnover and shallow marine habitat (epicontinental sea areas) availability by also promoting more heterogeneous habitats and the expansion of warm shallow marine habitats^62^. The two distinct shifts in faunal composition during the Miocene (Fig. 5), are the most notable shifts since the Early Jurassic^6^, and align with the timing of the final closure of the Tethys Seaway^66^.

During the Cenozoic, scleractinian reefs became increasingly important as shallow marine ecosystems because of their elevated species richness resulting from their habitat complexity^67,68^. Nowadays, hotspots of neoselachian diversity, both taxonomically and functionally, also occur in tropical coral reefs, i.e. in shallow marine areas^69,70^. These areas are also of immense value for species that tend to live in more open parts of the ocean and are therefore usually considered to be pelagic, like many lamniform sharks and some carcharhiniform sharks as they represent in general areas of high productivity^69^. This suggests, in combination with the results of our analysis, that shallow marine habitats were already of foremost importance for these animals in deep time.

Steinthorsdottir et al.^61^ reviewed geological and biological processes during the Miocene, but only briefly mentioned neoselachians. They showed that the closure of the Tethys Seaway, the onset of the closure of the Panama Isthmus, and the widening of the Fram Strait and Drake Passage, promoted the diversification of neoselachians by creating or removing dispersal barriers and increased nutrient availability in the deep-sea and many coastal areas through increased circulation in parts of the ocean. Thus, confirming the significance of alterations in ocean connectivity, not only through the emergence or closure of seaways, but also through the subsequent establishment of key ocean current systems and deepwater bodies.

Higher continental fragmentation also might increase habitat heterogeneity in deeper water environments^62^, enabling the diversification of deep-sea sharks (Tab. S6). The dependence of this group on their habitat is further underlined by the association with lower deep-sea temperatures. A strong increase in sea surface temperature can be observed along the diversity increase in the Eocene (Tab. S3), similar to that recovered by Marion et al.^59^ for squaliform sharks.

Squaliform sharks represent a large portion of the occurrences within the category of deep-sea sharks and therefore present a strong correlation with the general signal found for that category in the present study. The reciprocal relationship between deep-sea temperature and deep-sea shark diversity is not only evident from the multivariate linear modelling, but also from the comparative assessment of both parameters through the Palaeocene (66 Ma) and Eocene (56 Ma) (Tab. S3). The observed reciprocal relationship suggests that the decline in deep-sea temperature may have further played a pivotal role in fostering diversification of these taxa in the deep-sea. Given the documented increase in diversity seen among deep-sea sharks (Fig. S11), we hypothesise that they might have avoided elevated sea surface temperatures in shallow waters throughout the early Cenozoic by migrating to greater depths or higher latitudes^71^. Marion et al.^59^ found several shifts of various squaliform taxa from shallow environments to the deep-sea, but suggested a higher probability for taxa being already in the deep-sea after the KPg boundary event than a *de novo* colonisation of these ecosystems after the event. The question of whether the cooler temperatures had a direct physiological effect on the taxa or whether the secondary effects of decreasing temperatures on the deep-sea ecosystem and thus on these sharks were responsible for the observed relationship is still poorly understood. Cenozoic squaliform diversification has been attributed^57,72^ to opportunistic occupation of freed niches following the reorganisation of the ecosystem in the aftermath of the KPg boundary event. The rather generalist nature of squaliform lifestyle and their resulting adaptability suggest this to be a reasonable scenario^72^. Marion et al.^59^ further suggested a squaliform turnover at the Eocene-Oligocene transition (34 Ma), we cannot confirm this, but our results found instead two massive turnovers in the early Miocene (Fig. S13) suggested by a change in DCA values of four standard deviations. This faunal composition changes represent two complete turnovers in deep-sea sharks within less than 15 million years (Fig. S14, Tab. S4)^73^. This is likely caused by a sampling artefact, as deep-water deposits are scarce, and few are accessible to study^57^.

The majority of modern carcharhiniform and lamniform sharks is either highly associated or at least in partially dependent on coastal, shallow water habitats^35^. Certain deep-dwelling carcharhiniforms (e.g. Pentanchidae) and some pelagic carcharhinids (e.g. *Carcharhinus longimanus*, *C. falciformis*, *Prionace glauca*) and lamniforms (e.g. *Isurus oxyrinchus*) can be considered exceptions, while many other pelagic forms spend considerable time in shallower areas for hunting and/or reproduction (e.g. *Carcharodon carcharias*, *Cetorhinus maximus*, etc.)^35^. Therefore, it appears reasonable to conclude that the availability of heterogeneous shallow-water habitats exerts a major influence on the success of these two taxonomic groups.

We found that CO_2_ concentration was the second most significant factor influencing overall neoselachian, but also carcharhiniform and lamniform diversity patterns. The positive association with CO_2_ may be indirect via increased primary production and hence increased productivity of the ecosystem as a whole^74^. Carbon dioxide as a greenhouse gas leads to global warming^75^, which contributed during the Cenozoic to comparably warmer conditions on Earth, causing tropical and subtropical regions to expand further north and south^21,76^. The warm phase around and during the Miocene Climate Optimum (∼16.9–14.7 Ma)^61^ facilitated, for instance, low latitude and therewith rather warm adapted communities to expand further North and South. This led together with the progressive closure of the Tethys Seaway, to a distinct shift in global biodiversity hotspots^66^, allowing for example warm adapted plankton and scleractinian reefs to occur at higher latitudes^61^. The favouring of these plankton taxa, together with the general trend of planktonic foraminiferans shell size increase^61^, was associated with enhanced nutrient transport to higher trophic levels, which in turn supported a more diverse and abundant meso- and apex predator level^77^. For a more detailed discussion on the importance of primary production on neoselachian diversification, please refer to the section on ‘Biotic drivers’ below. Staggl et al.^6^ found conversely a negative association of CO_2_ concentration and diversity during the Jurassic and Cretaceous and hypothesised a possible negative physiological effect of elevated CO_2_ levels on neoselachians, already shown for extant taxa^78^. CO_2_ levels in the Mesozoic were in most parts higher than in the Cenozoic^79^; therefore, it is probable that the positive effects of CO_2_ (e.g. enhancing primary production) were outweighed by the negative effects. Conversely, in the Cenozoic, the beneficial effects may have prevailed, which is why a positive association with neoselachian diversity is recovered.

Temperature has been identified as a major factor influencing the evolution of some neoselachian orders and particularly lamniform and carcharhiniform sharks. The climate cooling during the Cenozoic has been hypothesised as a cause for the decline in lamniform shark diversity^60^. However, it remains unclear why sea surface temperature was not a significant driver of diversity in the best model fit or in the correlations in our study. One potential explanation is the relatively pronounced differences in temperature ranges over the last 66 Ma, which present fundamentally different challenges for the prevailing fauna.

Taxa adapted to shallow water environments generally exhibit a higher tolerance to warmer temperatures^80^. Their increased motility likely allowed them in earlier times to migrate to habitats with a favourable thermal range, as observed today^71^. This pattern is evident in marine taxa in the context of climate change and has also been suggested for some fossil taxa^7,63,71,81–83^. Opportunistic migration along temperature gradients can only be determined geographically over time by tracking fossil occurrences. This flexibility of possible migratory behaviour along the preferred temperature gradients could impede the statistical identification of water temperature as a driving factor^71^.

Nevertheless, temperature also affects other parts of the marine food web and may have an indirect impact on neoselachian diversity. For instance, ocean cooling beginning in the Oligocene led to a gradual decline in coral diversity^66^, which provided essential habitats for many neoselachian groups^84^. This decline was a primary factor in the loss of the Western Tethys hotspot, which likely was also of major importance for neoselachians^66^. Conversely, elevated temperatures might also pose challenges. Yasuhara et al.^66^ demonstrated that the Western Tethys was by no means the warmest region during the warm Eocene episode but nevertheless had the highest diversity. This suggest that the equatorial regions were probably too warm at that time, resulting in lower diversities. Consequently, temperature emerges as a pivotal factor that exerted both direct and indirect influences on the evolutionary trajectory of neoselachians. However, the extent to which this phenomenon manifests in our quantitative analyses is limited, prompting a discussion that primarily employs qualitative approaches to explore temperature’s role as a key driver from an ecological viewpoint.

### Biotic drivers

Dinoflagellate diversity was consistently identified as a diversification driver (Tab. S7), across the different taxonomic and ecologic subgroups studied. Dinoflagellates are important primary producers in open oceans as well as in coastal regions^61^. Like sharks and rays, dinoflagellates experienced a decline in diversity throughout the Cenozoic, attributed to the reduction of shelf areas^85^. This mirrors our findings for neoselachians and indicate that these primary producers responded to environmental perturbations in a similar way as neoselachians.

Two planktonic groups turn out to be significant factors influencing neoselachian faunal turnovers (Tab. S7): Calcareous nannoplankton and planktonic foraminiferans. We found a negative association between neoselachian faunal turnover and calcareous nannoplankton diversity, meaning higher diversity in this group results in lower turnover values. Conversely, there is a positive association with planktonic foraminiferans, indicating higher turnover rates with greater foraminifera diversity. The complex mechanisms behind this relationship are far-reaching and not always comprehensibly understood^86^. However, it is evident that these two groups of primary producers play a pivotal role in both past and present ecosystems, exerting significant, albeit indirect, influence on higher trophic levels, including sharks and rays. Lower trophic levels are similarly impacted by environmental change, as previously demonstrated by the influence of submerged shelf area and temperature on dinoflagellates^85^. Consequently, it is impracticable to entirely uncouple the relationship between neoselachian diversity/turnover and plankton diversity from abiotic factors. Instead, it is beneficial to contextualise and discuss abiotic processes such as plate tectonics, ocean currents and temperature changes and their consequences in relation to observed biological/ecological processes^60,86–88^.

Competition is often assumed to be a key driver of biotic diversification^38^. However, demonstrating biotic or competitive relationships in ecological systems poses a considerable challenge due to the numerous potential links that can be established^89,90^. The issue is further complicated by the introduction of the temporal dimension inherent in palaeontological studies, and the incompleteness of the fossil record. Therefore, we adopt a qualitative approach when discussing possible competitive relationships of Cenozoic neoselachians with other marine groups.

A notable difference between Cenozoic and Mesozoic marine faunas is the absence of large predatory marine reptiles during the Cenozoic^91^. This group has been previously identified as potential competitors and possible prey for Mesozoic sharks and rays (e.g. Benton^15)^. The vacated niches of these extinct marine reptiles may have been filled by neoselachians and larger bony fish taxa after the KPg boundary event. This enabled them, along with large bony fishes and marine mammals, to occupy high trophic levels in their respective ecosystems^15,91^.

Another group exerting influence on neoselachians is the Neoselachii themselves. Sharks of different orders (Lamniformes and Carcharhiniformes) have been identified as having a considerable influence on the evolution of other groups^92^. However, we have not found statistical evidence for a negative association between lamniform and carcharhiniform sharks in terms of taxic diversity. We observed a high positive correlation between these two orders (i.e. LamRT ~ CarRT: Rho = 0.946, *p* = 9.654E-09; LamBC ~ CarBC: Rho = 0.855, *p* = 1.203E-05; LamSIB ~ CarSIB: Rho = 0.802, *p* = 1.097E-04; for further details Tab. S5), suggesting a mutual positive relationship. However, this likely indicates they are being influenced by the same parameters, which confirmed by the stepwise multivariate linear regression (RT: Fragmentation index (p_Carcharhiniformes_ > 0.05), CO_2_). Condamine et al.^60^ reported a potential positive interaction of some taxa within these two orders, driving their evolution and diversification. The dynamic interplay between different neoselachian orders highlights the potential for reciprocal evolutionary influences within this diverse group.

The Cenozoic and particularly the Eocene is marked by the onset of a strong radiation of bony fishes^17,25,91,93–96^. Following the KPg boundary event, a considerable number of new lineages arose^17^ including numerous relatively small taxa, but also a few larger apex predatory taxa, such as large scombroids, carangids, xiphioids, and sphyraenids^15,17,91,97^. The probability of intense competition, particularly among smaller taxa, is considered low. Nevertheless, the potential value of these taxa as a food source could be substantial^98^. This is particularly evident in certain neoselachian groups that are typically regarded as teleost predators, such as many carcharhinids and lamnids, which diversified during the Cenozoic^99,100^. Nevertheless, we observed only very minor correlations across all diversity metrics and neoselachian subgroups (Rho = −0.3 to 0.3), which are not statistically significant (*p* > 0.05) (Tab. S5). This suggests a rather limited impact of Cenozoic bony fish diversification on neoselachian taxonomic diversity.

## Conclusion

Our study provides new insights into the diversity patterns and fluctuations in faunal composition of neoselachians during the Cenozoic, a significant chapter in Earth’s history characterised by a diverse array of environmental conditions. We found that the KPg had a milder effect than assumed, followed by a remarkable early Eocene radiation and peak diversity with a general long-term decline up to the present. Faunal compositional changes remained remarkably constant across the KPg boundary until two marked Miocene shifts—the most notable since the Early Jurassic. Diversity was primarily driven by the availability of shallow, heterogeneous habitats, with subgroup-specific variations reflecting ecological preferences.

Rising sea levels, CO_2_ concentrations, and sea surface temperatures may superficially suggest favourable conditions for neoselachians, however, rapid climate change imposes novel pressures on sharks and rays, especially through secondary ecosystem effects. While neoselachians have been shown to be highly adaptive in the past, the vulnerability of highly specialised taxa (e.g., deep-sea sharks adapted to cold, stable environments) will remain a major conservation concern^101^. Our results suggest that this group may have colonised deeper habitats or higher latitudes in response to past warming events, but the extreme pace of today’s ocean warming may impede the ability of such adaptive responses.

Overfishing continues to be the greatest threat to extant neoselachians, with more than a third of species currently evaluated as threatened with extinction (primarily due to unsustainable fishing)^9^. This overexploitation compromises their resilience and adaptability. Additionally emerging threats like climate change, habitat loss and degradation additionally pressures many taxa being already stressed by overfishing^9^. Given their role as apex- and meso-predators, the conservation of sharks and rays is of paramount importance not only in terms of biodiversity conservation, but also for maintaining the overall stability of marine ecosystems also as a basis for global food security^9,102^.

Future research addressing the role of ecological interactions, including predator-prey interactions, competition, and the integration of trophic data, functional traits, and palaeoenvironmental reconstructions could additionally offer better insights into both bottom-up and top-down processes in Cenozoic marine food webs further elucidating the factors shaping neoselachian diversity and faunal composition. While the present study has considered key uncertainties, such as the incompleteness of the fossil record, it should be noted that the results are sensitive to the choice of parameters and input data. Our results thus highlight the importance of further exploring the potential of additional predictors, and further investigations using regional datasets could shed light on macroevolutionary processes for unique geographic regions.

In the face of increased anthropogenic influences, a deep time understanding of extrinsic factors that drove, and sustained neoselachian diversity patterns and faunal turnovers provides a valuable framework for predicting, and thus potentially mitigating, future impacts on neoselachians.

## Supplementary Information

Below is the link to the electronic supplementary material.

Supplementary Material 1

## Data Availability

The data that support the findings are available in the supplementary material. The dataset underlying this research will be made available following an embargo period and will be accessible via the figshare repository under https://doi.org/10.6084/m9.figshare.c.8042509. A temporary embargo applies to the dataset, as it forms part of an ongoing doctoral project.

## Code availability

R programming codes are based on previously published methods and will be made available following an embargo period and will be accessible via the figshare repository under 10.6084/m9.figshare.c.8042509.
