# Supplementary material for: Global environmental drivers shape Cenozoic neoselachian diversity and identify modern conservation priorities
Source: Sci Rep. 2025 Nov 20;15:38661. doi: 10.1038/s41598-025-25653-6 (PMC12635402; doi:10.1038/s41598-025-25653-6)
Supplement: Supplementary file 1 — Supplementary Material 1 [file 41598_2025_25653_MOESM1_ESM.pdf]

Supplementary Materials, Figures

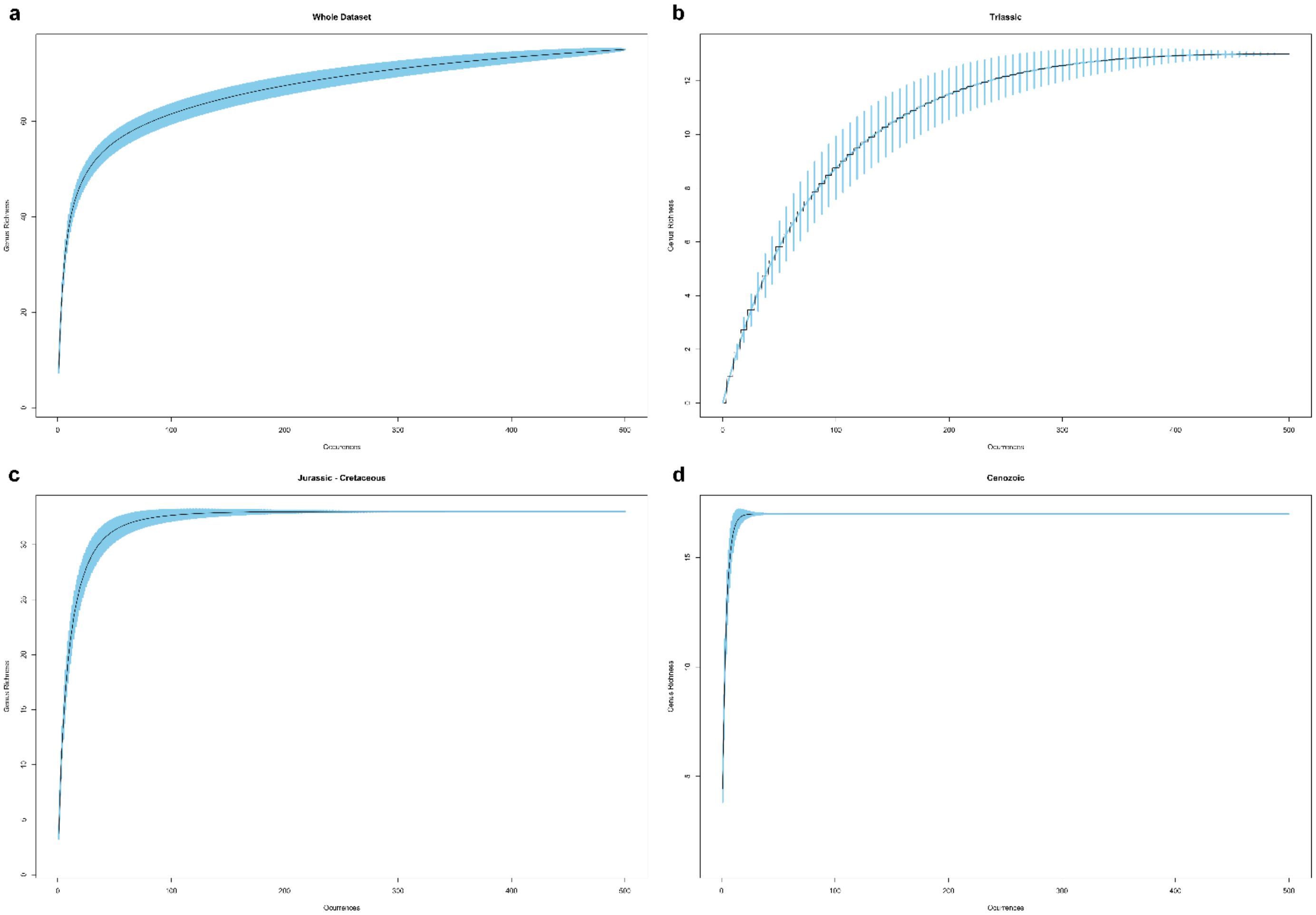

Figure S1: Rarefaction curves of genus richness for neoselachian fossil assemblages across time, with 95% confidence intervals derived from 1000 permutations. Blue shading indicates the confidence interval: a) the complete dataset, b) the Triassic, c) the Jurassic and Cretaceous combined, and d) the Cenozoic.

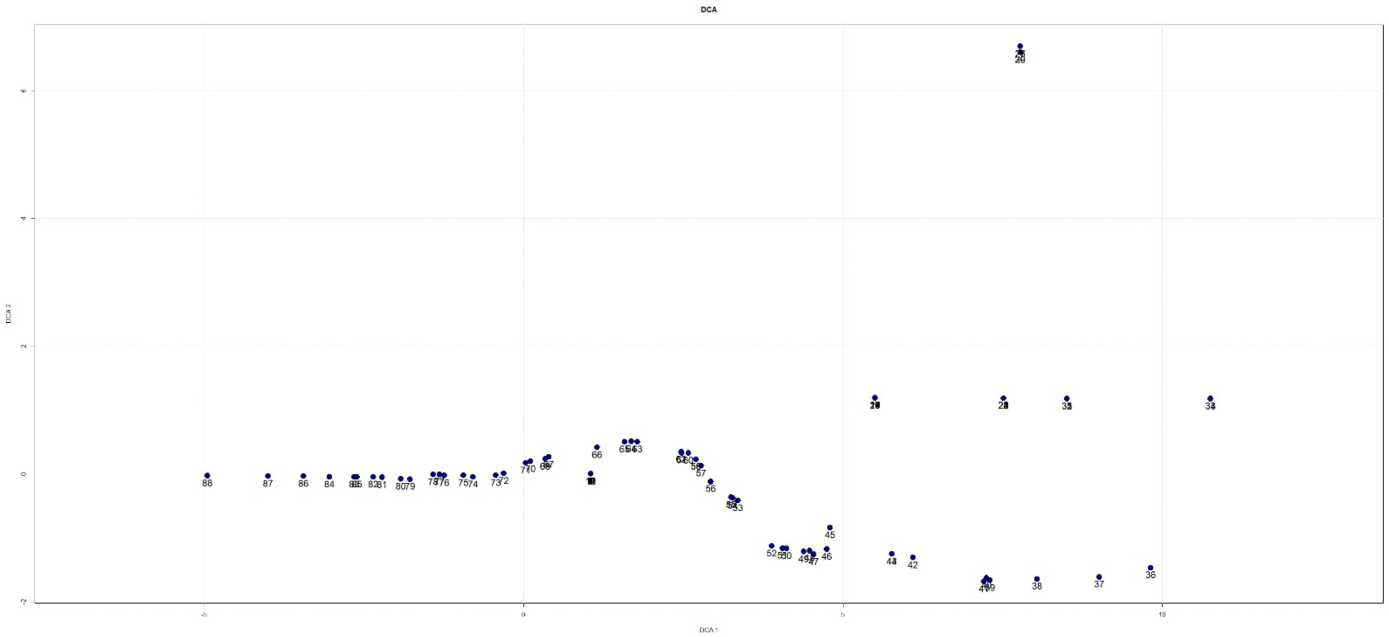

Figure S2: Ordination of neoselachian assemblages based on DCA axes 1 and 2, illustrating temporal shifts in faunal composition. Blue points on the ordination space represent the centroids of a time-specific assemblage (faunal composition). Axes represent the magnitude of compositional turnover, measured in standard deviation units. The numerical values assigned to each point correspond to the respective time bin.

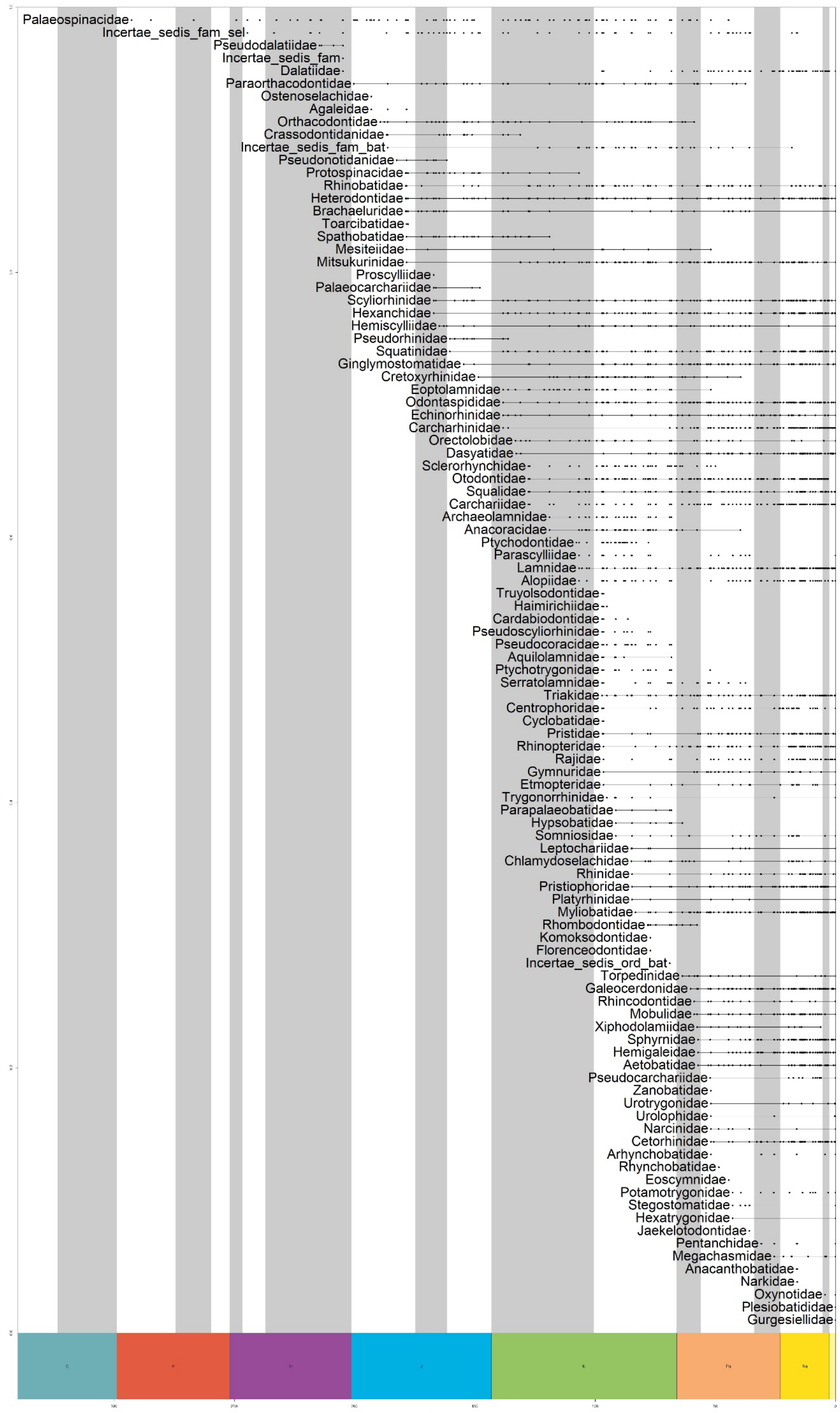

Figure S3: Chronostratigraphic range chart of neoselachian families. Black points indicate individual fossil occurrences attributed to their respective families, while horizontal black bars represent the stratigraphic range from the earliest to the latest recorded occurrence.

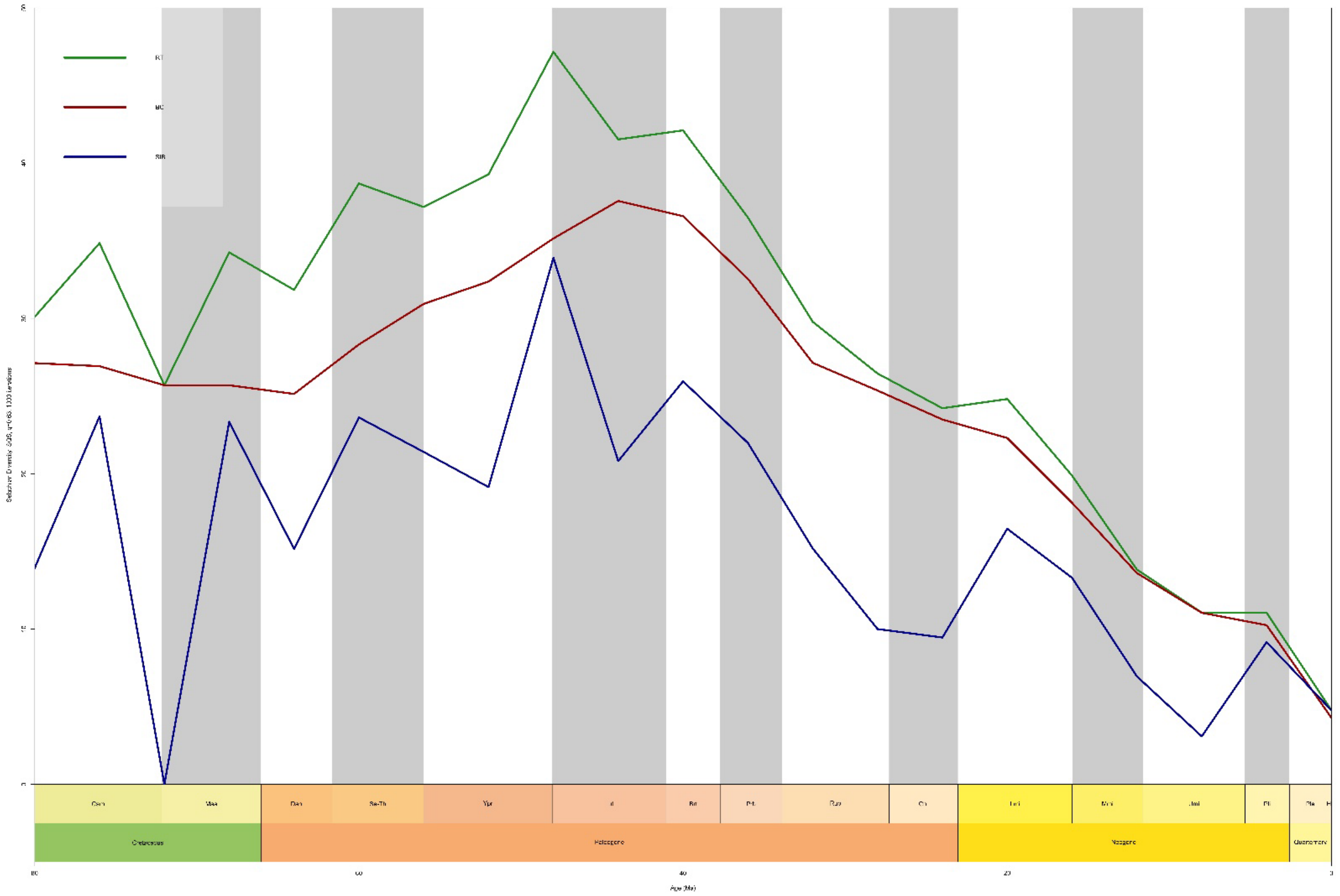

Figure S4: Selachian genus-level diversity trajectories across the latest Mesozoic and Cenozoic, inferred from shareholder quorum subsampling (SQS; quorum level q = 0.63, 1000 iterations). The three diversity measures used are illustrated accordingly: Sampled-in-bin (SIB, blue line), boundary-crosser (BC, red line), and range-through (RT, green line).

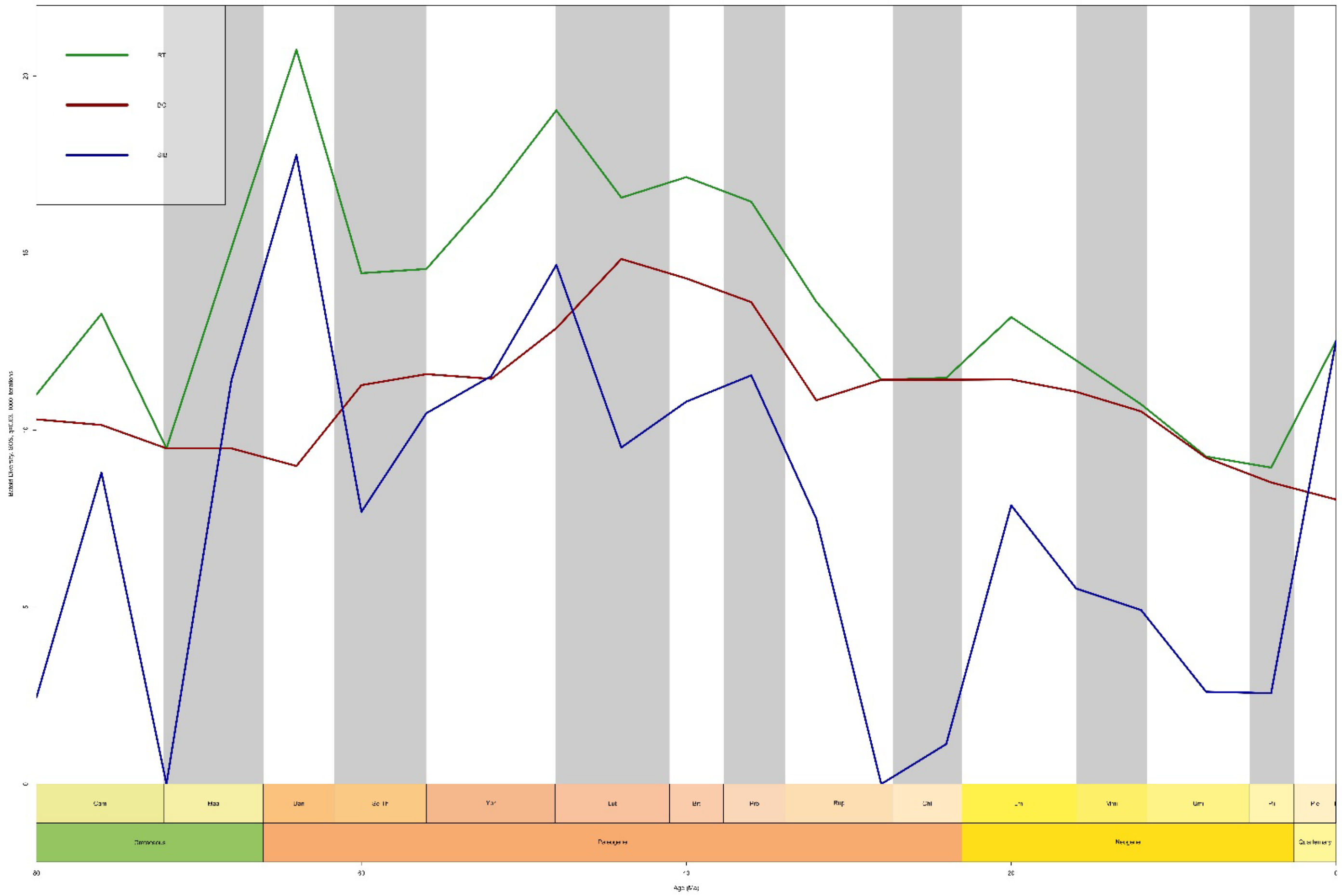

Figure S5: Batoidean genus-level diversity trajectories across the latest Mesozoic and Cenozoic, inferred from shareholder quorum subsampling (SQS; quorum level q = 0.63, 1000 iterations). The three diversity measures used are illustrated accordingly: Sampled-in-bin (SIB, blue line), boundary-crosser (BC, red line), and range-through (RT, green line).

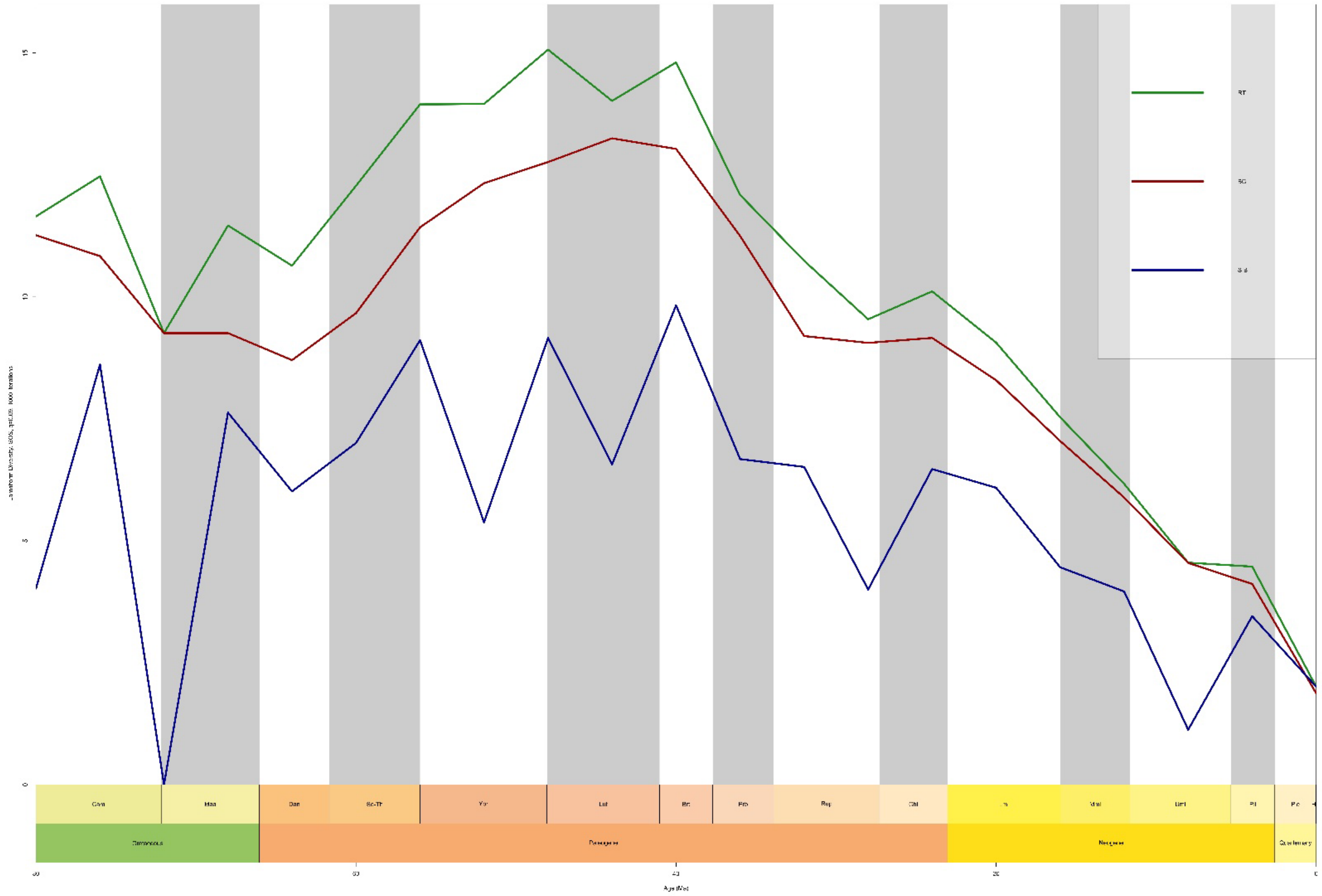

Figure S6: Lamniform genus-level diversity trajectories across the latest Mesozoic and Cenozoic, inferred from shareholder quorum subsampling (SQS; quorum level q = 0.69, 1000 iterations). The three diversity measures used are illustrated accordingly: Sampled-in-bin (SIB, blue line), boundary-crosser (BC, red line), and range-through (RT, green line).

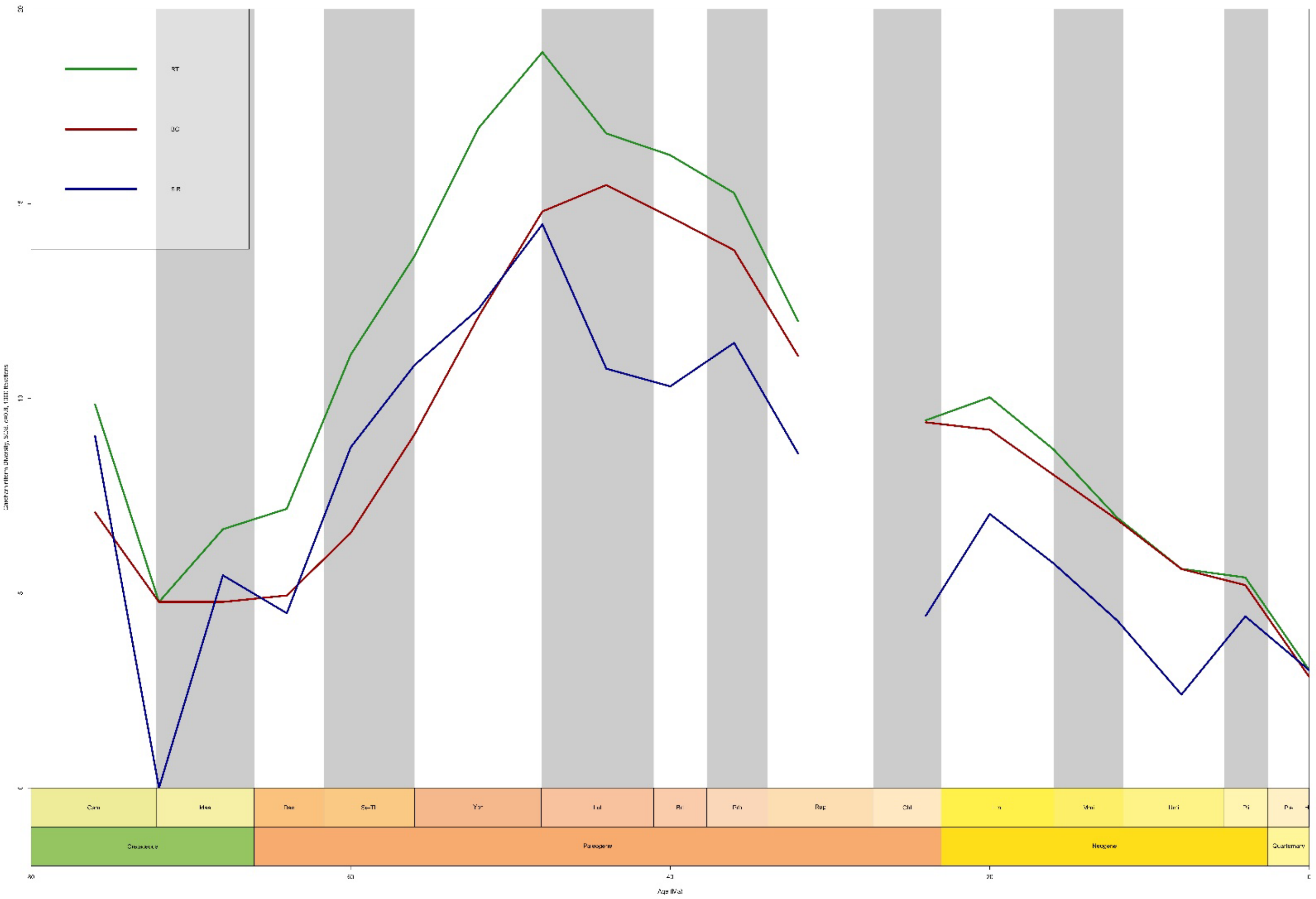

Figure S7: Carcharhiniform genus-level diversity trajectories across the latest Mesozoic and Cenozoic, inferred from shareholder quorum subsampling (SQS; quorum level q = 0.8, 1000 iterations). The three diversity measures used are illustrated accordingly: Sampled-in-bin (SIB, blue line), boundary-crosser (BC, red line), and range-through (RT, green line). The discontinuity is caused by the sample standardisation due to uneven sampling of the taxon during the Oligocene.

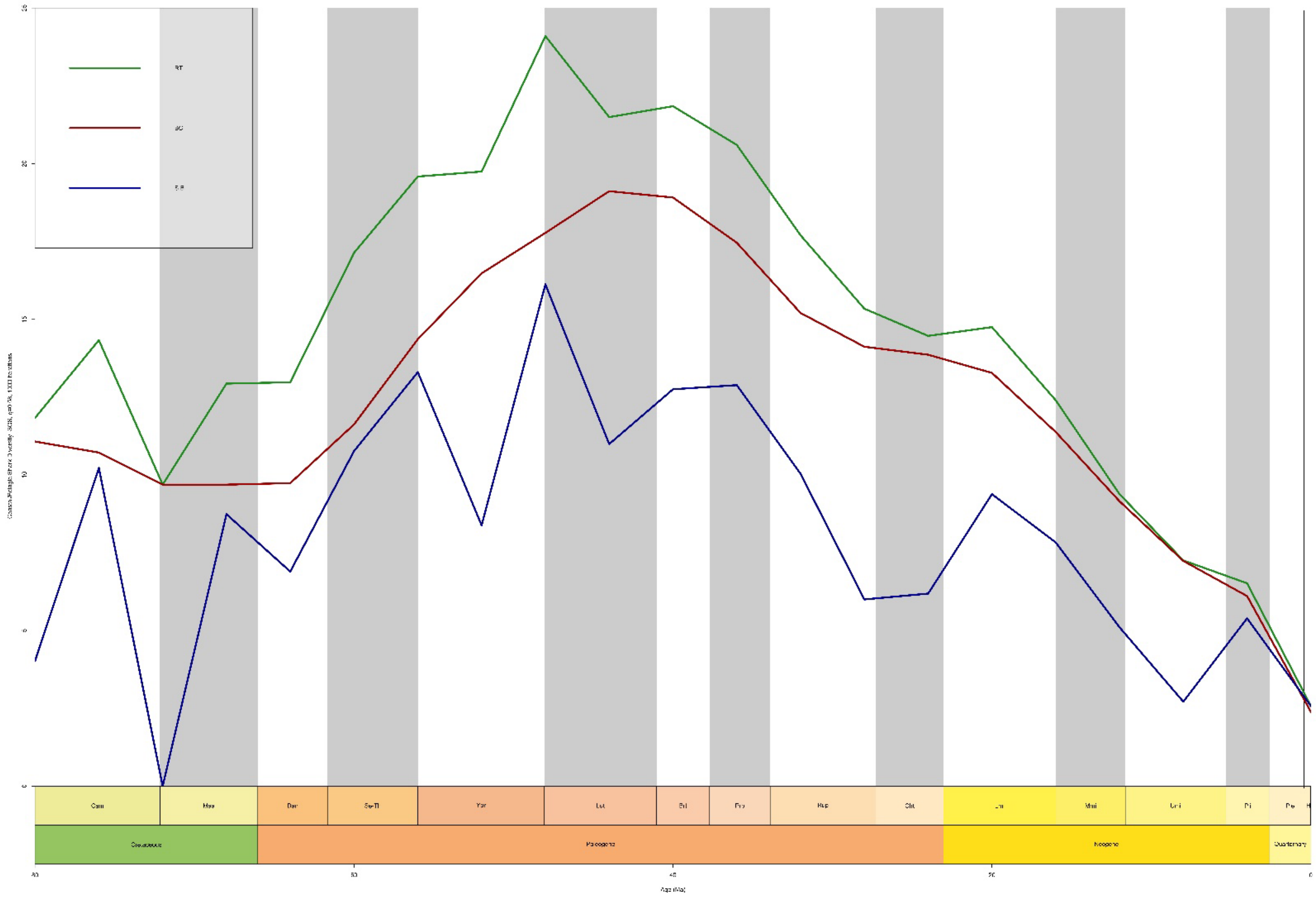

Figure S8: Coastal sharks genus-level diversity trajectories across the latest Mesozoic and Cenozoic, inferred from shareholder quorum subsampling (SQS; quorum level q = 0.58, 1000 iterations). The three diversity measures used are illustrated accordingly: Sampled-in-bin (SIB, blue line), boundary-crosser (BC, red line), and range-through (RT, green line).

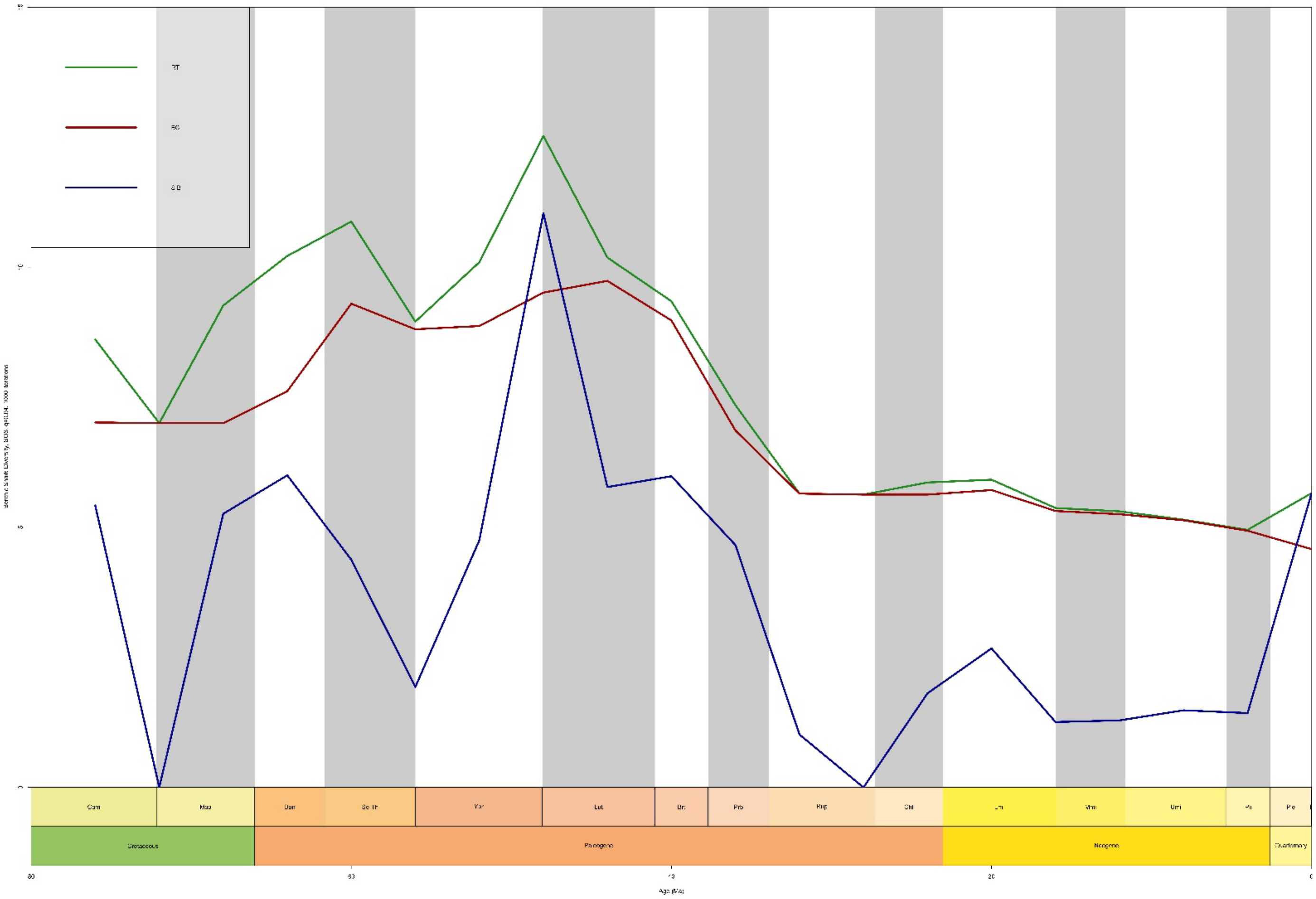

Figure S9: Benthic sharks genus-level diversity trajectories across the latest Mesozoic and Cenozoic, inferred from shareholder quorum subsampling (SQS; quorum level q = 0.64, 1000 iterations). The three diversity measures used are illustrated accordingly: Sampled-in-bin (SIB, blue line), boundary-crosser (BC, red line), and range-through (RT, green line).

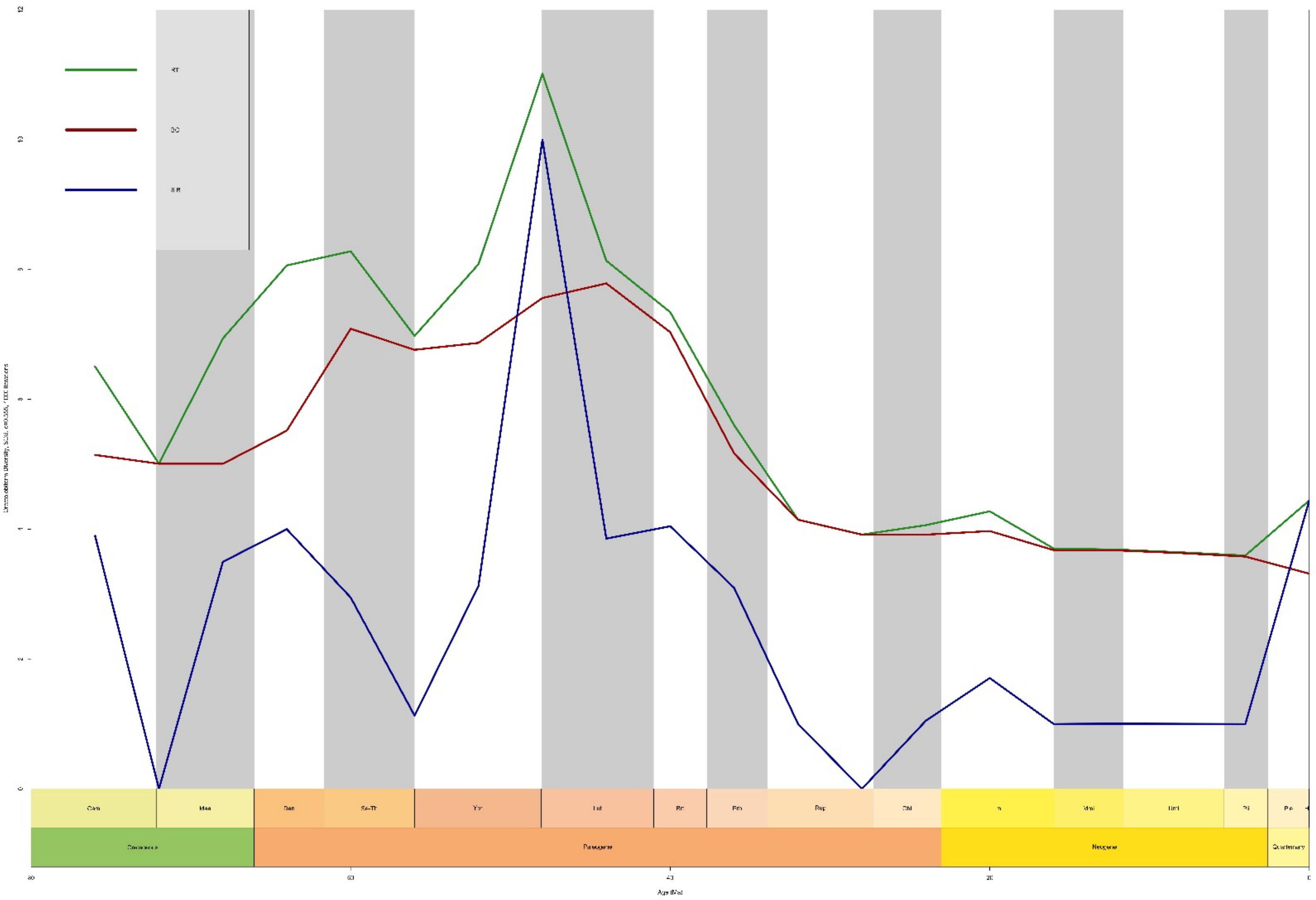

Figure S10: Orectolobiform genus-level diversity trajectories across the latest Mesozoic and Cenozoic, inferred from shareholder quorum subsampling (SQS; quorum level q = 0.55, 1000 iterations). The three diversity measures used are illustrated accordingly: Sampled-in-bin (SIB, blue line), boundary-crosser (BC, red line), and range-through (RT, green line).

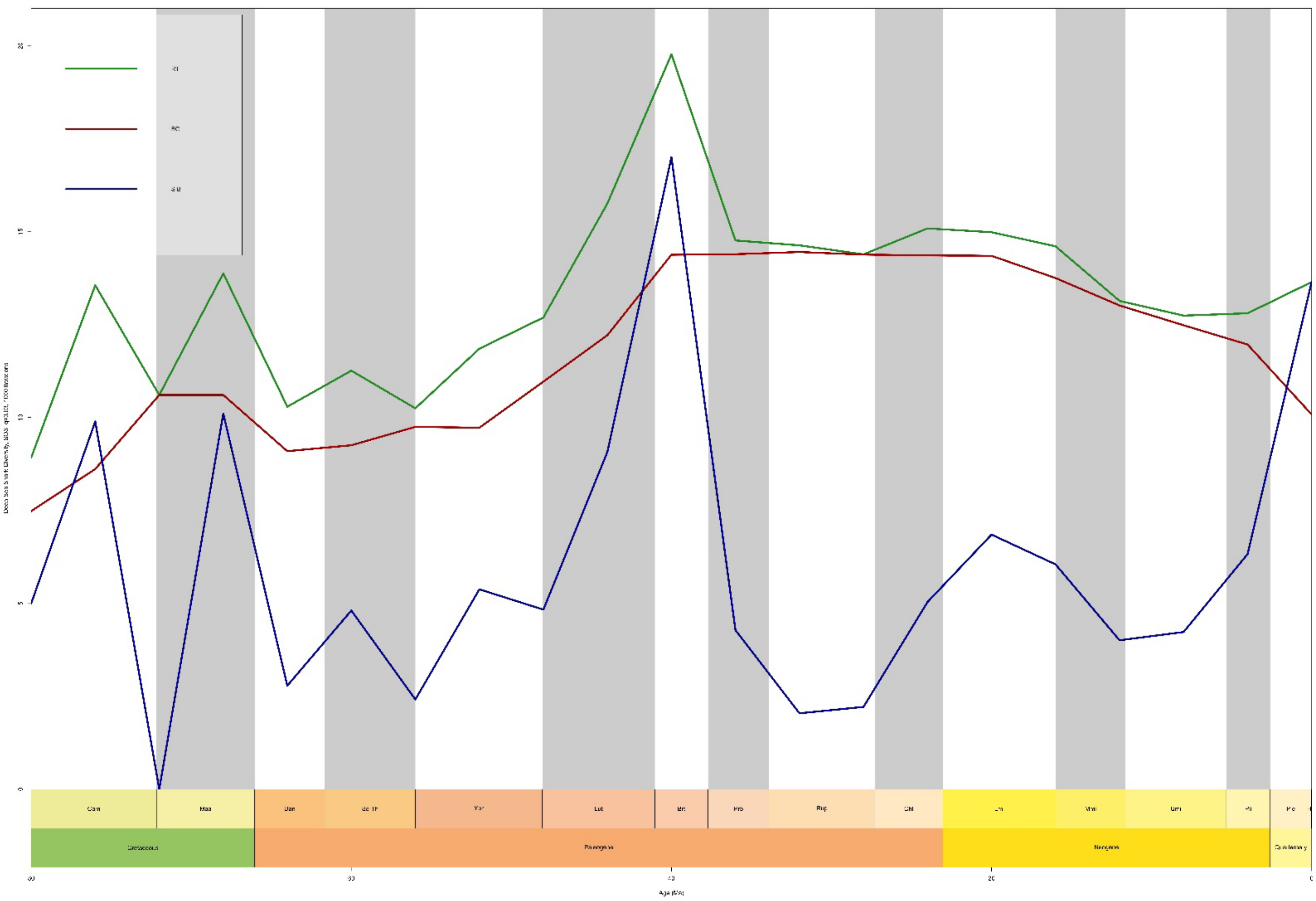

Figure S11: Deep-sea sharks genus-level diversity trajectories across the latest Mesozoic and Cenozoic, inferred from shareholder quorum subsampling (SQS; quorum level q = 0.63, 1000 iterations). The three diversity measures used are illustrated accordingly: Sampled-in-bin (SIB, blue line), boundary-crosser (BC, red line), and range-through (RT, green line).

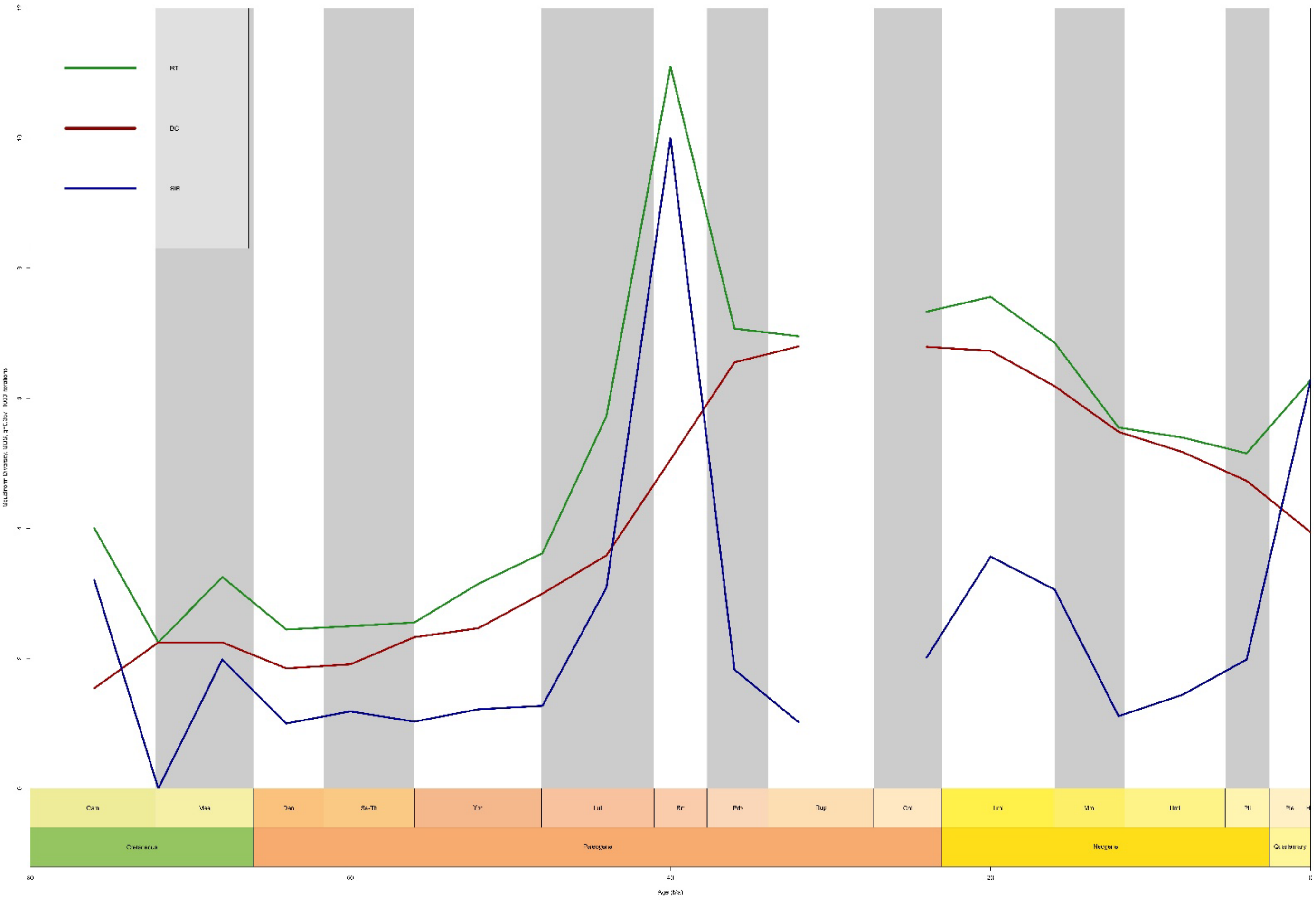

Figure S12: Squaliform genus-level diversity trajectories across the latest Mesozoic and Cenozoic, inferred from shareholder quorum subsampling (SQS; quorum level q = 0.8, 1000 iterations). The three diversity measures used are illustrated accordingly: Sampled-in-bin (SIB, blue line), boundary-crosser (BC, red line), and range-through (RT, green line). The discontinuity is caused by the sample standardisation due to uneven sampling of the taxon during the Oligocene.

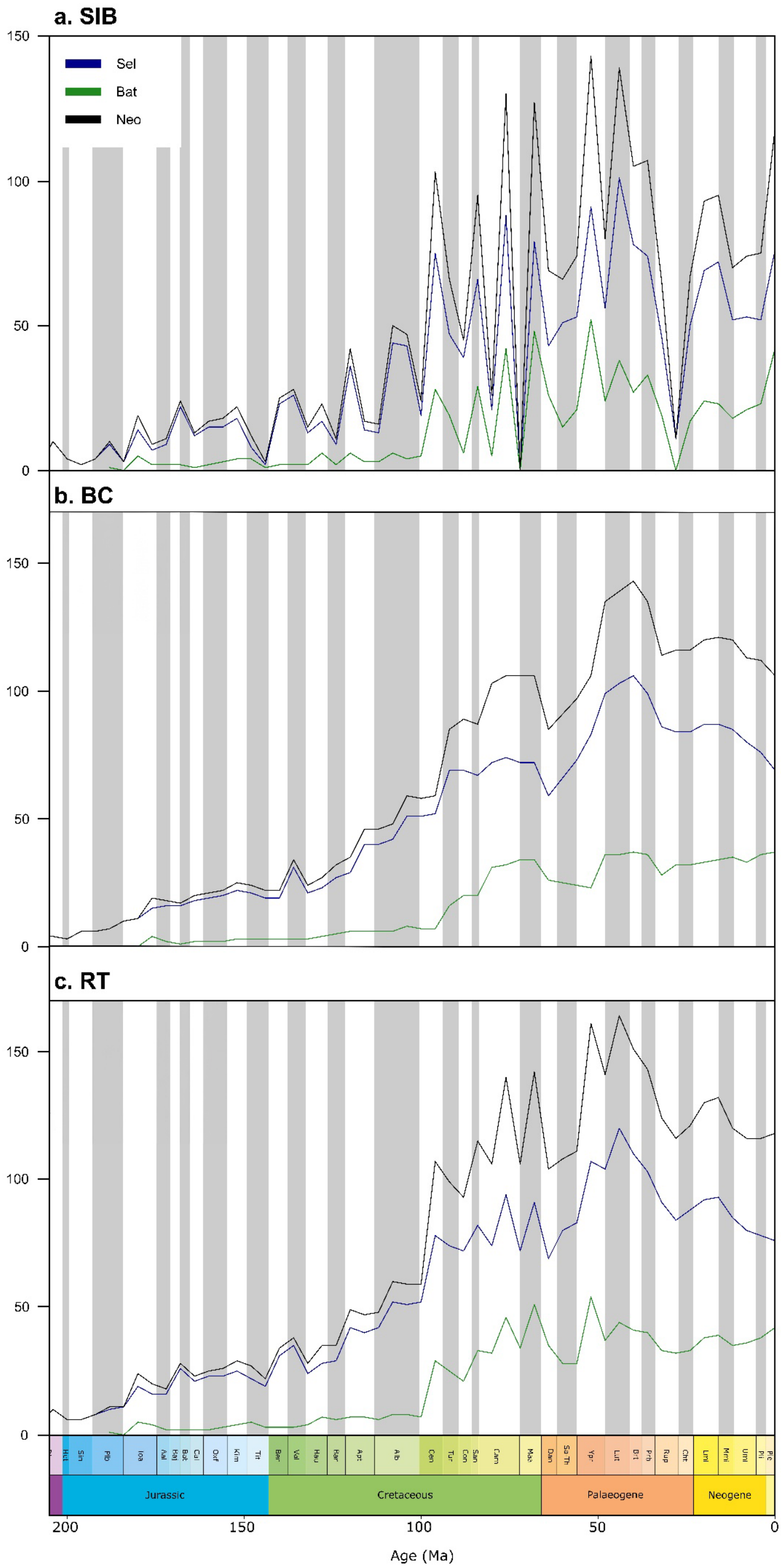

Figure S13: Raw genus-level diversity trajectories of selachians (blue), batoids (green), and neoselachians (black) throughout the Mesozoic and Cenozoic, based on three diversity metrics: (a) sampled-in-bin (SIB), (b) boundary-crosser (BC), and (c) range-through (RT). Diversity estimates are unstandardised and reflect observed taxonomic richness at a given temporal bin.

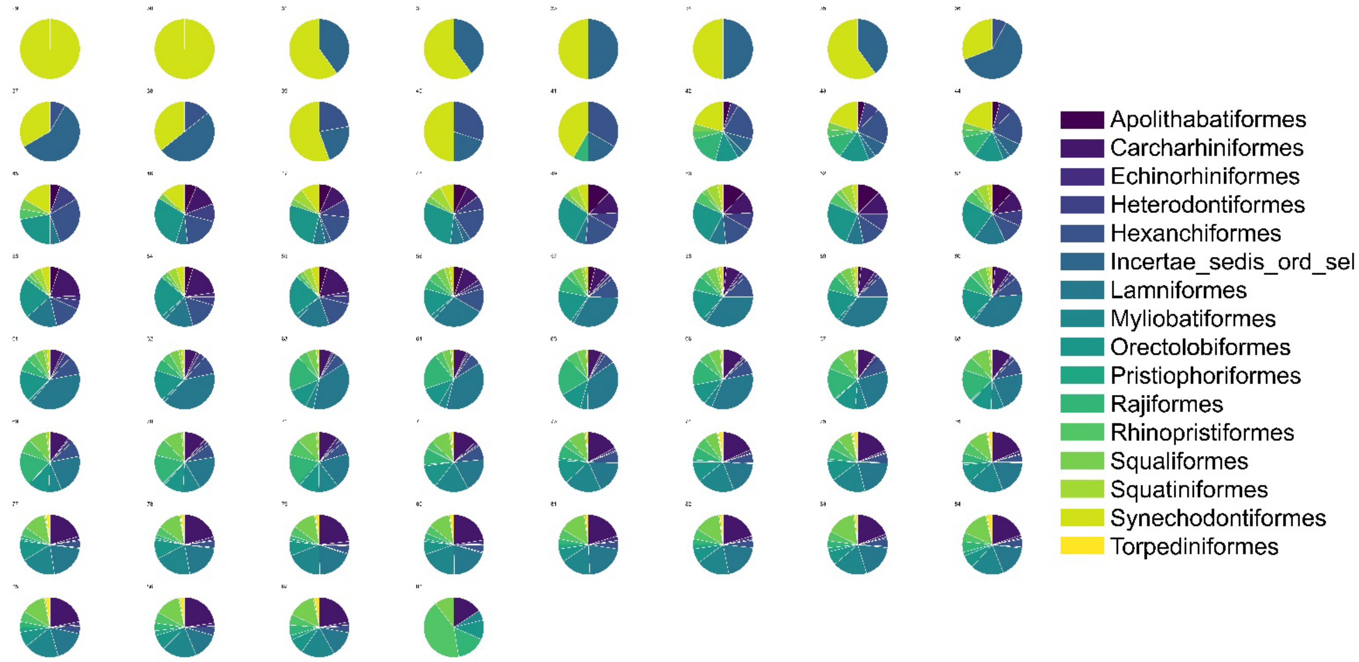

Figure S14: Shifts in the composition of neoselachian taxa over time – illustrated by pie charts representing the proportional richness of genera across individual orders within each time bin. Visualisation commences with bin 29, as earlier intervals are exclusively characterised by Synechodontiformes.

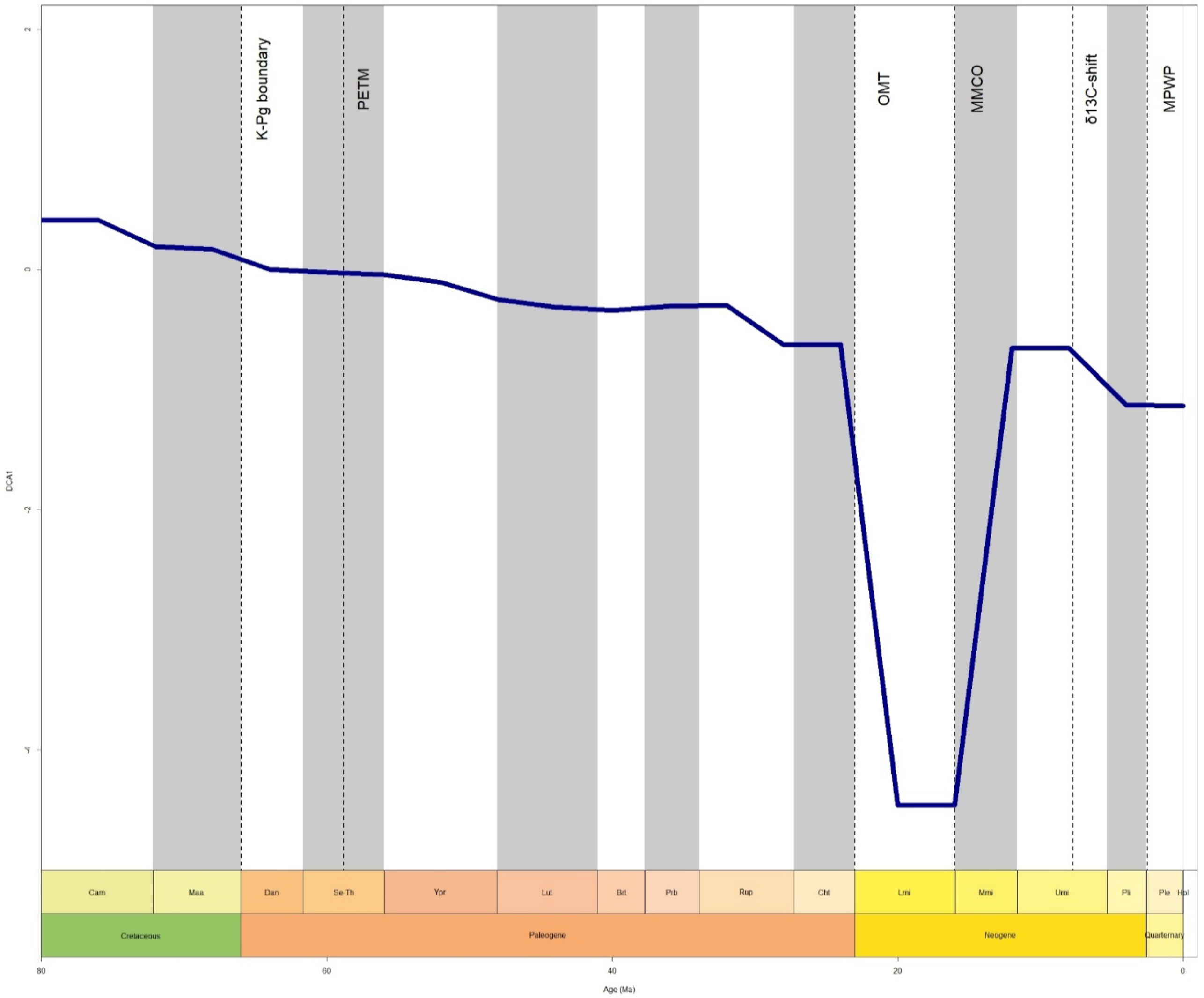

Figure S15: Deep-sea shark faunal composition change (DCA axis 1) throughout the latest Mesozoic and Cenozoic. Blue solid line shows the rate of turnover as a function of its ordination derived from DCA rescaled faunal composition. Y-axis units are standard deviation as a proxy for faunal turnover. Marked events in Earth's history are indicated by dashed lines for reference. The drastic shifts in the Neogene suggest two complete turnovers. Abbreviations: MMCO, mid Miocene climate optimum; MPWP, mid Pliocene warm period; PETM, Palaeocene–Eocene thermal maximum; OMT, Oligocene Miocene climatic transition; δ13C-shift, Carbon ($\delta^{13}C$) isotope shift.

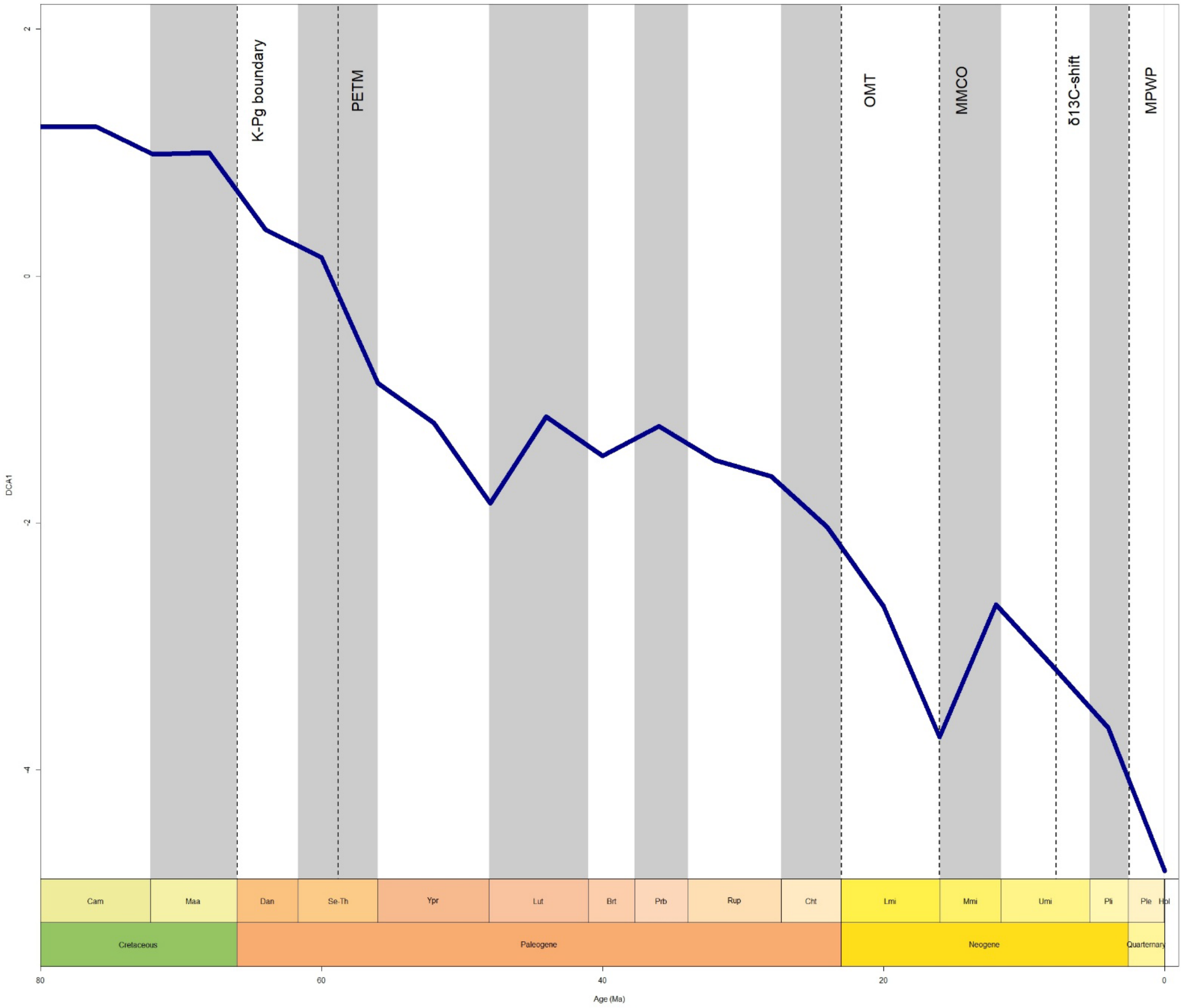

Figure S16: Batoidean faunal composition change (DCA axis 1) throughout the latest Mesozoic and Cenozoic. Blue solid line shows the rate of turnover as a function of its ordination derived from DCA rescaled faunal composition. Y-axis units are standard deviation as a proxy for faunal turnover. Marked events in Earth's history are indicated by dashed lines for reference. Abbreviations: MMCO, mid Miocene climate optimum; MPWP, mid Pliocene warm period; PETM, Palaeocene–Eocene thermal maximum; OMT, Oligocene Miocene climatic transition; δ13C-shift, Carbon ($\delta^{13}C$) isotope shift.

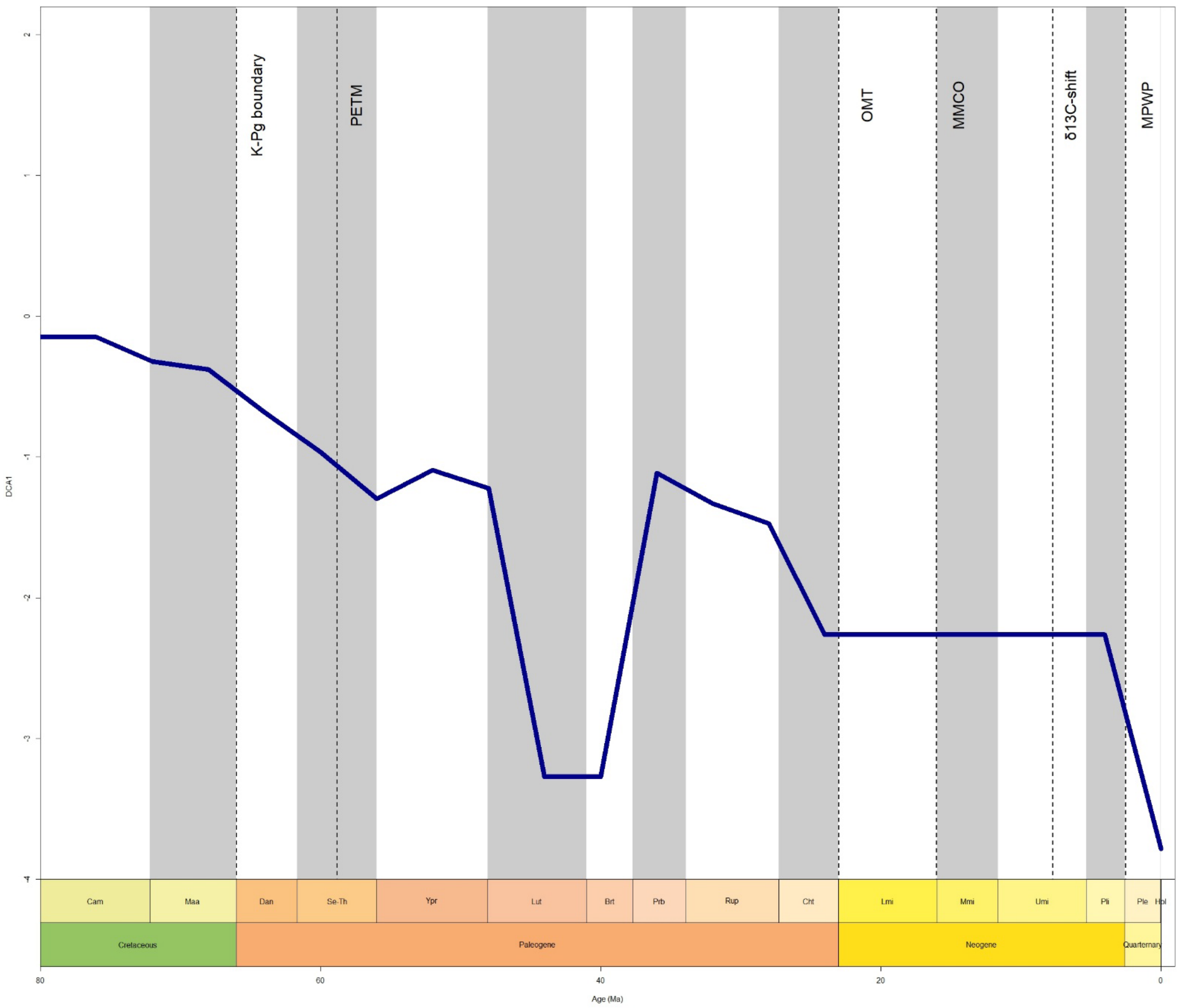

Figure S17: Benthic shark faunal composition change (DCA axis 1) throughout the latest Mesozoic and Cenozoic. Blue solid line shows the rate of turnover as a function of its ordination derived from DCA rescaled faunal composition. Y-axis units are standard deviation as a proxy for faunal turnover. Marked events in Earth's history are indicated by dashed lines for reference. The drastic shifts in the Neogene suggest two complete turnovers. Abbreviations: MMCO, mid Miocene climate optimum; MPWP, mid Pliocene warm period; PETM, Palaeocene–Eocene thermal maximum; OMT, Oligocene Miocene climatic transition; δ13C-shift, Carbon ($\delta^{13}C$) isotope shift.

Supplementary Materials, Tables

Table S1: Neoselachian genera with the respective first (FAD) and last appearance dates (LAD) in million years ago

Order	Genus	FAD	LAD
Carcharhiniformes	*Abdounia*	72.1	27.82
Carcharhiniformes	*Altusmirus*	139.8	132.6
Carcharhiniformes	*Aprionodon*	47.8	5.33
Carcharhiniformes	*Apristurus*	56	0
Carcharhiniformes	*Archaeogaleus*	140.2	132.6
Carcharhiniformes	*Archaeotriakis*	83.5	66
Carcharhiniformes	*Bavariscyllium*	157.3	145
Carcharhiniformes	*Bythaelurus*	33.9	0
Carcharhiniformes	*Cadiera*	140.2	132.6
Carcharhiniformes	*Carcharhinus*	59.2	0
Carcharhiniformes	*Casieria*	66	37.2
Carcharhiniformes	*Cephaloscyllium*	17.277	0
Carcharhiniformes	*Chaenogaleus*	58.7	0
Carcharhiniformes	*Corysodon*	157.3	113
Carcharhiniformes	*Crassescyliorhinus*	85.8	33.9
Carcharhiniformes	*Cretascyliorhinus*	113	86.3
Carcharhiniformes	*Danogaleus*	70.6	61.6
Carcharhiniformes	*Diprosopovenator*	99.6	93.5
Carcharhiniformes	*Eogaleus*	56	47.8
Carcharhiniformes	*Eugaleus*	56	27.82
Carcharhiniformes	*Eypea*	168.3	164.7
Carcharhiniformes	*Florenceodon*	83.5	70.6
Carcharhiniformes	*Fornicatus*	139.8	132.6
Carcharhiniformes	*Foumtizia*	70.6	33.9
Carcharhiniformes	*Galeocerdo*	59.2	0
Carcharhiniformes	*Galeorhinus*	100.5	0
Carcharhiniformes	*Galeus*	55.8	0
Carcharhiniformes	*Glyphis*	23.03	0
Carcharhiniformes	*Hemipristis*	56	0
Carcharhiniformes	*Hemitriakis*	16.4	15.6
Carcharhiniformes	*Hypogaleus*	37.8	0
Carcharhiniformes	*Iago*	41.2	0
Carcharhiniformes	*Isogomphodon*	0.01	0
Carcharhiniformes	*Kallodentis*	56	33.9
Carcharhiniformes	*Khouribgaleus*	61.6	56
Carcharhiniformes	*Leptocharias*	86.3	0
Carcharhiniformes	*Loxodon*	0.01	0
Carcharhiniformes	*Megascyliorhinus*	72.1	2.4
Carcharhiniformes	*Meridiogaleus*	56	33.9
Carcharhiniformes	*Microscyliorhinus*	61.6	33.9
Carcharhiniformes	*Misrichthys*	48.6	33.9
Carcharhiniformes	*Moerigaleus*	41.3	33.9
Carcharhiniformes	*Mustelus*	59.2	0
Carcharhiniformes	*Nasolamia*	0.01	0
Carcharhiniformes	*Negaprion*	56	0
Carcharhiniformes	*Pachygaleus*	59.2	37.8

Carcharhiniformes	*Pachyscyllium*	37.2	3.6
Carcharhiniformes	*Palaeogaleus*	89.8	47.8
Carcharhiniformes	*Palaeoscyllium*	168.3	72.1
Carcharhiniformes	*Palaeotriakis*	86.3	0
Carcharhiniformes	*Paratriakis*	100.5	66
Carcharhiniformes	*Physogaleus*	61.6	1.6
Carcharhiniformes	*Platyrhizodon*	86.3	70.6
Carcharhiniformes	*Platyrhizoscyllium*	47.8	38
Carcharhiniformes	*Porodermoides*	66	56
Carcharhiniformes	*Praeproscyllium*	168.3	166.1
Carcharhiniformes	*Premontreia*	66	4.9
Carcharhiniformes	*Prionace*	23.03	0
Carcharhiniformes	*Prohaploblepharus*	89.8	70.6
Carcharhiniformes	*Protoscyliorhinus*	130	86.3
Carcharhiniformes	*Pseudabdounia*	56	38
Carcharhiniformes	*Pseudoscyliorhinus*	100.5	66
Carcharhiniformes	*Rhaibodus*	38	33.9
Carcharhiniformes	*Rhizoprionodon*	72.1	0
Carcharhiniformes	*Scyliorhinotheca*	38	33.9
Carcharhiniformes	*Scyliorhinus*	139.8	0
Carcharhiniformes	*Sigmoscyllium*	93.9	70.6
Carcharhiniformes	*Sphyrna*	58.7	0
Carcharhiniformes	*Squatigaleus*	83.5	66
Carcharhiniformes	*Stenoscyllium*	56	41.3
Carcharhiniformes	*Thiesus*	167.7	132.6
Carcharhiniformes	*Triaenodon*	47.8	0
Carcharhiniformes	*Triakis*	70.6	0
Carcharhiniformes	*Xystrogaleus*	47.8	38
Echinorhiniformes	*Echinorhinus*	140.2	0
Echinorhiniformes	*Gibbechinorhinus*	72.1	66
Echinorhiniformes	*Orthechinorhinus*	56	28.1
Echinorhiniformes	*Paraechinorhinus*	47.8	5.333
Echinorhiniformes	*Pseudoechinorhinus*	72.1	56
Heterodontiformes	*Cestracion*	113	66
Heterodontiformes	*Heterodontus*	182.7	0
Heterodontiformes	*Paracestracion*	182	132.6
Heterodontiformes	*Procestration*	157.3	145
Heterodontiformes	*Proheterodontus*	168.3	164.7
Heterodontiformes	*Protoheterodontus*	83.6	66
Hexanchiformes	*Chlamydoselachus*	86.3	0
Hexanchiformes	*Crassodontidanus*	190.8	152.1
Hexanchiformes	*Dykeius*	83.5	70.6
Hexanchiformes	*Gladioserratus*	140.2	66
Hexanchiformes	*Heptranchias*	72.1	0
Hexanchiformes	*Hexanchus*	125	0
Hexanchiformes	*Komoksodon*	83.5	70.6
Hexanchiformes	*Macrourogaleus*	157.3	145

Hexanchiformes	*Notidanodon*	136.4	47.8
Hexanchiformes	*Notidanoides*	189.6	145
Hexanchiformes	*Notorynchus*	100.5	0
Hexanchiformes	*Occitanodus*	139.8	132.6
Hexanchiformes	*Pachyhexanchus*	140.2	129.4
Hexanchiformes	*Paraheptranchias*	18.3	17
Hexanchiformes	*Paranotidanus*	168.3	166.1
Hexanchiformes	*Paraorthacodus*	201.3	33.9
Hexanchiformes	*Protoheptranchias*	83.5	70.6
Hexanchiformes	*Pseudonotidanus*	183	157.3
Hexanchiformes	*Rolfodon*	89.8	7.25
Hexanchiformes	*Sphenodus*	208.5	56
Hexanchiformes	*Welcommia*	140.2	132.6
Hexanchiformes	*Weltonia*	66	47.8
Hexanchiformes	*Xampylodon*	125	66
Incertae_sedis_ord_se	*Agaleus*	196.5	174.1
Incertae_sedis_ord_se	*DalatiasIS*	208.5	201.3
Incertae_sedis_ord_se	*Duffinselache*	208.5	201.3
Incertae_sedis_ord_se	*Grozonodon*	228	208.5
Incertae_sedis_ord_se	*Hueneichthys*	208.5	201.3
Incertae_sedis_ord_se	*Microtoxodus*	182.7	174.1
Incertae_sedis_ord_se	*Nanocetorhinus*	20.44	15.155
Incertae_sedis_ord_se	*Odontorhytis*	56	33.9
Incertae_sedis_ord_se	*Ostenoselache*	196.5	189.6
Incertae_sedis_ord_se	*Pseudocetorhinus*	208.5	201.3
Incertae_sedis_ord_se	*Pseudodalatias*	228	201.3
Incertae_sedis_ord_se	*Raineria*	208.5	201.3
Incertae_sedis_ord_se	*Reifia*	221.5	215.56
Incertae_sedis_ord_se	*Squalogaleus*	164.7	161.2
Incertae_sedis_ord_se	*Vallisodus*	208.5	201.3
Lamniformes	*Acrolamna*	100.5	66
Lamniformes	*Acutalamna*	100.5	93.9
Lamniformes	*Alopias*	56	0
Lamniformes	*Anomotodon*	132.9	15.97
Lamniformes	*Aquilolamna*	93.5	89.3
Lamniformes	*Araloselachus*	100.5	0.78
Lamniformes	*Archaeolamna*	125	66
Lamniformes	*Brachycarcharias*	66	33.9
Lamniformes	*Carcharias*	130	0
Lamniformes	*Carcharodon*	66	0
Lamniformes	*Carcharoides*	72.1	5.33
Lamniformes	*Cardabiodon*	100.5	83.6
Lamniformes	*Caucasochasma*	33.9	28.4
Lamniformes	*Cenocarcharias*	100.5	66
Lamniformes	*Cetorhinus*	56	0
Lamniformes	*Cosmopolitodus*	33.9	3.6
Lamniformes	*Cretalamna*	130	11.63

Lamniformes	*Cretodus*	125	66
Lamniformes	*Cretomanta*	100.5	66
Lamniformes	*Cretoxyrhina*	152.1	66
Lamniformes	*Dallasiella*	100.5	89.3
Lamniformes	*Dwardius*	125	83.5
Lamniformes	*Eoptolamna*	130	70.6
Lamniformes	*Eostriatolamia*	113	66
Lamniformes	*Galeocorax*	100.5	66
Lamniformes	*Glueckmanotodus*	59.2	47.8
Lamniformes	*Haimirichia*	100.5	93.5
Lamniformes	*Hispidaspis*	125	83.6
Lamniformes	*Hypotodus*	83.5	11.62
Lamniformes	*Isurolamna*	61.6	17
Lamniformes	*Isurus*	113	0
Lamniformes	*Jaekelotodus*	72.1	33.9
Lamniformes	*Johnlongia*	125.45	83.5
Lamniformes	*Keasius*	52.5	11.63
Lamniformes	*Kenolamna*	100.5	93.5
Lamniformes	*Lamiostoma*	48.6	37.2
Lamniformes	*Lamna*	145	0
Lamniformes	*Leptostyrax*	125	70.6
Lamniformes	*Lethenia*	33.9	27.82
Lamniformes	*Macrorhizodus*	72.1	27.82
Lamniformes	*Megachasma*	28.1	0
Lamniformes	*Megalolamna*	23.9	15.97
Lamniformes	*Mennerotodus*	47.8	38
Lamniformes	*Microcarcharias*	100.5	86.3
Lamniformes	*Mitsukurina*	55.8	0
Lamniformes	*Nanocorax*	100.5	70.6
Lamniformes	*Odontaspis*	140.2	0
Lamniformes	*Orpodon*	61.6	59.2
Lamniformes	*Otodus*	66	2.59
Lamniformes	*Palaeoanacorax*	100.5	89.3
Lamniformes	*Palaeocarcharias*	168.3	145
Lamniformes	*Palaeocarcharodon*	66	33.9
Lamniformes	*Palaeohypotodus*	83.6	19
Lamniformes	*Paleogenotodus*	56	47.8
Lamniformes	*Paraisurus*	125	66
Lamniformes	*Paranomotodon*	113	66
Lamniformes	*Parotodus*	56	1.6
Lamniformes	*Plicatolamna*	113	66
Lamniformes	*Posadaia*	100.5	89.3
Lamniformes	*Protolamna*	140.2	66
Lamniformes	*Pseudocarcharias*	55.8	0
Lamniformes	*Pseudocorax*	100.5	66
Lamniformes	*Pseudodontaspis*	83.5	66
Lamniformes	*Pseudomegachasma*	105.3	89.3

Lamniformes	*Pseudoscapanorhynchus*	113	86.3
Lamniformes	*Ptychodus*	145	72.1
Lamniformes	*Pueblocarcharias*	85.8	83.5
Lamniformes	*Roulletia*	100.5	93.5
Lamniformes	*Scapanorhynchus*	182.7	11.62
Lamniformes	*Scindocorax*	85.8	83.5
Lamniformes	*Serratolamna*	100.5	33.9
Lamniformes	*Squalicorax*	145	38
Lamniformes	*Striatolamia*	83.6	4.3
Lamniformes	*Sylvestrilamia*	56	41.3
Lamniformes	*Synodontaspis*	56	23.03
Lamniformes	*Telodontaspis*	99.6	89.3
Lamniformes	*Tethylamna*	56	38
Lamniformes	*Trigonotodus*	41.2	37.8
Lamniformes	*Truyolsodontos*	100.5	93.5
Lamniformes	*Woellsteinia*	66	38
Lamniformes	*Xiphodolamia*	59.2	5.33
Myliobatiformes	*Aeotbatus*	23.03	0
Myliobatiformes	*Aetobatus*	58.7	0
Myliobatiformes	*Aetomylaeus*	56	0
Myliobatiformes	*Amamriabatis*	41.3	38
Myliobatiformes	*Apocopodon*	100.5	11.62
Myliobatiformes	*Archaeomanta*	66	33.9
Myliobatiformes	*Arechia*	56	38
Myliobatiformes	*Aturobatis*	56	33.9
Myliobatiformes	*Bathytoshia*	20.44	0
Myliobatiformes	*Burnhamia*	61.6	15.97
Myliobatiformes	*Casierabatis*	56	47.8
Myliobatiformes	*Coupatezia*	83.6	33.9
Myliobatiformes	*Dasyatis*	136.4	0
Myliobatiformes	*Dasyrhombodus*	70.6	66
Myliobatiformes	*Delpitia*	66	56
Myliobatiformes	*Enantiobatis*	99.6	93.5
Myliobatiformes	*Eomobula*	56	41.3
Myliobatiformes	*Eoplinthicus*	41.3	33.9
Myliobatiformes	*Eorhinoptera*	56	47.8
Myliobatiformes	*Fontitrygon*	7.25	0
Myliobatiformes	*Garabatis*	56	33.9
Myliobatiformes	*Glikmania*	48.6	37.2
Myliobatiformes	*Gymnura*	99.6	0
Myliobatiformes	*Heterobatis*	61.6	56
Myliobatiformes	*Heterotorpedo*	70.6	41.3
Myliobatiformes	*Hexatrygon*	47.8	0
Myliobatiformes	*Himantura*	47.8	0
Myliobatiformes	*Hypolophites*	66	56
Myliobatiformes	*Hypolophodon*	86.3	15.97
Myliobatiformes	*Igdabatis*	83.6	66

Myliobatiformes	*Ishaquia*	66	56
Myliobatiformes	*Ixobatis*	70.6	61.6
Myliobatiformes	*Jacquhermania*	59.2	33.9
Myliobatiformes	*Leidybatis*	72.1	33.9
Myliobatiformes	*Lessiniabatis*	56	47.8
Myliobatiformes	*Lophobatis*	56	33.9
Myliobatiformes	*Maculabatis*	47.8	41.2
Myliobatiformes	*Mecotrygon*	41.3	38
Myliobatiformes	*Merabatis*	56	41.3
Myliobatiformes	*Meridiania*	56	38
Myliobatiformes	*Mobula*	47.8	0
Myliobatiformes	*Myliobatis*	83.6	0
Myliobatiformes	*Myliodasyatis*	66	61.6
Myliobatiformes	*Neotrygon*	38	0
Myliobatiformes	*Ouledia*	59.2	33.9
Myliobatiformes	*Palaeodasyatis*	66	61.6
Myliobatiformes	*Paratrygon*	37.2	0
Myliobatiformes	*Pastinachus*	38	0
Myliobatiformes	*Phosphatodon*	72.1	66
Myliobatiformes	*Plesiobatis*	0.01	0
Myliobatiformes	*Plinthicus*	33.9	11.62
Myliobatiformes	*Potamotrygon*	48.6	0
Myliobatiformes	*Potobatis*	66	61.1
Myliobatiformes	*Promyliobatis*	56	47.8
Myliobatiformes	*Prosopodon*	70.6	61.6
Myliobatiformes	*Protohimantura*	23.03	15.97
Myliobatiformes	*Pseudaetobatus*	56	33.9
Myliobatiformes	*Pteromylaeus*	23.03	0
Myliobatiformes	*Pteroplatytrygon*	20.44	0
Myliobatiformes	*Pucabatis*	83.6	61.6
Myliobatiformes	*Rhinoptera*	100.5	0
Myliobatiformes	*Rhombodus*	85.8	56
Myliobatiformes	*Saltirius*	40.4	37.2
Myliobatiformes	*Subathunura*	58.7	47.8
Myliobatiformes	*Sulcidens*	61.6	59.2
Myliobatiformes	*Taeniura*	11.62	0
Myliobatiformes	*Taeniurops*	33.9	0
Myliobatiformes	*Tethytrygon*	56	47.8
Myliobatiformes	*Texabatis*	70.6	66
Myliobatiformes	*Urobatis*	56	0
Myliobatiformes	*Urolophus*	56	0
Myliobatiformes	*Viperecucullus*	66	61.7
Myliobatiformes	*Weissobatis*	33.9	27.82
Orectolobiformes	*Acanthoscyllium*	93.9	83.5
Orectolobiformes	*Adnetoscyllium*	100.5	70.6
Orectolobiformes	*Akaimia*	166.1	157.3
Orectolobiformes	*Almascyllium*	99.6	83.5

Orectolobiformes	*Annea*	182.7	93.5
Orectolobiformes	*Brachaelurus*	83.5	0
Orectolobiformes	*Cantioscyllium*	157.3	66
Orectolobiformes	*Cederstroemia*	125	70.6
Orectolobiformes	*Chiloscyllium*	125	0
Orectolobiformes	*Coelometlaouia*	56	41.2
Orectolobiformes	*Cretorectolobus*	136.4	66
Orectolobiformes	*Delpitoscyllium*	66	56
Orectolobiformes	*Dorsetoscyllium*	168.3	166.1
Orectolobiformes	*Eometlaouia*	61.6	41.3
Orectolobiformes	*Eostegostoma*	85.8	33.9
Orectolobiformes	*Eucrossorhinus*	0.01	0
Orectolobiformes	*Ganntouria*	70.6	61.6
Orectolobiformes	*Garrigascyllium*	140.2	132.6
Orectolobiformes	*Ginglymostoma*	100.5	0
Orectolobiformes	*Gryphodobatis*	23.03	5.33
Orectolobiformes	*Hemiscyllium*	113	0
Orectolobiformes	*Heterophorcynus*	168.3	166.1
Orectolobiformes	*Hologinglymostoma*	61.6	56
Orectolobiformes	*Magistrauia*	140.2	132.6
Orectolobiformes	*Mesiteia*	182.7	47.8
Orectolobiformes	*Nebrius*	72.1	0
Orectolobiformes	*Notoramphoscyllium*	56	41.2
Orectolobiformes	*Orectoloboides*	125	37.8
Orectolobiformes	*Orectolobus*	59.2	0
Orectolobiformes	*Ornatoscyllium*	180.1	161.2
Orectolobiformes	*Palaeobrachaelurus*	182.7	125
Orectolobiformes	*Palaeorectolobus*	157.3	145
Orectolobiformes	*Palaeorhincodon*	61.6	33.9
Orectolobiformes	*Paraginglymostoma*	125	113
Orectolobiformes	*Parahemiscyllium*	140.2	132.6
Orectolobiformes	*Pararhincodon*	113	33.9
Orectolobiformes	*Parascyllium*	0.01	0
Orectolobiformes	*Parasquatina*	93.9	66
Orectolobiformes	*Phorcynis*	163.5	145
Orectolobiformes	*Plicatoscyllium*	85.8	38
Orectolobiformes	*Protoginglymostoma*	56	41.3
Orectolobiformes	*Pseudoginglymostoma*	70.6	0
Orectolobiformes	*Pseudospinax*	166.1	93.5
Orectolobiformes	*Restesia*	83.5	66
Orectolobiformes	*Rhincodon*	28.1	0
Orectolobiformes	*Similiteroscyllium*	166.1	132.6
Orectolobiformes	*Squatiscyllium*	83.5	41.3
Orectolobiformes	*Stegostoma*	47.8	0
Pristiophoriformes	*Ikamauius*	28.1	3
Pristiophoriformes	*Pliotrema*	59.2	0
Pristiophoriformes	*Pristiophorus*	85.8	0

Pristiophoriformes	*Propristiophorus*	72.1	66
Rajiformes	*Actinobatis*	85.8	83.5
Rajiformes	*Agaleorhynchus*	85.8	70.6
Rajiformes	*Amblyraja*	5.333	0
Rajiformes	*Angolabatis*	83.5	66
Rajiformes	*Ankistrorhynchus*	89.8	66
Rajiformes	*Antiquaobatis*	189.6	183
Rajiformes	*Archingeayia*	99.6	93.5
Rajiformes	*Arhynchobatis*	17.277	15.155
Rajiformes	*Asflapristis*	93.5	89.3
Rajiformes	*Ataktobatis*	83.5	66
Rajiformes	*Atlanticopristis*	100.5	93.9
Rajiformes	*Atlantoraja*	33.9	0
Rajiformes	*Baharipristis*	99.6	93.5
Rajiformes	*Bathyraja*	56	0
Rajiformes	*Beringraja*	7.25	0
Rajiformes	*Biropristis*	83.6	66
Rajiformes	*Celtipristis*	130	125
Rajiformes	*Columbusia*	85.8	70.6
Rajiformes	*Cristabatis*	182.7	174.1
Rajiformes	*Cruriraja*	0.01	0
Rajiformes	*Ctenopristis*	85.8	61.6
Rajiformes	*Cyclobatis*	100.5	93.5
Rajiformes	*Dalpiazia*	83.5	66
Rajiformes	*Dipturus*	33.9	0
Rajiformes	*Doliobatis*	182.7	174.1
Rajiformes	*Engolismaia*	100.5	93.9
Rajiformes	*Eoplatyrhina*	56	47.8
Rajiformes	*Erguitaia*	85.8	66
Rajiformes	*Ganopristis*	85.8	66
Rajiformes	*Hamrabatis*	100.5	66
Rajiformes	*Hypsobatis*	83.5	66
Rajiformes	*Iansan*	122.46	112.03
Rajiformes	*Ischyrhiza*	100.5	56
Rajiformes	*Kiestus*	99.6	89.3
Rajiformes	*Libanopristis*	100.5	93.5
Rajiformes	*Mafdetia*	99.6	93.5
Rajiformes	*Marambioraja*	56	47.8
Rajiformes	*Marckgrafia*	113	93.5
Rajiformes	*Mesetaraja*	56	47.8
Rajiformes	*Micropristis*	99.6	89.3
Rajiformes	*Nebriimimus*	5.33	3.6
Rajiformes	*Onchopristis*	129.4	33.9
Rajiformes	*Onchosaurus*	100.5	47.8
Rajiformes	*Ostarriraja*	20.44	15.97
Rajiformes	*Parapalaeobates*	93.5	66
Rajiformes	*Pararaja*	99.6	93.5

Rajiformes	*Plicatopristis*	83.5	66
Rajiformes	*Protoplatyrhina*	93.5	66
Rajiformes	*Pseudoraja*	17.277	15.155
Rajiformes	*Ptychotrygon*	100.5	48.6
Rajiformes	*Ptychotrygonoides*	100.5	89.3
Rajiformes	*Pucapristis*	72.1	47.8
Rajiformes	*Raja*	85.8	0
Rajiformes	*Renpetia*	99.6	93.5
Rajiformes	*Rhombopterygia*	99.6	93.5
Rajiformes	*Rostroraja*	0.01	0
Rajiformes	*Schizorhiza*	83.6	56
Rajiformes	*Sclerorhynchus*	100.5	56
Rajiformes	*Smithraja*	56	47.8
Rajiformes	*Springeria*	17.277	15.155
Rajiformes	*Squatirhina*	125.45	66
Rajiformes	*Sympterygia*	0.01	0
Rajiformes	*Tanoutia*	70.6	66
Rajiformes	*Texatrygon*	93.9	70.6
Rajiformes	*Toarcibatis*	182.7	174.1
Rajiformes	*Tomewingia*	72.1	66
Rajiformes	*Turoniabatis*	125	86.3
Rajiformes	*Walteraja*	70.6	66
Rajiformes	*Youssoubatis*	83.5	61.6
Rhinopristiformes	*Aktaua*	48.6	37.2
Rhinopristiformes	*Anoxypristis*	83.5	0
Rhinopristiformes	*Asterodermus*	157.3	145
Rhinopristiformes	*Atlantobatis*	72.1	66
Rhinopristiformes	*Belemnobatis*	182.7	113
Rhinopristiformes	*Borodinopristis*	85.8	70.6
Rhinopristiformes	*Britobatos*	93.9	83.5
Rhinopristiformes	*Cretaplatyrhinoidis*	85.8	70.6
Rhinopristiformes	*Cristomylus*	99.6	66
Rhinopristiformes	*Engaibatis*	152.1	145
Rhinopristiformes	*Eorhinobatos*	56	47.8
Rhinopristiformes	*Glaucopristis*	56	47.8
Rhinopristiformes	*Jurobatos*	182.7	170.3
Rhinopristiformes	*Kimmerobatis*	157.3	145
Rhinopristiformes	*Microbatis*	72.1	66
Rhinopristiformes	*Myledaphus*	93.5	61.6
Rhinopristiformes	*Paratrygonorrhina*	70.6	66
Rhinopristiformes	*Peyeria*	100.5	93.5
Rhinopristiformes	*Platyrhina*	56	0
Rhinopristiformes	*Platyrhinoidis*	47.8	0
Rhinopristiformes	*Plesiozanobatus*	56	47.8
Rhinopristiformes	*Pristis*	66	0
Rhinopristiformes	*Propristis*	55.8	33.9
Rhinopristiformes	*Pseudobatos*	59.2	56

Rhinopristiformes	*Pseudohypolophus*	130	66
Rhinopristiformes	*Pseudomyledaphus*	93.5	66
Rhinopristiformes	*Pseudoplatyrhina*	85.8	70.6
Rhinopristiformes	*Pseudorhinobatos*	56	47.8
Rhinopristiformes	*Rhina*	0.01	0
Rhinopristiformes	*Rhinobatos*	152.1	0
Rhinopristiformes	*Rhynchobatus*	85.8	0
Rhinopristiformes	*Sowibatos*	72.1	66
Rhinopristiformes	*Spathobatis*	168.3	125
Rhinopristiformes	*Terangabatis*	72.1	66
Rhinopristiformes	*Tethybatis*	83.5	66
Rhinopristiformes	*Tingitanius*	100.5	89.3
Rhinopristiformes	*Tlalocbatos*	113	99.6
Rhinopristiformes	*Vascobatis*	70.6	66
Rhinopristiformes	*Zapteryx*	28.1	0
Squaliformes	*Acrosqualiolus*	47.8	37.8
Squaliformes	*Angoumeius*	47.8	38
Squaliformes	*Centrodeania*	66	61.6
Squaliformes	*Centrophoroides*	86.3	66
Squaliformes	*Centrophorus*	83.5	0
Squaliformes	*Centroscymnus*	83.5	0
Squaliformes	*Centroselachus*	66	0
Squaliformes	*Centrosqualus*	85.8	0
Squaliformes	*Cretascymnus*	85.8	66
Squaliformes	*Dalatias*	61.6	0
Squaliformes	*Deania*	83.5	0
Squaliformes	*Dracipinna*	18.3	17
Squaliformes	*Eodalatias*	56	47.8
Squaliformes	*Eoetmopterus*	99.6	66
Squaliformes	*Eoscymnus*	47.8	41.2
Squaliformes	*Eosqualiolus*	47.8	15.97
Squaliformes	*Etmopterus*	48.6	0
Squaliformes	*Euprotomicroides*	0.01	0
Squaliformes	*Fredipristis*	70.6	66
Squaliformes	*Hessinodon*	83.5	70.6
Squaliformes	*Incognitorapax*	66	61.6
Squaliformes	*Isistius*	70.6	0
Squaliformes	*Megasqualus*	66	2.59
Squaliformes	*Microetmopterus*	70.6	66
Squaliformes	*Miroscyllium*	20.44	15.97
Squaliformes	*Mollisquama*	0.01	0
Squaliformes	*Oligodalatias*	33.9	28.1
Squaliformes	*Oxynotus*	5.33	0
Squaliformes	*Palaeocentroscymnus*	20.44	15.155
Squaliformes	*Paraetmopterus*	56	37.8
Squaliformes	*Proetmopterus*	70.6	66
Squaliformes	*Protocentrophorus*	100.5	70.6

Squaliformes	*Protospinax*	182.7	100.5
Squaliformes	*Protosqualus*	130	66
Squaliformes	*Protoxynotus*	93.5	66
Squaliformes	*Rhinoscymnus*	83.5	0
Squaliformes	*Scymnodalatias*	47.8	0
Squaliformes	*Scymnodon*	23.03	0
Squaliformes	*Somniosus*	41.2	0
Squaliformes	*Squaliodalatias*	100.5	15.97
Squaliformes	*Squaliolus*	47.8	0
Squaliformes	*Squalus*	125	0
Squaliformes	*Trigonognathus*	47.8	0
Squaliformes	*Zameus*	5.33	0
Squatiniformes	*Cretasquatina*	83.6	66
Squatiniformes	*Pseudorhina*	163.5	132.6
Squatiniformes	*Squatina*	163.5	0
Synechodontiformes	*Antrigoulia*	140.2	132.6
Synechodontiformes	*Breviacanthus*	168.3	164.7
Synechodontiformes	*Keichouodus*	235	232
Synechodontiformes	*Mucrovenator*	247.2	242
Synechodontiformes	*Nemacanthus*	268.8	166.1
Synechodontiformes	*Palaeospinax*	208.5	157.3
Synechodontiformes	*Palidiplospinax*	201.3	174.1
Synechodontiformes	*Rhomphaiodon*	228	174.1
Synechodontiformes	*Synechodus*	295.5	41.2
Torpediniformes	*Benthobatis*	17.277	0
Torpediniformes	*Eotorpedo*	66	41.3
Torpediniformes	*Narcine*	56	0
Torpediniformes	*Narke*	16.4	15.6
Torpediniformes	*Pachygymnura*	37.8	33.9
Torpediniformes	*Titanonarke*	56	47.8
Torpediniformes	*Torpedo*	56	0

Table S2: Time bins of four million years (Ma) with their respective upper and lower boundary, mid Ma, and bin number.

bottom Ma	mid Ma	top Ma	# bin
350	348	346	1
346	344	342	2
342	340	338	3
338	336	334	4
334	332	330	5
330	328	326	6
326	324	322	7
322	320	318	8
318	316	314	9
314	312	310	10
310	308	306	11
306	304	302	12
302	300	298	13
298	296	294	14
294	292	290	15
290	288	286	16
286	284	282	17
282	280	278	18
278	276	274	19
274	272	270	20
270	268	266	21
266	264	262	22
262	260	258	23
258	256	254	24
254	252	250	25
250	248	246	26
246	244	242	27
242	240	238	28
238	236	234	29
234	232	230	30
230	228	226	31
226	224	222	32
222	220	218	33
218	216	214	34
214	212	210	35
210	208	206	36
206	204	202	37
202	200	198	38
198	196	194	39
194	192	190	40
190	188	186	41
186	184	182	42
182	180	178	43
178	176	174	44
174	172	170	45
170	168	166	46

166	164	162	47
162	160	158	48
158	156	154	49
154	152	150	50
150	148	146	51
146	144	142	52
142	140	138	53
138	136	134	54
134	132	130	55
130	128	126	56
126	124	122	57
122	120	118	58
118	116	114	59
114	112	110	60
110	108	106	61
106	104	102	62
102	100	98	63
98	96	94	64
94	92	90	65
90	88	86	66
86	84	82	67
82	80	78	68
78	76	74	69
74	72	70	70
70	68	66	71
66	64	62	72
62	60	58	73
58	56	54	74
54	52	50	75
50	48	46	76
46	44	42	77
42	40	38	78
38	36	34	79
34	32	30	80
30	28	26	81
26	24	22	82
22	20	18	83
18	16	14	84
14	12	10	85
10	8	6	86
6	4	2	87
2	0	-2	88

Table S3: Sample standardized diversity metrics based on SQS, and environmental data used for the determination of possible extrinsic diversification drivers.

Bin #	mid_MA	sampled	ranged	boundary	SelSIB	Srange	Sboundary	BatSIB	Brange	Bboundary	DCA	SL	SST	DST	CO2	Flood	FragInd	bony	Dino
72	64	28,566	46,73	31,766	15,125	31,664	25,062	17,832	20,84	9,016	-0,308207	61,3	16,68333	18,91133	825,9822	75,688	0,535821	42,44762	166,6618
73	60	31,418	52,197	37,616	23,806	38,932	28,238	7,693	14,518	11,389	-0,4366	26	17,15	16,14982	476,377	74,08745	0,531692	42,71429	152,3786
74	56	30,353	50,78	41,545	21,341	37,396	31,089	10,451	14,514	11,587	-0,793165	2,9	20,06	18,23524	886,474	71,71769	0,536112	142,7193	214,2343
75	52	30,111	55,067	43,604	19,313	39,585	32,646	11,473	16,608	11,402	-0,938802	45,2	22,07	17,55459	1347,091	69,34793	0,527553	382,0175	240,5517
76	48	49,358	66,733	47,771	33,967	47,612	35,5	14,729	19,061	12,885	-1,241705	51,6	21,81	16,18668	1137,59	68,92886	0,523754	315,2703	226,4228
77	44	30,559	58,003	52,219	21,067	41,868	37,824	9,443	16,629	14,836	-1,317556	36,7	15,74	13,91167	999,2107	70,46047	0,522097	238,107	215,856
78	40	36,415	58,727	50,745	26,022	42,395	36,872	10,765	17,184	14,379	-1,418931	56,4	17,31	13,4867	900,2363	71,99208	0,518785	142,9633	213,0554
79	36	33,624	52,289	45,795	21,998	36,744	32,897	11,649	16,622	13,787	-1,777347	35,5	19,19	9,263883	729,6903	69,63156	0,511398	113,9082	210,281
80	32	21,141	40,829	36,609	15,168	29,901	27,309	7,391	13,596	10,934	-1,921987	-1,3	21,8	7,889974	486,182	67,27104	0,500564	189,1769	180,81
81	28	10	35,218	34,305	10	26,413	25,332	0	11,37	11,37	-2,214379	-16,9	14,47	9,20331	433,3218	65,84882	0,511152	211,4738	157,6472
82	24	11,143	33,452	32,639	9,463	24,171	23,5	1,1	11,432	11,37	-2,354098	18,3	18,49	8,04282	546,19	65,36489	0,499343	150,3699	137,9553
83	20	23,862	35,457	31,6	16,298	24,586	22,28	7,874	13,168	11,389	-2,652271	1,2	16,64	8,672515	352,7372	64,88097	0,4999	170,6511	131,7017
84	16	17,498	28,196	25,893	13,334	19,967	18,163	5,589	11,932	11,089	-3,038526	22,4	17,64	7,035324	598,1521	64,54111	0,504454	205,5378	114,4985
85	12	10,558	20,6	20,223	7,107	13,904	13,647	4,976	10,684	10,466	-2,611406	1,4	16,57	8,42503	233,351	64,20125	0,491022	272,7779	101,8398
86	8	4,955	16,083	16,048	3,148	11,006	10,988	2,6	9,242	9,202	-3,443792	-18,9	16,11	6,188954	246,5548	63,52233	0,457428	260,6017	91,10486
87	4	11,915	15,474	14,342	9,034	10,939	10,159	2,711	8,982	8,512	-3,998808	5,4	17,47	3,7579	331	62,50436	0,450636	218,1598	59,89213
88	0	8,582	8,582	7,292	4,734	4,734	4,28	12,516	12,516	7,999	-4,950377	0	18,22	0,7	419	61,48639	0,427122	88	70,25761

Bin #	Nanno	Foram	DeepSIB	DeepRT	DeepBC	CoastSIB	CoastRT	CoastBC	BenthSIB	BenthRT	BenthBC	LamSIB	LamRT	LamBC	CarSIB	CarRT	CarBC
72	19	74,56505	2,769	10,251	9,057	6,901	13,012	9,845	6	10,083	7,482	6,012	10,639	8,703	4,488	7,168	4,944
73	26,5	64,19755	4,827	11,235	9,203	10,799	17,171	11,691	3,946	10,524	9,141	7,005	12,278	9,667	8,747	11,124	6,548
74	42	96,71958	2,416	10,275	9,764	13,202	19,634	14,405	1,987	8,834	8,655	9,111	13,947	11,426	10,859	13,66	9,082
75	37,5	105,414	5,489	11,907	9,724	8,382	19,818	16,47	4,59	9,947	8,744	5,378	13,955	12,331	12,3	16,942	12,101
76	56,5	87,27876	4,822	12,662	11,008	16,167	24,163	17,827	11,041	12,483	9,36	9,159	15,068	12,764	14,473	18,893	14,803
77	68	94,17801	9,091	15,74	12,222	11,173	21,638	19,176	5,683	10,127	9,609	6,567	14,018	13,25	10,766	16,805	15,482
78	60,5	75,2522	17	19,715	14,328	12,814	21,841	18,954	5,966	9,252	8,844	9,826	14,807	13,033	10,312	16,248	14,662
79	55,5	71,27889	4,212	14,669	14,298	12,933	20,618	17,423	4,533	7,271	6,818	6,675	12,09	11,248	11,428	15,276	13,809
80	26,5	52,64891	1,995	14,568	14,386	9,932	17,625	15,147	1,024	5,59	5,59	6,514	10,741	9,197	8,59	11,99	11,096
81	21	40,7645	2,243	14,345	14,336	6	15,119	13,951	0	5,559	5,559	4	9,541	9,057	5,752	8,704	8,383
82	23,5	42,19142	5,004	15,058	14,318	6,107	14,262	13,677	1,805	5,787	5,559	6,47	10,113	9,159	4,423	9,441	9,389
83	40,5	43,25843	6,726	14,999	14,355	9,285	14,571	13,106	2,641	5,831	5,62	6,087	9,062	8,292	7,035	10,03	9,198
84	55,5	45,2112	6,043	14,65	13,78	7,861	12,376	11,294	1,233	5,274	5,235	4,457	7,532	7,045	5,766	8,692	8,037
85	56	58,80407	3,987	13,174	13,037	5,126	9,338	9,093	1,259	5,217	5,171	3,96	6,166	5,886	4,292	6,934	6,881
86	63	66,85393	4,24	12,749	12,502	2,684	7,199	7,184	1,22	5,063	5,048	1,126	4,551	4,546	2,399	5,626	5,622
87	79	59,61541	6,271	12,809	11,998	5,379	6,527	6,116	1,44	4,917	4,899	3,454	4,471	4,115	4,407	5,405	5,208
88	84	46,80662	13,63	13,63	10,071	2,551	2,551	2,346	5,611	5,611	4,553	2,008	2,008	1,873	3,022	3,022	2,851

Abbreviation	Predictors	Source	Reference
**SL**	Sea Level	Miller et al. 2020	Miller, K. G. *et al.* Cenozoic sea-level and cryospheric evolution from deep-sea
**SST**	Sea Surface Temperature	Song et al 2019	Song, H., Wignall, P. B., Song, H., Dai, X. & Chu, D. Seawater Temperature and Dissolved Oxygen over the Past 500 Million Years. J. Earth Sci. 30, 236–243 (2019).
**DST**	Deap Sea Temperature	Meckler et al. 2022	Meckler, A. N. et al. Cenozoic evolution of deep ocean temperature from clumped isotope thermometry. Science 377, 86–90 (2022).
**CO2**	Atmospheric CO2 Concentration	Hönisch et al. 2023	Hönisch, B. et al. Toward a Cenozoic history of atmospheric CO2. Science 382, eadi5177 (2023).
**Flood**	Flooded Continental Area	Marcilly et al. 2022	Marcilly, C. M., Torsvik, T. H. & Conrad, C. P. Global Phanerozoic sea levels from paleogeographic flooding maps. Gondwana Res. 110, 128–142 (2022).
**FragInd**	Continental Fragmentation	Zaffos et al. 2017	Zaffos, A., Finnegan, S. & Peters, S. E. Plate tectonic regulation of global marine animal diversity. Proc. Natl. Acad. Sci. 114, 5653–5658 (2017).
**Bony**	Bony fish sampled in bin diversity	PBDB, Actinopterygii	https://paleobiodb.org/: Data download
**Dino**	Dinoflagellate diversity	Sucheras-Marx et al. 2019	Suchéras-Marx, B. et al. The colonization of the oceans by calcifying pelagic algae. Biogeosciences 16, 2501–2510 (2019).
**Nanno**	Calcaleous nannoplankton diversity	Lowery et al. 2020	Lowery, C. M., Bown, P. R., Fraass, A. J. & Hull, P. M. Ecological Response of Plankton to Environmental Change: Thresholds for Extinction. Annu. Rev. Earth Planet. Sci. 48, 403–429 (2020).
**Foram**	Planktic foraminiferan diversity	Sucheras-Marx et al. 2019	Suchéras-Marx, B. et al. The colonization of the oceans by calcifying pelagic algae. Biogeosciences 16, 2501–2510 (2019).

Abbreviation	Diversity Approach
**sampled**	Sampled in Bin Neoselachians
**boundary**	Boundary Crossers Neoselachians
**DCA**	Detrended Correspondence Analysis Axis 1 Neoselachians
**ranged**	Range Through Neoselachians
**BatSIB**	Sampled in Bin Batoids
**Bboundary**	Boundary Crossers Batoids
**Brange**	Range Through Batoids
**BenthSIB**	Sampled in Bin Benthic
**BenthBC**	Boundary Crossers Benthic
**BenthRT**	Range Through Benthic
**CarSIB**	Sampled in Bin Carcharhiniform
**CarBC**	Boundary Crossers Carcharhiniform
**CarRT**	Range Through Carcharhiniform
**CoastSIB**	Sampled in Bin Coastal
**CoastBC**	Boundary Crossers Coastal
**CoastRT**	Range Through Coastal
**DeepSIB**	Sampled in Bin Deep Sea
**DeepBC**	Boundary Crossers Deep Sea
**DeepRT**	Range Through Deep Sea
**LamSIB**	Sampled in Bin Lamniformes
**LamBC**	Boundary Crossers Lamniformes
**LamRT**	Range Through Lamniformes
**SelSIB**	Sampled in Bin Selachians
**Sboundary**	Boundary Crossers Selachians
**Srange**	Range Through Selachians

Table S4: Detrended correspondence analysis results for neoselachian, deep-sea sharks, batoidean, and benthic sharks fossil occurrences for the Mesozoic and Cenozoic. Values are included for all four axes respectively. Eigenvalues, additive eigenvalues, decorona values and axis length are additionally included below the individual DCA results. Red shadings indicate higher DCA values, green shadings tendentially lower ones.

Neoselachians			
DCA1	DCA2	DCA3	DCA4
1,04849799972	0,00631222778	0,00000001055	0,29996435846
1,04849799972	0,00631222778	0,00000001055	0,29996435846
1,04849799972	0,00631222778	0,00000001055	0,29996435846
1,04849799972	0,00631222778	0,00000001055	0,29996435846
1,04849799972	0,00631222778	0,00000001055	0,29996435846
1,04849799972	0,00631222778	0,00000001055	0,29996435846
1,04849799972	0,00631222778	0,00000001055	0,29996435846
1,04849799972	0,00631222778	0,00000001055	0,29996435846
1,04849799972	0,00631222778	0,00000001055	0,29996435846
1,04849799972	0,00631222778	0,00000001055	0,29996435846
1,04849799972	0,00631222778	0,00000001055	0,29996435846
1,04849799972	0,00631222778	0,00000001055	0,29996435846
1,04849799972	0,00631222778	0,00000001055	0,29996435846
5,50282685194	1,18871098011	0,00000007002	0,48127618583
5,50282685194	1,18871098011	0,00000007002	0,48127618583
5,50282685194	1,18871098011	0,00000007002	0,48127618583
5,50282685194	1,18871098011	0,00000007002	0,48127618583
5,50282685194	1,18871098011	0,00000007002	0,48127618583
5,50282685194	1,18871098011	0,00000007002	0,48127618583
5,50282685194	1,18871098011	0,00000007002	0,48127618583
7,51164394846	1,18840841256	0,00000005043	0,38576146049
7,51164394846	1,18840841256	0,00000005043	0,38576146049
7,51164394846	1,18840841256	0,00000005043	0,38576146049
7,51164394846	1,18840841256	0,00000005043	0,38576146049
7,51164394846	1,18840841256	0,00000005043	0,38576146049
7,77740387120	6,69305348440	-23,71311016722	0,35006477772
7,77740387120	6,69305348440	-23,71311016722	0,35006477772
7,51164394846	1,18840841256	0,00000005043	0,38576146049
7,77740387135	6,59743839637	23,71311023597	0,35006477772
7,77740387135	6,59743839637	23,71311023597	0,35006477772
8,50661517928	1,17796523471	-0,00000015559	0,44491138620
8,50661517928	1,17796523471	-0,00000015559	0,44491138620
10,75536963875	1,17923584454	-0,00000014770	0,42783448847
10,75536963875	1,17923584454	-0,00000014770	0,42783448847
8,50661517928	1,17796523471	-0,00000015559	0,44491138620
9,81861644101	-1,46532792948	0,00000003971	0,33029156597
9,01284313414	-1,60881775012	0,00000009684	0,31316360122
8,03575261911	-1,64036226662	0,00000007386	0,34879477888
7,30000507611	-1,66270938490	-0,00000001123	0,51120564310
7,24505869723	-1,62307961482	-0,00000004164	0,50244537567
7,20520439279	-1,68002029657	-0,00000002909	0,46546469968
6,09257137444	-1,30460245862	-0,00000002397	0,20550093784
5,76324592296	-1,25163397036	-0,00000000694	0,20134115680
5,76324592296	-1,25163397036	-0,00000000694	0,20134115680
4,79828407410	-0,83973147839	-0,00000000010	0,28635150937
4,74444918677	-1,17401233289	0,00000001520	0,39042020312
4,54218283288	-1,25682376393	0,00000000755	0,35960021527
4,47988692530	-1,20197624452	-0,00000001003	0,34158485426
4,38392179528	-1,21478121825	-0,00000000721	0,52527911171
4,11781034761	-1,16243448307	-0,00000000719	0,52084840119
4,05343540276	-1,16028680489	-0,00000000427	0,52362850620
3,88295983130	-1,12636325281	0,00000000426	0,45224258950
3,35659061166	-0,41509708532	0,00000001615	-1,08946218863
3,27557955561	-0,37382161095	0,00000000313	-0,99068832501
3,24705148128	-0,36612236905	0,00000000350	-1,17306716613
2,92768710754	-0,11729626312	0,00000001049	-0,14416582968
2,77687701099	0,13067274697	0,00000000352	-0,08088296760
2,69787831838	0,23313618507	0,00000000770	-0,07673119633
2,69787831838	0,23313618507	0,00000000770	-0,07673119633
2,58055040635	0,32900816728	-0,00000000996	-0,08098239997
2,47218384563	0,33236625567	0,00000000572	-0,06940741033
2,46649509175	0,34791576724	0,00000000357	-0,09302064888
1,77992930469	0,50631514106	-0,00000003948	-2,61814312907
1,68869454827	0,50809549422	-0,00000006347	-2,62112478632
1,58349956713	0,50646650052	-0,00000004861	-0,51885732523
1,14758386361	0,41611263868	-0,00000001441	0,35708180050
0,39293138054	0,26761360088	-0,00000000825	1,09087216906
0,33703939192	0,23521067300	-0,00000000684	1,16122910536
0,33703939192	0,23521067300	-0,00000000684	1,16122910536
0,10515280391	0,19615294283	-0,00000001876	3,11973334138
0,03513468457	0,17142008782	-0,00000001778	1,68320098897
-0,30820719652	0,00970495337	0,00000000527	0,29584545565
-0,43660040509	-0,02042134207	0,00000000949	0,06966925210
-0,79316471362	-0,04414137963	0,00000002291	-2,07724437089
-0,93880194081	-0,01870067089	0,00000002788	-1,70104391880
-1,24170538120	-0,02260786124	0,00000001945	-1,65340169635
-1,31755620101	-0,00477203855	0,00000001090	-0,36537903327
-1,41893056242	-0,00818406006	0,00000000874	-0,40561975525
-1,77734699524	-0,07945567417	0,00000000794	-0,00639199500
-1,92198689627	-0,07720270596	0,00000000861	0,12496332426
-2,21437868983	-0,05335570328	0,00000000989	0,31250867180
-2,35409816222	-0,04695611014	0,00000000852	0,33432188542
-2,65227060824	-0,04437177109	0,00000000851	0,36517519984
-3,03852624225	-0,04295504358	0,00000000811	0,37667012352
-2,61140581507	-0,04637880260	0,00000000592	0,35365758719
-3,44379218116	-0,03175173265	0,00000000513	0,35960694494
-3,99880750779	-0,03082827957	0,00000000901	0,34836665327
-4,95037733259	-0,02558319535	0,00000000623	0,31908300175

Call:
decorana(veg = sharks1, iweigh = 0, iresc = 6, ira = 0)

Detrended correspondence analysis with 26 segments.
Rescaling of axes with 6 iterations.
Total inertia (scaled Chi-square): 6.3982

	DCA1	DCA2	DCA3	DCA4
Eigenvalues	0,75	0,50	0,33	0,25
Additive Eigenvalues	0,75	0,49	0,33	0,25
Decorana values	0,81	0,37	0,33	0,25
Axis lengths	15,71	8,35	47,43	4,32

Deep Sea Sharks			
DCA1	DCA2	DCA3	DCA4
1,70	-9,41	-5,05	0,25
1,70	-9,41	-5,05	0,25
1,65	-6,27	-4,37	0,58
1,65	-6,27	-4,37	0,58
1,79	-5,79	-3,10	-1,99
2,00	-5,27	-1,87	-2,01
2,57	-1,93	-0,31	-2,04
2,57	-1,93	-0,31	-2,04
2,57	-1,93	-0,31	-2,04
2,57	-1,93	-0,31	-2,04
3,60	-1,46	-0,22	-2,00
2,57	-1,93	-0,31	-2,04
2,57	-1,93	-0,31	-2,04
3,54	-2,20	-0,03	4,07
2,39	-2,63	0,00	5,10
1,95	-2,18	0,07	6,31
1,95	-2,18	0,07	6,31
1,62	6,81	0,47	-0,38
1,58	5,28	0,38	-0,71
1,58	5,28	0,38	-0,71
1,54	3,94	0,32	-0,54
1,48	0,62	0,23	-0,47
1,48	0,62	0,23	-0,47
1,48	0,62	0,23	-0,47
1,48	0,62	0,23	-0,47
1,48	0,62	0,23	-0,47
1,48	0,62	0,23	-0,47
1,42	-0,57	0,25	-0,28
1,35	-0,80	0,20	-0,23
1,34	-0,92	0,23	-0,07
1,23	-0,97	0,29	0,04
0,41	-1,14	0,53	0,31
0,41	-1,14	0,53	0,31
0,41	-1,14	0,53	0,31
0,19	-1,40	1,98	0,05
0,17	-1,17	3,39	-0,36
0,00	-0,36	-0,46	-1,23
-0,02	-0,26	-0,45	-1,12
-0,04	-0,02	-0,80	-0,37
-0,11	0,67	-0,84	-0,28
-0,25	1,02	-2,90	0,54
-0,31	0,70	-1,53	0,85
-0,34	0,71	-1,26	0,80
-0,30	0,50	-0,29	0,78
-0,30	0,38	-0,02	0,83
-0,63	0,62	-0,01	0,85
-0,62	0,45	0,10	0,40
-4,46	0,41	0,11	0,35
-4,46	0,41	0,11	0,35
-0,66	0,53	0,15	0,00
-0,66	0,53	0,15	0,00
-1,13	0,67	0,45	-1,37
-1,14	0,72	1,00	-1,89

Call:
decorana(veg = sharks1, iweigh = 0, iresc = 6, ira = 0)

Detrended correspondence analysis with 26 segments.
Rescaling of axes with 6 iterations.
Total inertia (scaled Chi-square): 3.1293

	DCA1	DCA2	DCA3	DCA4
Eigenvalues	0.4990	0.3414	0.2090	0.1614
Additive Eiger	0.4990	0.3057	0.2092	0.1555
Decorana val	0.7493	0.3055	0.1582	0.1284
Axis lengths	8.0618	8.4160	4.5813	7.4444

Batoids			
DCA1	DCA2	DCA3	DCA4
13,80	-0,09	0,31	0,02
12,34	-0,15	0,26	0,62
10,95	-0,16	0,25	0,79
10,95	-0,16	0,25	0,79
9,12	-0,23	0,15	2,31
8,36	-0,13	0,11	2,53
8,36	-0,13	0,11	2,53
8,36	-0,13	0,11	2,53
8,36	0,02	0,62	-10,48
8,26	0,06	0,88	-8,35
8,26	0,06	0,88	-8,35
8,26	0,06	0,88	-8,35
8,15	-0,07	0,46	3,01
7,98	0,28	-0,08	3,63
7,98	0,28	-0,08	3,63
7,46	-0,43	-0,11	4,14
6,37	-0,70	-0,07	2,72
4,56	-0,74	0,00	2,42
4,56	-0,74	0,00	2,42
4,24	-0,61	0,57	2,71
3,57	-0,59	0,80	3,02
3,57	-0,59	0,80	3,02
2,53	3,43	0,55	-0,56
2,46	3,91	0,48	-0,99
2,29	2,26	-0,32	-2,08
2,08	0,68	-1,43	-3,01
1,21	-1,00	-1,00	-1,41
1,21	-1,02	-0,61	-0,93
1,21	-1,02	-0,61	-0,93
0,99	-3,07	-0,09	-0,43
1,00	-1,68	0,05	-0,36
0,37	0,13	1,90	0,20
0,15	0,33	3,17	0,33
-0,87	1,28	1,29	0,72
-1,19	1,05	-0,57	0,99
-1,84	0,80	-0,97	0,89
-1,14	0,21	-1,42	1,19
-1,46	0,21	-2,89	1,13
-1,22	0,01	-1,47	0,73
-1,49	-0,09	-0,70	0,60
-1,62	-0,32	0,43	-0,07
-2,03	-0,33	0,60	0,00
-2,68	-0,34	0,65	0,04
-3,73	-0,33	0,67	0,16
-2,66	-0,33	0,62	0,03
-3,15	-0,35	0,67	0,10
-3,66	-0,35	0,65	0,13
-4,81	-0,32	0,49	0,12

Call:
decorana(veg = sharks1, iweigh = 0, iresc = 6, ira = 0)

Detrended correspondence analysis with 26 segments.
Rescaling of axes with 6 iterations.
Total inertia (scaled Chi-square): 5.9879

	DCA1	DCA2	DCA3	DCA4
Eigenvalues	0.7823	0.4356	0.2168	0.4325
Additive Eiger	0.7823	0.4355	0.2178	0.3946
Decorana val	0.8841	0.3526	0.1921	0.1470
Axis lengths	17.8823	5.5762	6.0528	12.3167

Benthic Sharks			
DCA1	DCA2	DCA3	DCA4
1,20	0,24	-0,53	3,04
2,31	0,20	0,17	3,16
2,31	0,20	0,17	3,16
2,31	0,20	0,17	3,16
5,43	0,21	0,14	1,26
4,08	0,28	0,17	0,59
3,64	0,29	0,14	0,66
3,36	0,30	-1,68	-0,47
2,57	0,30	-2,64	-0,39
2,57	0,30	-2,64	-0,39
2,57	0,30	-2,64	-0,39
2,10	0,31	4,78	-0,15
1,54	0,31	3,15	-0,26
1,54	0,31	3,15	-0,26
1,10	0,33	-0,39	0,97
0,96	0,36	-1,64	1,59
0,88	0,37	-2,50	1,31
0,88	0,37	-2,50	1,31
0,71	0,34	-1,03	0,13
0,68	0,31	-0,49	-0,33
0,68	0,31	-0,49	-0,33
0,55	0,33	-0,47	-0,84
0,55	0,33	-0,47	-0,84
0,49	0,27	-0,40	-1,83
0,23	0,27	-0,07	-3,61
-0,12	-0,42	0,20	-2,66
-0,15	-0,52	0,29	-0,96
-0,15	-0,52	0,29	-0,96
-0,32	-0,34	0,32	-0,79
-0,38	-0,37	0,40	-0,72
-0,68	-0,46	0,33	1,78
-0,96	-1,34	0,33	1,94
-1,30	-3,34	0,26	1,20
-1,09	-2,33	0,19	0,15
-1,22	-1,65	0,19	0,14
-3,27	-1,74	0,19	-0,08
-3,27	-1,74	0,19	-0,08
-1,11	0,36	0,20	0,63
-1,33	0,41	0,25	0,39
-1,47	0,72	0,28	0,32
-2,26	2,63	0,27	0,28
-2,26	2,63	0,27	0,28
-2,26	2,63	0,27	0,28
-2,26	2,63	0,27	0,28
-2,26	2,63	0,27	0,28
-2,26	2,63	0,27	0,28
-3,79	0,65	0,27	0,16

Call:
decorana(veg = sharks1, iweigh = 0, iresc = 6, ira = 0)

Detrended correspondence analysis with 26 segments.
Rescaling of axes with 6 iterations.
Total inertia (scaled Chi-square): 2.8758

	DCA1	DCA2	DCA3	DCA4
Eigenvalues	0.6269	0.2773	0.2091	0.2327
Additive Eiger	0.6269	0.2724	0.2034	0.2334
Decorana val	0.6958	0.2652	0.1976	0.1418
Axis lengths	9.2139	4.9704	6.0930	4.6972

Table S4: Results of the Spearman's rank correlation analysis per taxonomic or ecologic (sub)sample unit and diversity approach (SQS) for biotic and abiotic predictors. Black font indicates significance ($p < 0.05$). Red shadings indicate high Spearman's ρ (Rho) or low p-value.

Diversity	Predictor	Rho	p (CI =0,95)
BatSIB	Bboundary	0,28711678	0,26383423
BatSIB	BenthBC	0,49908041	0,041401444
BatSIB	BenthRT	0,69362745	0,002014144
BatSIB	BenthSIB	0,8627451	8,31713E-06
BatSIB	bony	-0,304	2,36E-01
BatSIB	Brange	0,83088235	3,61106E-05
BatSIB	CarBC	0,20098039	0,439233876
BatSIB	CarRT	0,41421569	0,098331188
BatSIB	CarSIB	0,51715686	0,033511352
BatSIB	CO2	0,610	9,27E-03
BatSIB	CoastBC	0,30392157	0,235635841
BatSIB	CoastRT	0,41666667	0,09615711
BatSIB	CoastSIB	0,47794118	0,052328542
BatSIB	DeepBC	-0,4877451	0,047021097
BatSIB	DeepRT	-0,245098	0,343046653
BatSIB	DeepSIB	0,17156863	0,51026631
BatSIB	Dino	0,512	3,55E-02
BatSIB	DST	0,544	2,39E-02
BatSIB	Flood	0,480	5,10E-02
BatSIB	Foram	0,613	8,92E-03
BatSIB	FragInd	0,505	3,87E-02
BatSIB	LamBC	0,38480392	0,127215995
BatSIB	LamRT	0,4754902	0,053722731
BatSIB	LamSIB	0,42401961	0,089841685
BatSIB	Nanno	0,119	6,49E-01
BatSIB	SL	0,637	5,93E-03
BatSIB	SST	0,429	8,58E-02
Bboundary	BenthBC	0,82872944	3,9442E-05
Bboundary	BenthRT	0,65766921	0,004112755
Bboundary	BenthSIB	0,41595123	0,096788124
Bboundary	bony	0,088	7,36E-01
Bboundary	CarBC	0,87852827	3,48995E-06
Bboundary	CarRT	0,90797614	4,75159E-07
Bboundary	CarSIB	0,85398837	1,28781E-05
Bboundary	CO2	0,699	1,78E-03
Bboundary	CoastBC	0,91288412	3,19638E-07
Bboundary	CoastRT	0,92024609	1,68483E-07
Bboundary	CoastSIB	0,85030739	1,53488E-05
Bboundary	DeepBC	0,12024549	0,645735005
Bboundary	DeepRT	0,33619657	0,187038973
Bboundary	DeepSIB	0,15582834	0,550357185
Bboundary	Dino	0,800	1,15E-04
Bboundary	DST	0,604	1,03E-02
Bboundary	Flood	0,622	7,67E-03
Bboundary	Foram	0,507	3,79E-02
Bboundary	FragInd	0,605	1,01E-02
Bboundary	LamBC	0,91043013	3,90816E-07
Bboundary	LamRT	0,85766936	1,07536E-05
Bboundary	LamSIB	0,80981656	8,16077E-05
Bboundary	Nanno	-0,019	9,42E-01
Bboundary	SL	0,499	4,13E-02
Bboundary	SST	0,161	5,38E-01
BenthBC	bony	-0,034	8,96E-01
BenthBC	CarBC	0,68546917	0,002388332
BenthBC	CarRT	0,88166785	2,89491E-06
BenthBC	CarSIB	0,84610684	1,86485E-05
BenthBC	CO2	0,791	1,57E-04
BenthBC	Dino	0,871	5,47E-06
BenthBC	DST	0,847	1,76E-05
BenthBC	Flood	0,860	9,77E-06

Abbreviation	Diversity Approach
sampled	Sampled in Bin Neoselachians
boundary	Boundary Crossers Neoselachians
DCA	Detrended Correspondence Analysis Axis 1 Neoselachians
ranged	Range Through Neoselachians
BatSIB	Sampled in Bin Batoids
Bboundary	Boundary Crossers Batoids
Brange	Range Through Batoids
BenthSIB	Sampled in Bin Benthic
BenthBC	Boundary Crossers Benthic
BenthRT	Range Through Benthic
CarSIB	Sampled in Bin Carcharhiniform
CarBC	Boundary Crossers Carcharhiniform
CarRT	Range Through Carcharhiniform
CoastSIB	Sampled in Bin Coastal
CoastBC	Boundary Crossers Coastal
CoastRT	Range Through Coastal
DeepSIB	Sampled in Bin Deep Sea
DeepBC	Boundary Crossers Deep Sea
DeepRT	Range Through Deep Sea
LamSIB	Sampled in Bin Lamniformes
LamBC	Boundary Crossers Lamniformes
LamRT	Range Through Lamniformes
SelSIB	Sampled in Bin Selachians
Sboundary	Boundary Crossers Selachians
Srange	Range Through Selachians

Abbreviation	Predictors
SL	Sea Level
SST	Sea Surface Temperature
DST	Deap Sea Temperature
CO2	Atmospheric CO2 Concentration
Flood	Flooded Continental Area
FragInd	Continental Fragmentation
Bony	Bony fish diversity
Dino	Dinoflagellate diversity
Nanno	Calcaleous nannoplankton diversity
Foram	Planktic foraminiferan diversity

BenthBC	Foram	0,656	**4,24E-03**
BenthBC	FragInd	0,851	**1,48E-05**
BenthBC	LamBC	0,92090759	**1,58591E-07**
BenthBC	LamRT	0,9503374	**5,27382E-09**
BenthBC	LamSIB	0,84242812	**2,20141E-05**
BenthBC	Nanno	-0,212	4,13E-01
BenthBC	SL	0,695	1,94E-03
BenthBC	SST	0,168	5,19E-01
BenthRT	BenthBC	0,91600262	**2,45439E-07**
BenthRT	bony	-0,228	3,79E-01
BenthRT	CarBC	0,49019608	**0,04575998**
BenthRT	CarRT	0,74019608	**0,00067992**
BenthRT	CarSIB	0,73529412	**0,000770053**
BenthRT	CO2	0,767	3,26E-04
BenthRT	Dino	0,767	**3,26E-04**
BenthRT	DST	0,826	4,41E-05
BenthRT	Flood	0,809	8,46E-05
BenthRT	Foram	0,600	**1,08E-02**
BenthRT	FragInd	0,806	**9,24E-05**
BenthRT	LamBC	0,77696078	0,000243315
BenthRT	LamRT	0,83823529	**2,64641E-05**
BenthRT	LamSIB	0,75490196	**0,000460298**
BenthRT	Nanno	-0,213	4,11E-01
BenthRT	SL	0,733	**8,19E-04**
BenthRT	SST	0,250	3,33E-01
BenthSIB	BenthBC	0,62293083	0,007559115
BenthSIB	BenthRT	0,80392157	0,000100739
BenthSIB	bony	-0,223	3,90E-01
BenthSIB	CarBC	0,29901961	0,243655338
BenthSIB	CarRT	0,45833333	0,064271303
BenthSIB	CarSIB	0,47058824	0,056594109
BenthSIB	CO2	0,640	**5,68E-03**
BenthSIB	Dino	0,498	**4,21E-02**
BenthSIB	DST	0,569	**1,72E-02**
BenthSIB	Flood	0,517	**3,35E-02**
BenthSIB	Foram	0,610	9,27E-03
BenthSIB	FragInd	0,478	5,23E-02
BenthSIB	LamBC	0,49264706	**0,044524526**
BenthSIB	LamRT	0,56372549	**0,01843499**
BenthSIB	LamSIB	0,49509804	0,043314417
BenthSIB	Nanno	0,198	4,47E-01
BenthSIB	SL	0,801	**1,10E-04**
BenthSIB	SST	0,230	3,74E-01
boundary	BatSIB	0,39215686	0,119499785
boundary	Bboundary	0,92270008	**1,34251E-07**
boundary	BenthBC	0,90864517	4,50751E-07
boundary	BenthRT	0,75980392	**0,000401789**
boundary	BenthSIB	0,48284314	**0,04962159**
boundary	bony	0,044	8,66E-01
boundary	Brange	0,75980392	**0,000401789**
boundary	CarBC	0,8627451	8,31713E-06
boundary	CarRT	0,94852941	6,8596E-09
boundary	CarSIB	0,8872549	2,04831E-06
boundary	CO2	0,821	5,35E-05
boundary	CoastBC	0,96078431	9,24916E-10
boundary	CoastRT	0,98039216	5,40856E-12
boundary	CoastSIB	0,86519608	**7,32003E-06**
boundary	DeepBC	0,00735294	0,977655893
boundary	DeepRT	0,18872549	0,468192284
boundary	DeepSIB	-0,0122549	0,962767575
boundary	Dino	0,926	**9,33E-08**
boundary	DST	0,713	**1,31E-03**
boundary	Flood	0,777	**2,43E-04**

boundary	Foram	0,608	9,64E-03
boundary	FragInd	0,730	8,70E-04
boundary	LamBC	0,99019608	3,06422E-14
boundary	LamRT	0,96568627	3,44636E-10
boundary	LamSIB	0,85294118	1,35439E-05
boundary	Nanno	-0,145	5,79E-01
boundary	Sboundary	0,99754902	0
boundary	SelSIB	0,87745098	3,71678E-06
boundary	SL	0,603	1,04E-02
boundary	Srange	0,95343137	3,28499E-09
boundary	SST	0,255	3,23E-01
Brange	Bboundary	0,6110434	0,009164685
Brange	BenthBC	0,8448806	1,97182E-05
Brange	BenthRT	0,90196078	7,504E-07
Brange	BenthSIB	0,82352941	4,85755E-05
Brange	bony	-0,279	2,77E-01
Brange	CarBC	0,51960784	0,032537167
Brange	CarRT	0,71813725	0,001167042
Brange	CarSIB	0,73284314	0,000818701
Brange	CO2	0,821	5,35E-05
Brange	CoastBC	0,66421569	0,003636349
Brange	CoastRT	0,75	0,000525731
Brange	CoastSIB	0,74019608	0,00067992
Brange	DeepBC	-0,3088235	0,227785653
Brange	DeepRT	-0,0661765	0,800774094
Brange	DeepSIB	0,06862745	0,793542443
Brange	Dino	0,792	1,53E-04
Brange	DST	0,777	2,43E-04
Brange	Flood	0,824	4,86E-05
Brange	Foram	0,642	5,45E-03
Brange	FragInd	0,775	2,62E-04
Brange	LamBC	0,75245098	0,000492111
Brange	LamRT	0,81862745	5,87601E-05
Brange	LamSIB	0,74264706	0,000638247
Brange	Nanno	-0,161	5,38E-01
Brange	SL	0,797	1,30E-04
Brange	SST	0,299	2,44E-01
CarBC	bony	0,324	2,05E-01
CarBC	CO2	0,676	2,87E-03
CarBC	Dino	0,787	1,79E-04
CarBC	DST	0,395	1,17E-01
CarBC	Flood	0,409	1,03E-01
CarBC	Foram	0,382	1,30E-01
CarBC	FragInd	0,373	1,41E-01
CarBC	Nanno	-0,002	9,93E-01
CarBC	SL	0,404	1,07E-01
CarBC	SST	0,301	2,40E-01
CarRT	bony	0,186	4,74E-01
CarRT	CarBC	0,90441176	6,25179E-07
CarRT	CO2	0,824	4,86E-05
CarRT	Dino	0,934	4,33E-08
CarRT	DST	0,676	2,87E-03
CarRT	Flood	0,657	4,17E-03
CarRT	Foram	0,598	1,12E-02
CarRT	FragInd	0,681	2,60E-03
CarRT	Nanno	-0,142	5,86E-01
CarRT	SL	0,556	2,04E-02
CarRT	SST	0,424	8,98E-02
CarSIB	bony	0,081	7,58E-01
CarSIB	CarBC	0,78431373	0,000193652
CarSIB	CarRT	0,94852941	6,8596E-09
CarSIB	CO2	0,824	4,86E-05
CarSIB	Dino	0,900	8,96E-07

CarSIB	DST	0,708	1,46E-03
CarSIB	Flood	0,667	3,47E-03
CarSIB	Foram	0,620	7,92E-03
CarSIB	FragInd	0,750	5,26E-04
CarSIB	Nanno	-0,142	5,86E-01
CarSIB	SL	0,581	1,45E-02
CarSIB	SST	0,510	3,66E-02
CoastBC	BenthBC	0,80564086	9,48062E-05
CoastBC	BenthRT	0,62254902	0,00760691
CoastBC	BenthSIB	0,37009804	0,143675473
CoastBC	bony	0,159	5,41E-01
CoastBC	CarBC	0,95343137	3,28499E-09
CoastBC	CarRT	0,94607843	9,65394E-09
CoastBC	CarSIB	0,85784314	1,06611E-05
CoastBC	CO2	0,782	2,09E-04
CoastBC	Dino	0,900	8,96E-07
CoastBC	DST	0,574	1,61E-02
CoastBC	Flood	0,625	7,30E-03
CoastBC	Foram	0,483	4,96E-02
CoastBC	FragInd	0,581	1,45E-02
CoastBC	LamBC	0,94852941	6,8596E-09
CoastBC	LamRT	0,8995098	8,96482E-07
CoastBC	LamSIB	0,78921569	0,000165507
CoastBC	Nanno	-0,121	6,42E-01
CoastBC	SL	0,485	4,83E-02
CoastBC	SST	0,277	2,82E-01
CoastRT	BenthBC	0,88779906	1,97855E-06
CoastRT	BenthRT	0,73529412	0,000770053
CoastRT	BenthSIB	0,46568627	0,059578242
CoastRT	bony	0,096	7,15E-01
CoastRT	CarBC	0,89215686	1,48935E-06
CoastRT	CarRT	0,97303922	5,76923E-11
CoastRT	CarSIB	0,92156863	1,49208E-07
CoastRT	CO2	0,797	1,30E-04
CoastRT	CoastBC	0,96813725	1,99099E-10
CoastRT	Dino	0,926	9,33E-08
CoastRT	DST	0,686	2,35E-03
CoastRT	Flood	0,716	1,24E-03
CoastRT	Foram	0,576	1,55E-02
CoastRT	FragInd	0,694	2,01E-03
CoastRT	LamBC	0,96813725	1,99099E-10
CoastRT	LamRT	0,95833333	1,44699E-09
CoastRT	LamSIB	0,86519608	7,32003E-06
CoastRT	Nanno	-0,135	6,06E-01
CoastRT	SL	0,554	2,10E-02
CoastRT	SST	0,324	2,05E-01
CoastSIB	BenthBC	0,8448806	1,97182E-05
CoastSIB	BenthRT	0,71813725	0,001167042
CoastSIB	BenthSIB	0,45588235	0,06589472
CoastSIB	bony	-0,118	6,53E-01
CoastSIB	CarBC	0,74509804	0,000598715
CoastSIB	CarRT	0,87745098	3,71678E-06
CoastSIB	CarSIB	0,91666667	2,31712E-07
CoastSIB	CO2	0,725	9,80E-04
CoastSIB	CoastBC	0,82352941	4,85755E-05
CoastSIB	CoastRT	0,8995098	8,96482E-07
CoastSIB	Dino	0,801	1,10E-04
CoastSIB	DST	0,664	3,64E-03
CoastSIB	Flood	0,721	1,10E-03
CoastSIB	Foram	0,556	2,04E-02
CoastSIB	FragInd	0,728	9,24E-04
CoastSIB	LamBC	0,85539216	1,20294E-05
CoastSIB	LamRT	0,875	4,28012E-06

**CoastSIB**	**LamSIB**	0,93872549	2,46438E-08
CoastSIB	Nanno	-0,110	6,73E-01
**CoastSIB**	SL	0,547	2,32E-02
CoastSIB	SST	0,412	1,01E-01
**DCA**	**BenthBC**	0,8595955	9,76599E-06
**DCA**	**BenthRT**	0,83578431	2,94017E-05
**DCA**	**BenthSIB**	0,50735294	0,037632881
**DCA**	bony	-0,216	4,06E-01
**DCA**	**CarBC**	0,375	0,138034844
**DCA**	**CarRT**	0,67401961	0,003007538
**DCA**	**CarSIB**	0,69362745	0,002014144
**DCA**	CO2	0,711	1,38E-03
**DCA**	**CoastBC**	0,57598039	0,015531766
**DCA**	**CoastRT**	0,69117647	0,002121119
**DCA**	**CoastSIB**	0,67892157	0,002728141
**DCA**	**DeepBC**	-0,4509804	0,069231986
**DCA**	**DeepRT**	-0,3946078	0,117002322
**DCA**	**DeepSIB**	-0,3161765	0,216326972
**DCA**	Dino	0,801	1,10E-04
**DCA**	DST	0,951	4,79E-09
**DCA**	Flood	0,944	1,34E-08
**DCA**	Foram	0,647	4,99E-03
**DCA**	FragInd	0,956	2,21E-09
**DCA**	**LamBC**	0,75735294	0,000430216
**DCA**	**LamRT**	0,82352941	4,85755E-05
**DCA**	**LamSIB**	0,70588235	0,001543353
**DCA**	Nanno	-0,497	4,24E-02
**DCA**	SL	0,652	4,57E-03
**DCA**	SST	0,218	4,00E-01
**DeepBC**	**BenthBC**	-0,2403434	0,352784473
**DeepBC**	**BenthRT**	-0,4387255	0,078116713
**DeepBC**	**BenthSIB**	-0,502451	0,039832968
**DeepBC**	bony	0,130	6,19E-01
**DeepBC**	**CarBC**	0,35784314	0,15846399
**DeepBC**	**CarRT**	0,0245098	0,925607776
**DeepBC**	**CarSIB**	-0,1029412	0,694204783
**DeepBC**	CO2	-0,277	2,82E-01
**DeepBC**	**CoastBC**	0,23039216	0,373664867
**DeepBC**	**CoastRT**	0,07843137	0,764779883
**DeepBC**	**CoastSIB**	-0,0098039	0,970210571
**DeepBC**	Dino	-0,132	6,13E-01
**DeepBC**	DST	-0,453	6,75E-02
**DeepBC**	Flood	-0,294	2,52E-01
**DeepBC**	Foram	-0,583	1,40E-02
**DeepBC**	FragInd	-0,451	6,92E-02
**DeepBC**	**LamBC**	-0,0392157	0,881213586
**DeepBC**	**LamRT**	-0,1372549	0,599369052
**DeepBC**	**LamSIB**	0,01960784	0,940458344
**DeepBC**	Nanno	-0,083	7,50E-01
**DeepBC**	SL	-0,424	8,98E-02
**DeepBC**	SST	-0,169	5,16E-01
**DeepRT**	**BenthBC**	-0,0380135	0,884831959
**DeepRT**	**BenthRT**	-0,1617647	0,535075506
**DeepRT**	**BenthSIB**	-0,0784314	0,764779883
**DeepRT**	bony	0,027	9,18E-01
**DeepRT**	**CarBC**	0,49509804	0,043314417
**DeepRT**	**CarRT**	0,14705882	0,573277291
**DeepRT**	**CarSIB**	-0,002451	0,992551189
**DeepRT**	CO2	-0,010	9,70E-01
**DeepRT**	**CoastBC**	0,3627451	0,152429892
**DeepRT**	**CoastRT**	0,18382353	0,480034401
**DeepRT**	**CoastSIB**	0,07352941	0,779127287
**DeepRT**	**DeepBC**	0,77941176	0,000225699

DeepRT	Dino	-0,037	8,89E-01
DeepRT	DST	-0,387	1,25E-01
DeepRT	Flood	-0,172	5,10E-01
DeepRT	Foram	-0,370	1,44E-01
DeepRT	FragInd	-0,385	1,27E-01
DeepRT	LamBC	0,15196078	0,560415506
DeepRT	LamRT	0,01715686	0,947890882
DeepRT	LamSIB	0,13970588	0,592801082
DeepRT	Nanno	0,210	4,19E-01
DeepRT	SL	-0,054	8,37E-01
DeepRT	SST	-0,196	4,51E-01
DeepSIB	BenthBC	0,04169222	0,873766741
DeepSIB	BenthRT	0,11764706	0,65293344
DeepSIB	BenthSIB	0,44117647	0,076276683
DeepSIB	bony	0,015	9,55E-01
DeepSIB	CarBC	0,10539216	0,687265839
DeepSIB	CarRT	0,01960784	0,940458344
DeepSIB	CarSIB	-0,0220588	0,933030449
DeepSIB	CO2	0,100	7,01E-01
DeepSIB	CoastBC	0,00490196	0,985102959
DeepSIB	CoastRT	-0,0318627	0,903376845
DeepSIB	CoastSIB	-0,0514706	0,844466903
DeepSIB	DeepBC	-0,0367647	0,888592868
DeepSIB	DeepRT	0,46323529	0,061113371
DeepSIB	Dino	-0,150	5,67E-01
DeepSIB	DST	-0,243	3,48E-01
DeepSIB	Flood	-0,191	4,62E-01
DeepSIB	Foram	0,044	8,66E-01
DeepSIB	FragInd	-0,255	3,23E-01
DeepSIB	LamBC	0,0122549	0,962767575
DeepSIB	LamRT	-0,0245098	0,925607776
DeepSIB	LamSIB	-0,0122549	0,962767575
DeepSIB	Nanno	0,589	1,29E-02
DeepSIB	SL	0,284	2,69E-01
DeepSIB	SST	-0,069	7,94E-01
LamBC	bony	0,069	7,94E-01
LamBC	CarBC	0,85539216	1,20294E-05
LamBC	CarRT	0,95588235	2,20565E-09
LamBC	CarSIB	0,88235294	2,77727E-06
LamBC	CO2	0,848	1,71E-05
LamBC	Dino	0,939	2,46E-08
LamBC	DST	0,733	8,19E-04
LamBC	Flood	0,777	2,43E-04
LamBC	Foram	0,637	5,93E-03
LamBC	FragInd	0,748	5,61E-04
LamBC	Nanno	-0,155	5,54E-01
LamBC	SL	0,613	8,92E-03
LamBC	SST	0,294	2,52E-01
LamRT	bony	0,039	8,81E-01
LamRT	CarBC	0,79166667	0,000152775
LamRT	CarRT	0,94607843	9,65394E-09
LamRT	CarSIB	0,89215686	1,48935E-06
LamRT	CO2	0,865	7,32E-06
LamRT	Dino	0,944	1,34E-08
LamRT	DST	0,797	1,30E-04
LamRT	Flood	0,819	5,88E-05
LamRT	Foram	0,679	2,73E-03
LamRT	FragInd	0,809	8,46E-05
LamRT	LamBC	0,98039216	5,40856E-12
LamRT	Nanno	-0,189	4,68E-01
LamRT	SL	0,676	2,87E-03
LamRT	SST	0,346	1,74E-01
LamSIB	bony	-0,267	3,00E-01

LamSIB	CarBC	0,69852941	0,001813432
LamSIB	CarRT	0,81862745	5,87601E-05
LamSIB	CarSIB	0,80147059	0,00010974
LamSIB	CO2	0,679	2,73E-03
LamSIB	Dino	0,743	6,38E-04
LamSIB	DST	0,652	4,57E-03
LamSIB	Flood	0,777	2,43E-04
LamSIB	Foram	0,456	6,59E-02
LamSIB	FragInd	0,696	1,91E-03
LamSIB	LamBC	0,85539216	1,20294E-05
LamSIB	LamRT	0,87990196	3,2179E-06
LamSIB	Nanno	-0,191	4,62E-01
LamSIB	SL	0,576	1,55E-02
LamSIB	SST	0,358	1,58E-01
ranged	BatSIB	0,54901961	0,022461152
ranged	Bboundary	0,86993931	5,6765E-06
ranged	BenthBC	0,96014733	1,04178E-09
ranged	BenthRT	0,85784314	1,06611E-05
ranged	BenthSIB	0,62254902	0,00760691
ranged	bony	0,020	9,40E-01
ranged	boundary	0,95833333	1,44699E-09
ranged	Brange	0,86764706	6,42647E-06
ranged	CarBC	0,78676471	0,000179117
ranged	CarRT	0,94117647	1,82756E-08
ranged	CarSIB	0,90686275	5,18287E-07
ranged	CO2	0,841	2,38E-05
ranged	CoastBC	0,89215686	1,48935E-06
ranged	CoastRT	0,96078431	9,24916E-10
ranged	CoastSIB	0,87990196	3,2179E-06
ranged	DeepBC	-0,122549	0,639378272
ranged	DeepRT	0,04166667	0,873843533
ranged	DeepSIB	0,01715686	0,947890882
ranged	Dino	0,926	9,33E-08
ranged	DST	0,806	9,24E-05
ranged	Flood	0,824	4,86E-05
ranged	Foram	0,674	3,01E-03
ranged	FragInd	0,801	1,10E-04
ranged	LamBC	0,95343137	3,28499E-09
ranged	LamRT	0,97794118	1,29914E-11
ranged	LamSIB	0,85784314	1,06611E-05
ranged	Nanno	-0,171	5,13E-01
ranged	Sboundary	0,96813725	1,99099E-10
ranged	SelSIB	0,93137255	5,64207E-08
ranged	SL	0,716	1,24E-03
ranged	Srange	0,99019608	3,06422E-14
ranged	SST	0,284	2,69E-01
sampled	BatSIB	0,62009804	0,007919521
sampled	Bboundary	0,79877361	0,000120405
sampled	BenthBC	0,88779906	1,97855E-06
sampled	BenthRT	0,81862745	5,87601E-05
sampled	BenthSIB	0,67401961	0,003007538
sampled	bony	-0,150	5,67E-01
sampled	boundary	0,84558824	1,90947E-05
sampled	Brange	0,84558824	1,90947E-05
sampled	CarBC	0,65931373	0,003988577
sampled	CarRT	0,83578431	2,94017E-05
sampled	CarSIB	0,88235294	2,77727E-06
sampled	CO2	0,752	4,92E-04
sampled	CoastBC	0,75	0,000525731
sampled	CoastRT	0,85784314	1,06611E-05
sampled	CoastSIB	0,92647059	9,32988E-08
sampled	DeepBC	-0,1960784	0,450704981
sampled	DeepRT	0,00980392	0,970210571

sampled	DeepSIB	0,12254902	0,639378272
sampled	Dino	0,767	3,26E-04
sampled	DST	0,721	1,10E-03
sampled	Flood	0,779	2,26E-04
sampled	Foram	0,652	4,57E-03
sampled	FragInd	0,748	5,61E-04
sampled	LamBC	0,83333333	3,26105E-05
sampled	LamRT	0,87990196	3,2179E-06
sampled	LamSIB	0,89705882	1,06622E-06
sampled	Nanno	-0,044	8,66E-01
sampled	ranged	0,91911765	1,86581E-07
sampled	Sboundary	0,85784314	1,06611E-05
sampled	SelSIB	0,97058824	1,10013E-10
sampled	SL	0,775	2,62E-04
sampled	Srange	0,89460784	1,26272E-06
sampled	SST	0,368	1,47E-01
Sboundary	BatSIB	0,42892157	0,085801019
Sboundary	Bboundary	0,90920314	4,31225E-07
Sboundary	BenthBC	0,91968135	1,77343E-07
Sboundary	BenthRT	0,7745098	0,000262068
Sboundary	BenthSIB	0,50490196	0,038721049
Sboundary	bony	0,029	9,11E-01
Sboundary	Brange	0,78921569	0,000165507
Sboundary	CarBC	0,84068627	2,37789E-05
Sboundary	CarRT	0,94117647	1,82756E-08
Sboundary	CarSIB	0,88970588	1,74986E-06
Sboundary	CO2	0,828	3,99E-05
Sboundary	CoastBC	0,95098039	4,79176E-09
Sboundary	CoastRT	0,97794118	1,29914E-11
Sboundary	CoastSIB	0,86764706	6,42647E-06
Sboundary	DeepBC	-0,0220588	0,933030449
Sboundary	DeepRT	0,15441176	0,554031999
Sboundary	DeepSIB	-0,0269608	0,918190901
Sboundary	Dino	0,934	4,33E-08
Sboundary	DST	0,740	6,80E-04
Sboundary	Flood	0,801	1,10E-04
Sboundary	Foram	0,632	6,45E-03
Sboundary	FragInd	0,757	4,30E-04
Sboundary	LamBC	0,98529412	6,34159E-13
Sboundary	LamRT	0,96813725	1,99099E-10
Sboundary	LamSIB	0,84803922	1,70628E-05
Sboundary	Nanno	-0,150	5,66E-01
Sboundary	SL	0,623	7,61E-03
Sboundary	SST	0,240	3,53E-01
SelSIB	BatSIB	0,53921569	0,02550368
SelSIB	Bboundary	0,84417241	2,03593E-05
SelSIB	BenthBC	0,90374021	6,57564E-07
SelSIB	BenthRT	0,81617647	6,44926E-05
SelSIB	BenthSIB	0,57107843	0,016646382
SelSIB	bony	-0,152	5,60E-01
SelSIB	Brange	0,80637255	9,23719E-05
SelSIB	CarBC	0,69117647	0,002121119
SelSIB	CarRT	0,87745098	3,71678E-06
SelSIB	CarSIB	0,91666667	2,31712E-07
SelSIB	CO2	0,735	7,70E-04
SelSIB	CoastBC	0,79656863	0,000129764
SelSIB	CoastRT	0,90441176	6,25179E-07
SelSIB	CoastSIB	0,95343137	3,28499E-09
SelSIB	DeepBC	-0,1029412	0,694204783
SelSIB	DeepRT	0,02696078	0,918190901
SelSIB	DeepSIB	0,05637255	0,829848584
SelSIB	Dino	0,799	1,19E-04
SelSIB	DST	0,725	9,80E-04

SelSIB	Flood	0,784	1,94E-04
SelSIB	Foram	0,564	1,84E-02
SelSIB	FragInd	0,772	2,82E-04
SelSIB	LamBC	0,86519608	7,32003E-06
SelSIB	LamRT	0,90441176	6,25179E-07
SelSIB	LamSIB	0,92892157	7,2878E-08
SelSIB	Nanno	-0,141	5,89E-01
SelSIB	Sboundary	0,88480392	2,38916E-06
SelSIB	SL	0,654	4,37E-03
SelSIB	Srange	0,92156863	1,49208E-07
SelSIB	SST	0,355	1,62E-01
Srange	BatSIB	0,50735294	0,037632881
Srange	Bboundary	0,84908039	1,62569E-05
Srange	BenthBC	0,97118351	9,45355E-11
Srange	BenthRT	0,86519608	7,32003E-06
Srange	BenthSIB	0,59313725	0,012085076
Srange	bony	0,025	9,26E-01
Srange	Brange	0,84558824	1,90947E-05
Srange	CarBC	0,74754902	0,000561238
Srange	CarRT	0,92647059	9,32988E-08
Srange	CarSIB	0,88970588	1,74986E-06
Srange	CO2	0,841	2,38E-05
Srange	CoastBC	0,87254902	4,91459E-06
Srange	CoastRT	0,94852941	6,8596E-09
Srange	CoastSIB	0,8627451	8,31713E-06
Srange	DeepBC	-0,1691176	0,516417623
Srange	DeepRT	-0,0196078	0,940458344
Srange	DeepSIB	-0,0122549	0,962767575
Srange	Dino	0,929	7,29E-08
Srange	DST	0,831	3,61E-05
Srange	Flood	0,843	2,13E-05
Srange	Foram	0,676	2,87E-03
Srange	FragInd	0,838	2,65E-05
Srange	LamBC	0,95833333	1,44699E-09
Srange	LamRT	0,9877451	1,62759E-13
Srange	LamSIB	0,85539216	1,20294E-05
Srange	Nanno	-0,199	4,44E-01
Srange	Sboundary	0,96323529	5,74099E-10
Srange	SL	0,694	2,01E-03
Srange	SST	0,262	3,09E-01

Table S6: Summary of statistical outcomes for the best-supported models linking abiotic predictors to diversity patterns (SQS) and faunal turnover (DCA) across varying taxonomic and ecologic groups and diversity metrics.

	Sampled in Bin Neoselachians					Sampled in Bin Selachians					Sampled in Bin Batoids				
	y1 ~ x1 + x2 + x6					y2 ~ x1 + x2 + x6					y3 ~ x1 + x2 + x5 + x6				
Predictors	***Estimates***	***standardized std. Error***	***t-value***	***p***	***df***	***Estimates***	***std. Error***	***standardized std. Error***	***p***	***df***	***Estimates***	***std. Error***	***standardized std. Error***	***t-value***	***df***
**(Intercept)**	-85,428	0,129	-2,514	2,5894E-02	13	-66,619	25,923	0,146	2,3298E-02	13	-17,392	19,972	0,161	-0,871	12
**x1 SL**	0,229	0,168	2,796	1,5159E-02	13	0,115	0,062	0,190	8,7642E-02	13	0,091	0,047	0,241	1,930	12
**x2 SST**	1,399	0,139	1,834	8,9599E-02	13	0,910	0,582	0,157	1,4168E-01	13	0,648	0,389	0,176	1,668	12
**x6 FragInd**	156,444	0,164	2,425	3,0597E-02	13	127,019	49,209	0,186	2,2804E-02	13	-88,961	49,792	0,315	-1,787	12
**x5 Flood**											0,839	0,423	0,358	1,984	12
**x4 CO2**															
**x3 DST**															
**Observations**	17					17					17				
**R2**	0,771					0,706					0,670				
**R2 Adj,**	0,718					0,638					0,560				
**AIC**	67,150					57,950					44,280				
**F-statistic**	14,580					10,420					6,096				
**p-value**	1,89E-04					9,15E-04					6,46E-03				

	Boundary Crossers Neoselachians					Boundary Crossers Selachians						Boundary Crossers Batoids					
	y4 ~ x4 + x6					y5 ~ x4 + x6						y6 ~ x6					
Predictors	***Estimates***	***std. Error***	***standardized std. Error***	***t-value***	***p***	***Estimates***	***std. Error***	***standardized std. Error***	***t-value***	***p***	***df***	***Estimates***	***std. Error***	***standardized std. Error***	***t-value***	***p***	***df***
**(Intercept)**	-106,188	32,132	0,121	-3,305	5,2122E-03	-85,171	22,239	0,110	-3,830	1,8394E-03	14	-8,548	6,247	0,194	-1,368	1,9136E-01	15
**x1 SL**																	
**x2 SST**																	
**x6 FragInd**	259,204	68,928	0,162	3,761	2,1090E-03	203,814	47,706	0,148	4,272	7,7402E-04	14	39,412	12,400	0,200	3,178	6,2340E-03	15
**x5 Flood**																	
**x4 CO2**	0,015	0,007	0,162	2,207	4,4482E-02	0,011	0,005	0,148	2,428	2,9227E-02	14						
**x3 DST**																	
**Observations**	17					17						17					
**R2**	0,784					0,821						0,402					
**R2 Adj,**	0,753					0,795						0,363					
**AIC**	66,710					54,200						16,670					
**F-statistic**	25,380					32,060						10,100					
**p-value**	2,21E-05					5,94E-06						6,23E-03					

	Range Through Neoselachians							
	y7 ~ x4 + x6							
Predictors	***Estimates***	***std. Error***	***standardized std. Error***	***t-value***	***p***	***df***	***Estimates***	***std. Error***
**(Intercept)**	-143,202	33,220	0,095	-4,311	7,1849E-04	14	-110,259	25,162
**x1 SL**								
**x2 SST**								
**x6 FragInd**	334,681	71,263	0,128	4,696	3,4350E-04	14	255,672	53,977
**x5 Flood**								
**x4 CO2**	0,023	0,007	0,128	3,320	5,0591E-03	14	0,016	0,005
**x3 DST**								
**Observations**	17						17	
**R2**	0,866						0,859	
**R2 Adj,**	0,847						0,839	
**AIC**	67,840						58,400	
**F-statistic**	45,420						42,680	
**p-value**	7,57E-07						1,10E-06	

Dependent Variable	***Diversity Approach***
**y1**	Sampled in Bin Neoselachians
**y2**	Sampled in Bin Selachians
**y3**	Sampled in Bin Batoids
**y4**	Boundary Crossers Neoselachians
**y5**	Boundary Crossers Selachians
**y6**	Boundary Crossers Batoids
**y7**	Range Through Neoselachians
**y8**	Range Through Selachians
**y9**	Range Through Batoids
**y10**	Detrended Correspondence Analysis Axis 1 Neoselachians
**y11**	Sampled in Bin Deep Sea
**y12**	Sampled in Bin Coastal
**y13**	Sampled in Bin Benthic
**y14**	Range Through Deep Sea
**y15**	Range Through Coastal
**y16**	Range Through Benthic
**y17**	Boundary Crossers Deep Sea
**y18**	Boundary Crossers Coastal
**y19**	Boundary Crossers Benthic
**y20**	Range Through Lamniformes
**y21**	Range Through Carcharhiniform
**y22**	Boundary Crossers Lamniformes
**y23**	Boundary Crossers Carcharhiniform

Predictors	
**x1**	Sea Level
**x2**	Sea Surface Temperature
**x3**	Deap Sea Temperature
**x4**	Atmospheric CO2 Concentration
**x5**	Flooded Continental Area
**x6**	Continental Fragmentation

Range Through Selachians				Range Through Batoids					
y8 ~ x4 + x6				y9 ~ x1 + x4 + x5					
standardized std. Error	***t-value***	***p***	***df***	***Estimates***	***std. Error***	***standardized std. Error***	***t-value***	***p***	***df***
0,097	-4,382	6,2622E-04	14	-9,654	7,883	0,103	-1,225	2,4244E-01	13
				0,054	0,025	0,186	2,169	4,9233E-02	13
0,131	4,737	3,1836E-04	14						
				0,310	0,121	0,149	2,554	2,4016E-02	13
0,131	3,017	9,2352E-03	14	0,003	0,002	0,168	1,484	1,6164E-01	13
				17					
				0,854					
				0,821					
				15,680					
				25,390					
				1,04E-05					

Detrended Correspondence Analysis Axis 1 Neoselachians						Sampled in Bin Deep Sea					
y10 ~ x1 + x2 + x5 + x6						y11 ~ x1 + x6					
Estimates	***std. Error***	***standardized std. Error***	***t-value***	***p***	***df***	***Estimates***	***std. Error***	***standardized std. Error***	***t-value***	***p***	***df***
-25,017	1,453	0,045	-17,219	7,9506E-10	12	48,962	16,107	0,201	3,040	8,8249E-03	14
-0,005	0,003	0,068	-1,365	1,9743E-01	12	0,110	0,041	0,256	2,703	1,7154E-02	14
0,049	0,028	0,050	1,747	1,0610E-01	12						
23,104	3,622	0,089	6,378	3,5131E-05	12	-89,785	32,876	0,256	-2,731	1,6238E-02	14
0,155	0,031	0,100	5,045	2,8690E-04	12						
17						17					
0,974						0,399					
0,965						0,314					
-44,830						43,510					
112,600						4,656					
2,09E-09						2,82E-02					

Sampled in Bin Coastal						Sampled in Bin Benthic					
y12 ~ x2 + x6						y13 ~ x1 + x4 + x6					
Estimates	***std. Error***	***standardized std. Error***	***t-value***	***p***	***df***	***Estimates***	***std. Error***	***standardized std. Error***	***t-value***	***p***	***df***
-42,530	10,782	0,160	-3,944	1,4680E-03	14	16,706	8,988	0,155	1,859	8,5862E-02	13
						0,077	0,028	0,255	2,718	1,7569E-02	13
0,572	0,290	0,168	1,971	6,8769E-02	14						
81,219	20,802	0,168	3,904	1,5881E-03	14	-33,179	19,040	0,213	-1,743	1,0500E-01	13
						0,003	0,002	0,270	1,362	1,9620E-01	13
17						17					
0,621						0,668					
0,567						0,592					
34,450						23,130					
11,470						8,728					
1,12E-03						1,97E-03					

Range Through Deep Sea							
y14 ~ x2 + x3 + x4 + x6							
Estimates	***std. Error***	***std. Beta***	***standardized std. Error***	***t-value***	***p***	***df***	***Estimates***
-12,758	16,036	0,000	0,194	-0,796	4,4172E-01	12	-49,399
-0,396	0,250	-0,389	0,245	-1,585	1,3886E-01	12	
75,661	34,142	1,036	0,468	2,216	4,6769E-02	12	119,972
0,006	0,002	0,803	0,351	2,291	4,0866E-02	12	0,007
-0,747	0,226	-1,759	0,531	-3,312	6,1962E-03	12	
17							17
0,519							0,776
0,359							0,744
24,480							41,000
3,236							24,190
5,10E-02							2,87E-05

Range Through Coastal					Range Through Benthic					
y15 ~ x4 + x6					y16 ~ x4 + x5					
std. Error	standardized std. Error	t-value	p	df	Estimates	std. Error	standardized std. Error	t-value	p	df
15,083	0,123	-3,275	5,5287E-03	14	-15,264	5,419	0,111	-2,816	1,3729E-02	14
32,355	0,166	3,708	2,3400E-03	14						
					0,300	0,086	0,144	3,496	3,5921E-03	14
0,003	0,166	2,114	5,2950E-02	14	0,004	0,001	0,144	3,492	3,5907E-03	14
					17					
					0,816					
					0,789					
					7,030					
					30,940					
					7,27E-06					

Boundary Crossers Deep Sea						Boundary Crossers Coastal					
y17 ~ x3 + x6						y18 ~ x4 + x5 + x6					
Estimates	std. Error	standardized std. Error	t-value	p	df	Estimates	std. Error	standardized std. Error	t-value	p	df
-32,340	9,905	0,140	-3,265	5,6411E-03	14	-26,641	15,019	0,149	-1,774	9,9501E-02	13
104,530	22,149	0,336	4,719	3,2894E-04	14	134,450	46,098	0,303	2,917	1,2018E-02	13
						-0,466	0,332	0,291	-1,402	1,8422E-01	13
						0,005	0,003	0,203	1,770	1,0017E-01	13
-0,738	0,129	0,336	-5,728	5,2238E-05	14						
17						17					
0,708						0,695					
0,666						0,624					
8,540						39,740					
16,950						9,863					
1,82E-04						1,16E-03					

Boundary Crossers Benthic						Range Through Lamniformes					
y19 ~ x4 + x5						y20 ~ x4 + x6					
Estimates	std. Error	standardized std. Error	t-value	p	df	Estimates	std. Error	standardized std. Error	t-value	p	df
-10,924	3,843	0,106	-2,843	1,3043E-02	14	-34,947	7,145	0,089	-4,891	2,3818E-04	14
						83,703	15,327	0,120	5,461	8,3863E-05	14
0,236	0,061	0,137	3,873	1,6882E-03	14						
0,003	0,001	0,137	3,557	3,1572E-03	14	0,005	0,002	0,120	3,086	8,0616E-03	14
17						17					
0,833						0,882					
0,810						0,865					
-4,650						15,590					
35,040						52,170					
3,55E-06						3,25E-07					

Range Through Carcharhiniformes						
y21 ~ x4 + x6						
Estimates	std. Error	standardized std. Error	t-value	p	df	Estimates
-19,560	12,838	0,135	-1,524	1,4989E-01	14	-26,894
49,471	27,540	0,182	1,796	9,4044E-02	14	66,032
0,009	0,003	0,182	3,313	5,1247E-03	14	0,004
17						17
0,728						0,843
0,689						0,821
35,520						14,560
18,690						37,670
1,12E-04						2,32E-06

Boundary Crossers Lamniformes					Boundary Crossers Carcharhiniformes					
y22 ~ x4 + x6					y23 ~ x4 + x5 + x6					
std. Error	*standardized std. Error*	*t-value*	*p*	*df*	*Estimates*	*std. Error*	*standardized std. Error*	*t-value*	*p*	*df*
6,931	0,103	-3,880	1,6661E-03	14	-2,420	14,859	0,182	-0,163	8,7314E-01	13
14,869	0,138	4,441	5,5911E-04	14	72,976	45,608	0,371	1,600	1,3359E-01	13
					-0,433	0,328	0,356	-1,319	2,0995E-01	13
0,001	0,138	2,845	1,2981E-02	14	0,007	0,003	0,248	2,318	3,7408E-02	13
					17					
					0,543					
					0,437					
					39,380					
					5,141					
					1,46E-02					

Table S7: Summary of statistical outcomes for the best-supported models linking biotic predictors to diversity patterns (SQS) and faunal turnover (DCA) across varying taxonomic and ecologic groups and diversity metrics.

	Sampled in Bin Neoselachians						Sampled in Bin Selachians					
	y1 ~ x7 + x8 + x9						y2 ~ x8					
Predictors	*Estimates*	*std. Error*	*standardized std. Error*	*t-value*	*p*	*df*	*Estimates*	*std. Error*	*standardized std. Error*	*t-value*	*p*	*df*
(Intercept)	-11.0642	7,886	0.1355	-1.4030	1.8406e-01	13	-2.6649	3,617	0.1451	-0.7367	4.7265e-01	15
x7 Bony	-0.0287	0.0199	0.1483	-1.4465	1.7172e-01	13						
x8 Dino	0.2027	0.0331	0.1542	6.1313	3.5941e-05	13	0.1178	0.0216	0.1496	5,451	6.6976e-05	15
x9 Nanno	0.1540	0.0938	0.1551	1,642	1.2446e-01	13						
x10 Foram												
Observations	17						17					
R2	0.746						0.665					
R2 Adj.	0.688						0.642					
AIC	68.87						56.2					
F-statistic	12,75						29.71					
p-value	0.0003596						6.698e-05					

	Sampled in Bin Batoids							
	y3 ~ x7 + x10							
Predictors	*Estimates*	*std. Error*	*standardized std. Error*	*t-value*	*p*	*df*	*Estimates*	*std. Error*
(Intercept)	1.289	3,262	0.1826	0.3953	6.9860e-01	14	1.765	3,613
x7 Bony	-0.0243	0.0107	0.1998	-2.2620	4.0135e-02	14		
x8 Dino							0.2464	0.0260
x9 Nanno								
x10 Foram	0.1727	0.0479	0.1998	3,602	2.8887e-03	14	-0.1082	0.0726
Observations	17						17	
R2	0.504						0.912	
R2 Adj.	0.433						0.899	
AIC	47.22						51.53	
F-statistic	7.111						72.1	
p-value	0.007392						4.249e-08	

Dependent Variable	*Diversity Approach*
y1	Sampled in Bin Neoselachians
y2	Sampled in Bin Selachians
y3	Sampled in Bin Batoids
y4	Boundary Crossers Neoselachians
y5	Boundary Crossers Selachians
y6	Boundary Crossers Batoids
y7	Range Through Neoselachians
y8	Range Through Selachians
y9	Range Through Batoids
y10	Detrended Correspondence Analysis Axis 1 Neoselachians
y11	Sampled in Bin Deep Sea
y12	Sampled in Bin Coastal
y13	Sampled in Bin Benthic
y14	Range Through Deep Sea
y15	Range Through Coastal
y16	Range Through Benthic
y17	Boundary Crossers Deep Sea
y18	Boundary Crossers Coastal
y19	Boundary Crossers Benthic
y20	Range Through Lamniformes
y21	Range Through Carcharhiniform
y22	Boundary Crossers Lamniformes
y23	Boundary Crossers Carcharhiniform

Predictors	
x7	Bony fish diversity
x8	Dinoflagellate diversity
x9	Calcaleous nannoplankton diversity
x10	Planktic foraminiferan diversity

oundary Crossers Neoselachians				Boundary Crossers Selachians						Boundary Crossers Batoids						Range Through Neoselachians			
y4 ~ x8 + x10				y5 ~ x8 + x10						y6 ~ x8 + x9 + x10						y7 ~ x7 + x8			
standardized std. Error	*t-value*	*p*	*df*	*Estimates*	*std. Error*	*standardized std. Error*	*t-value*	*p*	*df*	*Estimates*	*std. Error*	*standardized std. Error*	*t-value*	*p*	*df*	*Estimates*	*std. Error*	*standardized std. Error*	*t-value*
0.0771	0.4886	6.3268e-01	14	-0.1464	2,557	0.0718	-0.0573	9.5515e-01	14	5.687	1.1759	0.1291	4,836	3.2531e-04	13	-2.340	4,754	0.0797	-0.4922
																-0.023	0.0159	0.0835	-1.4706
0.1125	9,474	1.8173e-07	14	0.1863	0.0184	0.1048	10.1204	8.0525e-08	14	0.0458	0.0079	0.2337	5,795	6.2303e-05	13	0.294	0.0254	0.0835	11,563
										0.0442	0.0158	0.1661	2,787	1.5394e-02	13				
0.1125	-1.4912	1.5808e-01	14	-0.0729	0.0513	0.1048	-1.4192	1.7773e-01	14	-0.0570	0.0209	0.2212	-2.7282	1.7241e-02	13				
				17						17						17			
				0.923						0.770						0.905			
				0.912						0.717						0.892			
				39.77						4,45						61.98			
				84.25						14,50						67.03			
				1.563e-08						0.0001936						6.756e-08			

		Range Through Selachians						Range Through Batoids						Detrended Correspondence Analysis Axis 1 Neoselachians						
		y8 ~ x7 + x8						y9 ~ x7 + x8						y10 ~ x7 + x8 + x9 + x10						
p	*df*	*Estimates*	*std. Error*	*standardized std. Error*	*t-value*	*p*	*df*	*Estimates*	*std. Error*	*standardized std. Error*	*t-value*	*p*	*df*	*Estimates*	*std. Error*	*standardized std. Error*	*t-value*	*p*	*df*	*Estimates*
6.3019e-01	14	-2.6622	3,592	0.0817	-0.7411	4.7086e-01	14	8.419	1,687	0.1454	4,989	1.9840e-04	14	-3.1670	0.5057	0.0835	-6.2627	4.1741e-05	12	0.3316
1.6352e-01	14	-0.0165	0.0120	0.0856	-1.3790	1.8952e-01	14	-0.0112	0.0056	0.1523	-1.9826	6.7391e-02	14	-0.0028	0.0013	0.0929	-2.1603	5.1685e-02	12	
1.5033e-08	14	0.2159	0.0192	0.0856	Nov.19	2.1272e-08	14	0.0489	0.0090	0.1523	5,427	8.9143e-05	14	0.0069	0.0034	0.1512	2.0376	6.4260e-02	12	
														-0.0334	0.0068	0.1089	-4.8763	3.8091e-04	12	0.1168
														0.0321	0.0091	0.1455	3,542	4.0594e-03	12	
		17						17						17						17
		0.901						0.686						0.911						0.349
		0.887						0.641						0.882						0.306
		52.45						26.76						-23,90						42.87
		63.51						15.26						30.76						8.054
		9.508e-08						0.0003042						3.183e-06						0.01247

Sampled in Bin Deep Sea					Sampled in Bin Coastal						Sampled in Bin Benthic						Range Through Deep Sea			
y11 ~ x9					y12 ~ x7 + x8 + x9						y13 ~ x8 + x9						y14 ~ x8 + x9 + x10			
std. Error	*standardized std. Error*	*t-value*	*p*	*df*	*Estimates*	*std. Error*	*standardized std. Error*	*t-value*	*p*	*df*	*Estimates*	*std. Error*	*standardized std. Error*	*t-value*	*p*	*df*	*Estimates*	*std. Error*	*standardized std. Error*	*t-value*
2.1317	0.2021	0.1556	8.7846e-01	15	-1.6403	2,618	0.1427	-0.6266	5.4179e-01	13	-4.2755	2,619	0.1993	-1.6325	1.2486e-01	14	10.693	1,728	0.1613	6.1888
					-0.0085	0.0066	0.1562	-1.2932	2.1844e-01	13										
					0.0624	0.0110	0.1624	5,689	7.4314e-05	13	0.0311	0.0107	0.2183	2,922	1.1154e-02	14	0.0487	0.0116	0.2920	4.1894
0.0412	0.2083	2,838	1.2468e-02	15	0.0426	0.0311	0.1633	1,37	1.9401e-01	13	0.0602	0.0301	0.2183	2.0035	6.4870e-02	14	0.0898	0.0233	0.2076	3,855
																	-0.1361	0.0307	0.2764	-4.4374
					17						17						17			
					0.719						0.409						0.641			
					0.654						0.325						0.558			
					31.38						30.93						17.53			
					11,07						4,85						7.722			
					0.0006938						2,51E-02						0.003259			

		Range Through Coastal						Range Through Benthic						Boundary Crossers Deep Sea						
		y15 ~ x8 + x10						y16 ~ x7 + x8 + x10						y17 ~ x7 + x8 + x9 + x10						
p	*df*	*Estimates*	*std. Error*	*standardized std. Error*	*t-value*	*p*	*df*	*Estimates*	*std. Error*	*standardized std. Error*	*t-value*	*p*	*df*	*Estimates*	*std. Error*	*standardized std. Error*	*t-value*	*p*	*df*	*Estimates*
3.2772e-05	13	0.6380	1,662	0.0770	0.3839	7.0680e-01	14	1.543	1,307	0.1421	1.1808	2.5882e-01	13	12,654	1,513	0,1551	8,361	2,39E-06	12	0,5507
								-0.0066	0.0042	0.1561	-1.5669	1.4115e-01	13	0,0076	0,0039	0,1727	1,952	7,47E-02	12	0,0074
1.0603e-03	13	0.1145	0.0120	0.1123	4,189	1.6117e-07	14	0.0174	0.0090	0.2082	1,93	7.5698e-02	13	0,0339	0,0101	0,2809	3,356	5,71E-03	12	0,105
1.9893e-03	13													0,0398	0,0205	0,2023	1,942	7,59E-02	12	0,034
6.7001e-04	13	-0.0540	0.0334	0.1123	-1.6179	1.2798e-01	14	0.0671	0.0262	0.2175	2,559	2.3779e-02	13	-0,1371	0,0271	0,2704	-5,0582	2,81E-04	12	-0,111
		17						17						17						17
		0.912						0.721						0,69						0,90
		0.899						0.657						0,59						0,87
		25,12						16,06						13,37						22,66
		72.36						11,20						6,779						27,20
		4.153e-08						0.0006588						4,30E-03						6,16E-06

Boundary Crossers Coastal					Boundary Crossers Benthic						Range Through Lamniformes						Range Through Carcharhiniform			
y18 ~ x7 + x8 + x9 + x10					y19 ~ x7 + x8 + x10						y20 ~ x8						y21 ~ x7 + x8 + x9 + x10			
std. Error	***standardized std. Error***	***t-value***	***p***	***df***	***Estimates***	***std. Error***	***standardized std. Error***	***t-value***	***p***	***df***	***Estimates***	***std. Error***	***standardized std. Error***	***t-value***	***p***	***df***	***Estimates***	***std. Error***	***standardized std. Error***	***t-value***
1,989	0,0883	0,2768	7,87E-01	12	1,955	0,7727	0,1126	2,53	2,51E-02	13	-0,3984	0,9115	0,076	-0,4371	6,68E-01	15	-4,8294	1,653	0,074	-2,922
0,0051	0,0983	1,46	1,70E-01	12	-0,0043	0,0025	0,1237	-1,7438	1,05E-01	13							0,0066	0,0042	0,0824	1,553
0,0133	0,1599	7,912	4,21E-06	12	0,0157	0,0053	0,1649	2,945	1,14E-02	13	0,0662	0,0054	0,0784	12,1585	3,61E-09	15	0,0923	0,011	0,134	8,371
0,0269	0,1151	1,261	2,31E-01	12													0,0571	0,0224	0,0965	2,551
0,0356	0,1539	-3,1155	8,93E-03	12	0,0484	0,0155	0,1723	3,1172	8,17E-03	13							-0,0419	0,0296	0,129	-1,4147
					17						17						17			
					0,83						0,91						0,93			
					0,78						0,90						0,91			
					-1,8						9,34						16,36			
					20,41						147,80						39,99			
					3,38E-05						3,61E-09						7,60E-07			

		Boundary Crossers Lamniformes						Boundary Crossers Carcharhiniform					
		y22 ~ x8						y23 ~ x7 + x8 + x9 + x10					
p	*df*	*Estimates*	*std. Error*	*standardized std. Error*	*t-value*	*p*	*df*	*Estimates*	*std. Error*	*standardized std. Error*	*t-value*	*p*	*df*
1,28E-02	12	0,0733	0,7427	0,0735	0,0987	9,23E-01	15	-3,6762	01,01,5892	0,0873	-2,3133	3,92E-02	12
1,46E-01	12							0,0093	0,0041	0,0971	2,281	4,16E-02	12
2,36E-06	12	0,056	0,0044	0,0757	01,12,6207	2,16E-09	15	0,0912	0,0106	0,158	8,608	1,76E-06	12
2,54E-02	12							0,086	0,0215	0,1138	3,999	1,76E-03	12
1,83E-01	12							-0,1102	0,0285	0,1521	-3,8733	2,21E-03	12
		17						17					
		0,91						0,90					
		0,91						0,87					
		2,38						15,03					
		159,3						27,91					
		2,16E-09						5,37E-06					
